# Phase II study of novel orally PI3Kα/δ inhibitor TQ-B3525 in relapsed and/or refractory follicular lymphoma

**DOI:** 10.1038/s41392-024-01798-0

**Published:** 2024-04-17

**Authors:** Huaqing Wang, Jifeng Feng, Yanyan Liu, Zhengzi Qian, Da Gao, Xuehong Ran, Hui Zhou, Lihong Liu, Binghua Wang, Meiyun Fang, Hebing Zhou, Zhenqian Huang, Shi Tao, Zhuowen Chen, Liping Su, Hang Su, Yu Yang, Xiaobao Xie, Huijing Wu, Ping Sun, Guoyu Hu, Aibin Liang, Zhiming Li

**Affiliations:** 1 Department of Oncology, Tianjin Union Medical Center of Nankai University, Tianjin, 300121 PR China; 2 The Institute of Translational Medicine, Tianjin Union Medical Center of Nankai University, Tianjin, 300121 PR China; 3 Department of Medical Oncology, Jiangsu Cancer Hospital, The Affiliated Cancer Hospital of Nanjing Medical University, Nanjing, 210009 PR China; 4 Department of Medical Oncology, Henan Cancer Hospital, The Affiliated Cancer Hospital of Zhengzhou University, Zhengzhou, 450003 PR China; 5 Department of Medical Oncology, Tianjin Medical University Cancer Institute and Hospital, Tianjin, 300060 PR China; 6 Department of Hematology, The Affiliated Hospital of Inner Mongolia Medical College, 010050 Hohhot, PR China; 7 Department of Hematology, Weifang People’s Hospital, The First Affiliated Hospital of Weifang Medical University, 261000 Weifang, PR China; 8 Department of Lymphoma & Hematology, Hunan Cancer Hospital, The Affiliated Cancer Hospital of Xiangya School of Medicine, Central South University, 410013 Changsha, PR China; 9 Department of Hematology, The Fourth Hospital of Hebei Medical University and Hebei Tumor Hospital, 050011 Shijiazhuang, PR China; 10 Department of Lymphoma, Weihai Central Hospital, 264400 Weihai, PR China; 11 Department of Hematology and Rheumatology, The Affiliated Zhongshan Hospital of Dalian University, 116001 Dalian, PR China; 12 Department of Hematology, Beijing Luhe Hospital, 101199 Beijing, PR China; 13 Department of Hematology, The First Affiliated Hospital of Guangzhou Medical University, 510120 Guangzhou, PR China; 14 Department of Hematology, The First Affiliated Hospital of Hainan Medical College, 570102 Haikou, PR China; 15 Department of Hematology, The First People’s Hospital of Foshan, 528000 Foshan, PR China; 16 Department of Hematology, Shanxi Cancer Hospital, 030013 Taiyuan, PR China; 17 Department of Lymphoma, Senior Department of Hematology, The Fifth Medical Center of Chinese PLA General Hospital, 100039 Beijing, PR China; 18 Department of Lymphoma and Head and Neck Cancer, Fujian Cancer Hospital, 350014 Fuzhou, PR China; 19 Department of Hematology, The First People’s Hospital of Changzhou, The Third Affiliated Hospital of Soochow University, 213003 Changzhou, PR China; 20 Department of Medical Oncology, Hubei Cancer Hospital Affiliated to Tongji Medical College, Huazhong University of Science and Technology, 430079 Wuhan, PR China; 21 Department of Medical Oncology, Yantai Yuhuangding Hospital, 264000 Yantai, PR China; 22 Department of Hematology, Zhuzhou Central Hospital, 412007 Zhuzhou, PR China; 23 Department of Hematology, Tongji Hospital of Tongji University, Shanghai, 200333 PR China; 24 Department of Medical Oncology, State Key Laboratory of Oncology in South China, Collaborative Innovation Center for Cancer Medicine, Guangdong Provincial Clinical Research Center for Cancer, Sun Yat-sen University Cancer Center, 510060 Guangzhou, PR China

**Keywords:** Cancer therapy, Drug development

## Abstract

This registration study assessed clinical outcomes of TQ-B3525, the dual phosphatidylinositol-3-kinase (PI3K) α/δ inhibitor, in relapsed and/or refractory follicular lymphoma (R/R FL). This phase II study (ClinicalTrials.gov NCT04324879. Registered March 27, 2020) comprised run-in stage and stage 2. R/R FL patients after ≥2 lines therapies received oral 20 mg TQ-B3525 once daily in a 28-day cycle until intolerable toxicity or disease progression. Primary endpoint was independent review committee (IRC)-assessed objective response rate (ORR). Based on results (ORR, 88.0%; duration of response [DOR], 11.8 months; progression-free survival [PFS], 12.0 months) in 25 patients at run-in stage, second stage study was initiated and included 82 patients for efficacy/safety analysis. Patients received prior-line (median, 3) therapies, with 56.1% refractory to previous last therapies; 73.2% experienced POD24 at baseline. At stage 2, ORR was 86.6% (71/82; 95% CI, 77.3–93.1%), with 28 (34.2%) complete responses. Disease control rate was 95.1% due to 7 (8.5%) stable diseases. Median time to response was 1.8 months. Among 71 responders, median DOR was not reached; 18-month DOR rate was 51.6%. with median follow-up of 13.3 months, median PFS was 18.5 (95% CI, 10.2-not estimable) months. Median overall survival (OS) was not reached by cutoff date; 24-month OS rate was estimated as 86.1%. Response rates and survival data were consistent across all subgroups. Grade 3 or higher treatment-related adverse events were observed in 63 (76.8%) cases, with neutropenia (22.0%), hyperglycemia (19.5%), and diarrhea (13.4%) being common. TQ-B3525 showed favorable efficacy and safety for R/R FL patients after ≥2 lines prior therapies.

## Introduction

Follicular lymphoma (FL) ranks among the most prevalent indolent non-Hodgkin lymphoma (NHL) and is incurable by a continuous pattern of relapse.^1^ Some patients progressed within 2 years (POD24) or relapsed after an initial response to first-line anti-CD20 monoclonal antibody-based chemotherapy regimens and eventual development of refractory disease^2^; generally, patients with POD24 or refractory FL had poor prognosis, which challenged positive clinical treatment.^3^ Despite available treatments (including chemoimmunotherapy, and targeted and cellular therapies) for subsequent relapses, successive relapses may lead to declining response rates and survival, progressively shortening remissions, and increased risk of cumulative toxicity.^4–8^ Thus, given the long natural history of relapsed and/or refractory (R/R) FL, paramount therapeutic focus for R/R FL is on increased objective response rates (ORR), prolonged survival, and reducing toxicities, ultimately improving patients’ quality of life.^9,10^

The dysregulation of PI3K signaling in hematological malignancies underscores the potential significance of PI3K inhibitor development.^11^ Several phosphatidylinositol 3-kinase (PI3K) inhibitors with idelalisib,^12^ copanlisib,^13^ duvelisib,^14^ and umbralisib^15^ target the abnormal PI3K pathways that drove lymphoma progression and are approved by FDA for third- or later-line R/R FL (Supplementary Table S1). Nonetheless, limited ORR ranging from 45.3%-59.0% and complete response rate (CRR; 1.6%-6.0%), and tolerability issues with high frequencies of serious toxicities (hepatic and gastrointestinal toxicity, colitis, pneumonitis, and infection) reported in these agents compromise treatment.^12,14–16^ Currently, the investigations on structural optimization, effective toxicity management, and predictive biomarkers partly mitigated the toxicities limiting the PI3K inhibitor development^17,18^; while there remains an unmet need for new PI3K inhibitors in balancing the enhanced activity with mitigated toxicities.

Preclinical evidence suggested that simultaneous blockade of PI3Kα and PI3Kδ could eliminate constitutive activation of PI3K and compensatory signaling-related mechanisms of resistance, thus increasing the activity.^19,20^ Considering the functionally dominant role of PI3Kδ in lymphocytes, isoform-specific inhibitors may result in reduced toxicity than less specific pan-PI3K inhibitors.^21^ Besides, the therapeutic effects of PI3Kδ blockade on FL arises from causing a less supportive and tolerogenic immune microenvironment and subsequently interfering with the tumor-promoting micro-environmental crosstalk.^22^ Thus, all these rationales suggested that dual blockade of PI3Kα/δ may be an attractive target. The mode of intravenous infusion of copanlisib, a potent pan-PI3K inhibitor predominantly targeting both isoforms of PI3K-α and PI3K-δ, may lead to high-risk hyperglycemia (41% grade ≥3) and hypertension (24% grade ≥3).^13^ Consequently, a need remains for oral improved PI3Kα/δ inhibitor that ideally optimize safety.

TQ-B3525, a selective orally PI3K α/δ inhibitor, is newly developed by China. Preclinical characterization with kinase activity indicated that the TQ-B3525 half maximal inhibitory concentration (IC_50_; unpublished data) of each class I PI3K isoform was much lower than idelalisib and duvelisib, and comparable with copanlisib.^23–25^ More thrillingly, TQ-B3525 has shown preliminary promising clinical activities and favorable safety (10.0% grade ≥3 hyperglycemia and 3.8% grade ≥3 hypertension) in advanced malignancies, especially R/R lymphoma.^26^

Although tazemetostat (enhancer of Zeste homolog 2 [EZH2] inhibitor), mosunetuzumab (CD20xCD3 bispecific T-cell engager antibody), and chimeric antigen receptor (CAR)-T therapies represent major advances in three or later lines R/R FL treatment, such issues, like cytokine release syndrome (CRS), immune effector cell-associated neurotoxicity syndrome (ICANS), and secondary T-cell malignancies of CAR-T therapies,^27^ still raise wide concerns. We reasoned that TQ-B3525, as a PI3K inhibitor with a different mechanism of action from those three drugs, may serve as a valuable treatment addition and feasible therapeutic option for R/R FL patients, particularly for those in China. Besides, the oral modality of TQ-B3525 may also create convenience and accessibility for cancer therapy compared with CAR-T therapies.^28–31^ In this phase II registration study, we assessed the clinical outcomes of TQ-B3525 in R/R FL following two or more lines of previous therapies.

## Results

### Patient characteristics

Between May 20, 2020, and November 26, 2020, 25 patients were enrolled at the run-in stage; median age was 55.0 (range, 49–61) years and other baseline characteristics were found in Table S2.

**Fig. 1:**
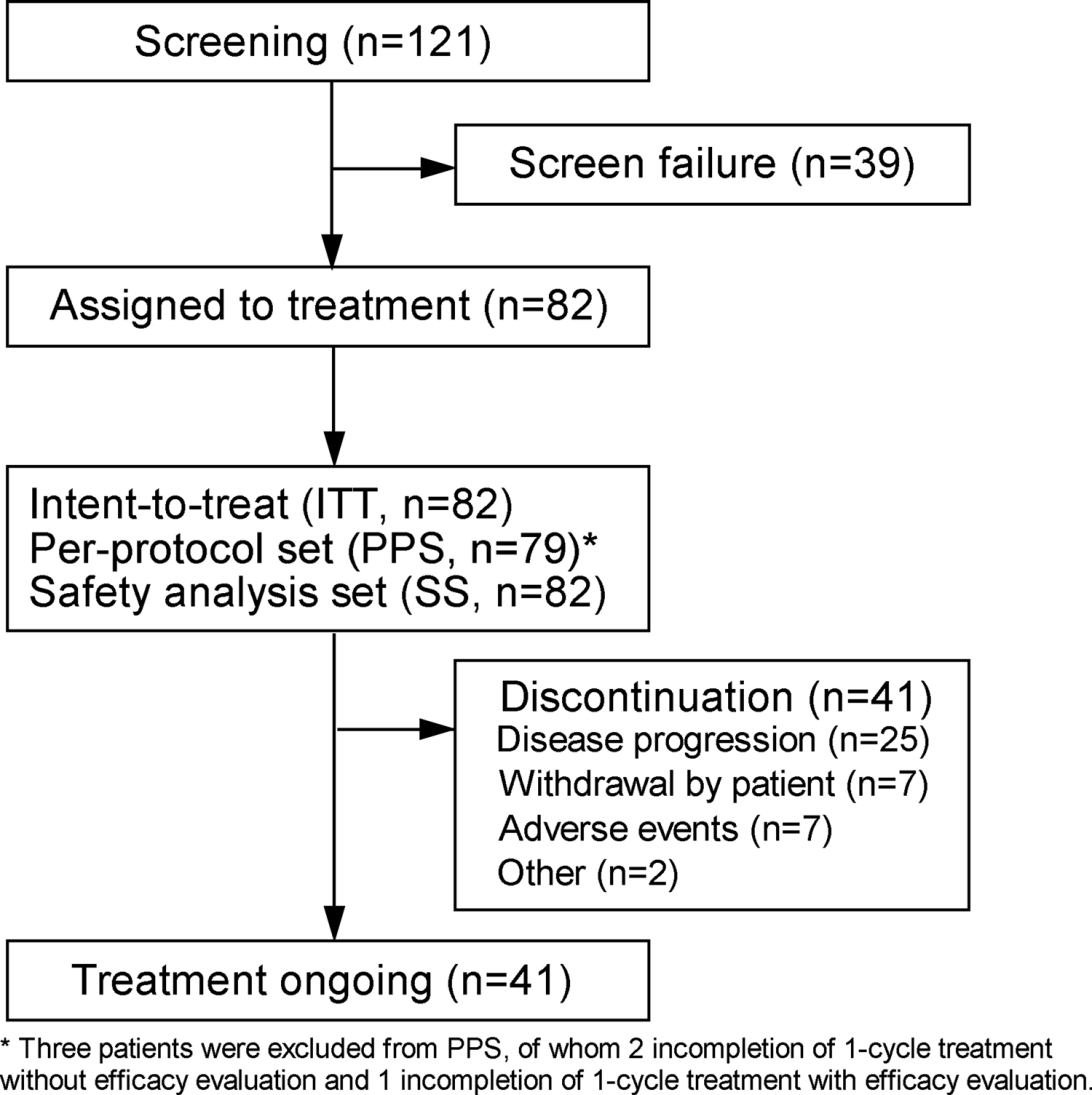
Trial profile at stage 2. SS safety analysis set, PPS per-protocol set, ITT intent-to-treat

Based on the results from the run-in stage, 121 patients from 41 sites in China were screened for stage 2 study from November 29, 2020, to June 18, 2022, of whom 82 were enrolled and included in the ITT and SS. As of the data cutoff (December 18, 2022), 41 (50.0%) patients were still on treatment; the majority (25/82, 30.5%) discontinued treatment due to disease progression (Fig. 1). Table 1 showed the baseline characteristics of 82 ITT populations. Median age was 52.5 (range, 32–76) years and 62.2% were male. Most patients (79/82, 96.3%) had ECOG PS of 0–1. The majority (64/82, 78.0%) had stage III-IV diseases, and median disease course of FL was 37.7 (range, 4.8-156.2) months. Forty-four (54.3%) were intermediate-/high-risk FL per Follicular Lymphoma International Prognostic Index 2 (FLIPI-2). All cases had received prior rituximab-based regimens, with R-CHOP/R-CDOP (76/82, 92.7%) being common; and 46 (56.1%) patients were refractory to last therapies. Median number of previous treatment lines was 3 (range, 2–7). No one received autologous/allogeneic stem cell transplants before the enrollment. Progression of disease within 24 months (POD24) at enrollment occurred in over half (73.2%).

**Table 1:** Baseline characteristics at stage 2

Characteristics	ITT population (*n* = 82)
Sex	
Male	51 (62.2%)
Female	31 (37.8%)
Age, years-median (range)	52.5 (32–76)
<60	56 (68.3%)
≥60	26 (31.7%)
ECOG PS	
0	43 (52.4%)
1	36 (43.9%)
2	3 (3.7%)
Median time from initial diagnosis to start of study treatment, months (range)	37.7 (4.8-156.2)
Histological grade	
Grade 1–2	69 (84.1%)
Grade 3a	13 (15.9%)
Relapsed to last therapies	36 (43.9%)
Refractory to last therapies	46 (56.1%)
Primary refractory	16 (19.5%)
Prior therapy to which the disease was refractory	
Rituximab	40 (48.8%)
Rituximab and alkylating agents	26 (31.7%)
FLIPI-2^a^	
0–1	37 (45.7%)
2	23 (28.4%)
3–5	21 (25.9%)
Lugano stage	
I–II	9 (11.0%)
III-IV	64 (78.0%)
Other	9 (11.0%)
Bone marrow involvement	
Yes	9 (11.0%)
No	71 (86.6%)
Not estimable	2 (2.4%)
Prior radiotherapy	
Yes	12 (14.6%)
No	70 (85.4%)
Organ or stem cell transplant (autologous/allogeneic)	
No	82 (100.0%)
POD24	
Yes	60 (73.2%)
No	22 (26.8%)
Lines of prior systemic therapies, median (range)	3.0 (2.0, 7.0)
2	38 (46.3%)
3	26 (31.7%)
>3	18 (22.0%)
Prior therapies	
Rituximab-based regimens	82 (100.0%)
R-CHOP/R-CDOP	76 (92.7%)
Rituximab plus bendamustine	19 (23.2%)
Rituximab plus lenalidomide	1 (1.2%)
Alkylating agents	82 (100.0%)
Immunomodulatory drugs	45 (54.9%)
Bendamustine	26 (31.7%)
BTK inhibitors	13 (15.9%)

Data were presented as the median (range) or n (%)

*ITT* intent-to-treat, *POD24* progression of disease within 24 months, *BTK* Bruton tyrosine kinase, *FLIPI-2* Follicular Lymphoma International Prognostic Index 2, *ECOG PS* Eastern Cooperative Oncology Group Performance Score

^a^Totally 81 patients had FLIPI-2 score because case 49,001 did not complete the initial treatment examination and thus did not evaluate the FLIPI-2

### Efficacy

Median follow-up was 24.9 months at the run-in stage (cutoff date, December 18, 2022). Among 25 patients with evaluable efficacy, 6 (24.0%) patients achieved CR based on the IRC assessment (Table S3). The percentage of patients with the IRC-assessed objective response and disease control was 88.0% (95% CI, 68.8–97.5%) and 92.0% (95% CI, 74.0–99.0%), respectively. Median DOR was 11.8 (95% CI, 5.5-not estimable) months. Median PFS was 12.0 (95% CI, 7.3-not estimable) months; 24-month OS rate was estimated as 78.9% (95% CI, 56.4–90.6%) and median OS was not reached. The consistent results (ORR, 88.0%; CRR, 20.0%; DCR, 96.0%; median DOR, 14.8 months; median PFS, 10.9 months) assessed by the investigator were provided in Table S3.

**Fig. 2:**
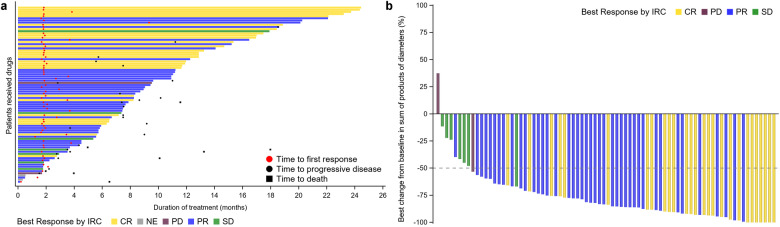
TQ-B3525 treatment and response outcomes in the ITT at stage 2. **a** Swimmer plot. **b** Waterfall plot of tumor change from baseline. PD progressive disease, SD stable disease, IRC independent review committee, PR partial response, NE not estimable, CR complete response

Median follow-up at the stage 2 was 13.3 (95% CI, 10.1–18.9) months by the data cutoff (December 18, 2022). Of 82 patients, 28 (34.2%) achieved CR and 43 (52.4%) achieved PR, with IRC-assessed ORR of 86.6% (95% CI, 77.3–93.1%; Table S4). The study met its predefined primary endpoint, which significantly rejected the null hypothesis of ORR (≤40%). SD was attained in 7 (8.5%) patients and PD in 2 (2.4%) patients (Fig. 2a). As per IRC assessment in 82 patients, 70 (85.4%) achieved lesion reduction of at least 50% following TQ-B3525 treatment (Fig. 2b). Consistent with the IRC assessment, the investigator assessment showed an ORR of 87.8% (95% CI, 78.7–94.0%; Table S4). Also, ORR (IRC-assessed 88.6%; investigator-assessed, 89.9%) in the PPS yielded similar results to the analysis in the ITT (Table S5).

**Fig. 3:**
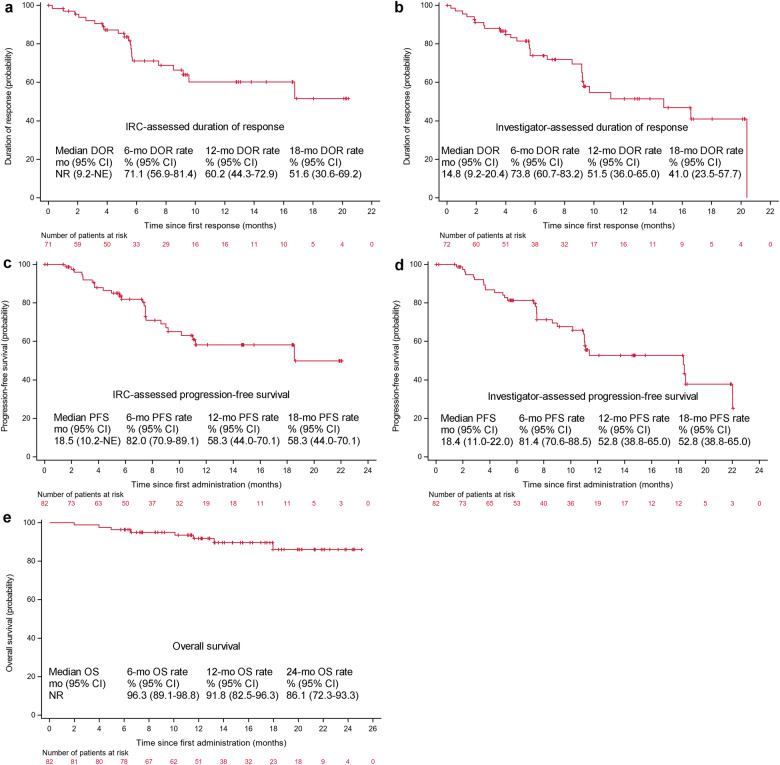
Kaplan-Meier curves of DOR, PFS, and OS at stage 2 (ITT population). DOR as per IRC (**a**) and investigator assessments (**b**). PFS as per IRC (**c**) and investigator assessments (**d**). OS (**e**). DOR duration of response, mo months, OS overall survival, NR not reached, NE not estimable, CI confidence interval, IRC independent review committee, PFS progression-free survival

Responses for stage 2 were rapid and durable. For the ITT population, IRC-assessed median TTR was 1.8 (range, 0.2–9.3; Table S4) months, and DOR was not reached (95% CI, 9.2-not estimable; Fig. 3a). Investigator-assessed median TTR (1.8 months) and DOR (14.8 months) were similar to the above results (Table S4 and Fig. 3b). There were 26 (31.7%) PFS events as assessed by IRC; median PFS was 18.5 (95% CI, 10.2-not estimable; Fig. 3c) months, with both 58.3% of the patients remaining progression-free at 12 and 18 months. Investigator assessment also demonstrated similar PFS results (18.4 months; Fig. 3d). Median OS was not reached because of insufficient events (8 [9.8%] deaths; Fig. 3e); 12- and 24-month OS rates were respectively estimated as 91.8% (95% CI, 82.5–96.3%) and 86.1% (95% CI, 72.3–93.3%). A PPS analysis with DOR (IRC-assessed, not reached; investigator-assessed, 14.8 months), PFS (IRC-assessed, 18.5 months; investigator-assessed, 18.4 months), and OS (not reached) was consistent with these results in the ITT (Table S5 and Fig. S1).

**Fig. 4:**
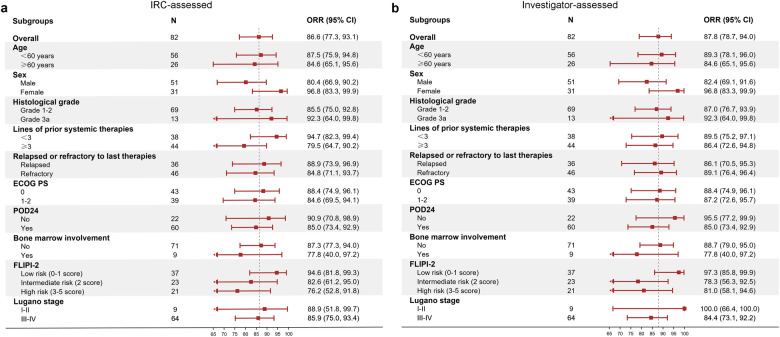
Forest Plot of subgroup analysis for ORR in ITT at stage 2. Response rates were assessed by IRC (**a**) and investigator (**b**). ORR objective response rate, ITT intent-to-treatment, CI confidence interval, POD24 progression of disease within 24 months, IRC independent review committee, ECOG PS Eastern Cooperative Oncology Group Performance Score, FLIPI-2 Follicular Lymphoma International Prognostic Index 2

Subgroup analysis at stage 2 showed that response rates confirmed by IRC (ORR range, 76.2–96.8%; Fig. 4a) and investigator (ORR range, 77.8–-100.0%; Fig. 4b) in the ITT population were consistent across all patient subgroups including age, sex, histological grade, lines of prior systemic therapies, relapsed or refractory to last therapies, ECOG PS, POD24, bone marrow involvement, FLIPI, and Lugano stage. Favorable DOR, PFS, and OS were also observed in various subgroups (Fig. S2). Analysis in subpopulation with high-aggressive disease indicated by POD24 revealed that ORR assessed by IRC (90.9% vs. 85.0%) and investigator (95.5% vs. 85.0%) was numerically higher in non-POD24 than POD24 patients. Of note, patients who are clinically challenging to treat, such as those with POD24 (90.9%), refractory diseases to last therapies (84.8%), or Lugano stage III-IV (85.9%), exhibited a high IRC-assessed ORR exceeding 80.0%. Patients who underwent third-line or later treatments also have an ORR of 79.5% by IRC assessment. Patients with primary refractory disease attained an IRC-assessed ORR of 87.5%; besides, median DOR and PFS assessed by IRC and median OS were not reached.

### Safety

Median exposure with TQ-B3525 was 7.5 (range, 0.1–23.5) months and median exposure was 8.5 (range, 0–26) cycles (Table S6). Median actual and relative dose intensity (RDI), along with cumulative dose of TQ-B3525 were 480.7 (range, 310.6–615.4) mg/day, 79.0% (range, 51.0–100.0%), and 4050.0 (range, 80.0–14300.0) mg, respectively. Nearly half (46.3%) received 80–120% of RDI.

Any-grade treatment-related AEs (TRAEs) were observed in all 25 (100.0%) patients at the run-in stage, and mainly included hyperglycemia (19/25, 76.0%), neutropenia (16/25, 64.0%), diarrhea (15/25, 60.0%). Grade 3 or higher TRAEs were reported in 19 (76.0%) cases and primarily were neutropenia (9/25, 36.0%) and hyperglycemia (7/25, 28.0%). There were no deaths from any cause at the run-in stage (Table S7).

TRAE with an incidence of ≥10% at stage 2 was summarized in Table 2. All 82 patients in the SS experienced at least one treatment-related AE (TRAE) of any grade; frequent TRAEs were hyperglycemia (68/82, 82.9%), diarrhea (46/82, 56.1%), neutropenia (37/82, 45.1%), and leukopenia (31/82, 37.8%). Grade 3 or higher TRAEs occurred in 63 (76.8%) cases, with neutropenia (18/82, 22.0%), hyperglycemia (16/82, 19.5%), and diarrhea (11/82, 13.4%) being common. The grade 3 or higher TRAEs of special interest occurring in 61.0% of patients mainly included neutropenia (18/82, 22.0%), hyperglycemia (16/82, 19.5%), diarrhea (11/82, 13.4%), thrombopenia (7/82, 8.5%), lymphopenia (7/82, 8.5%), infectious pneumonia (6/82, 7.3%), and pneumonitis (5/82, 6.1%; Table S8). Serious TRAEs occurred in 39 (47.6%) patients and mainly included infectious pneumonia (8.5%), pneumonitis (7.3%), hyperglycemia (6.1%), diarrhea (6.1%), interstitial lung disease (3.7%), and upper respiratory infection (3.7%). Dosing of TQ-B3525 was reduced in 61 (74.4%) and interrupted in 60 (73.2%) patients due to TRAEs (Table S9). Eight (9.8%) patients discontinued TQ-B3525 as a result of a TRAE. Among 13 patients with pneumonitis, 3 dose reductions, 7 dose interruptions, and 2 treatment discontinuations were observed.

**Table 2:** Treatment-related adverse events (TRAEs) with incidence of ≥10% in SS at stage 2

	Safety population (*n* = 82)	
	Any grade	Grade 3 or more
TRAEs	82 (100.0%)	63 (76.8%)
Hyperglycemia	68 (82.9%)	16 (19.5%)
Diarrhea	46 (56.1%)	11 (13.4%)
Neutropenia	37 (45.1%)	18 (22.0%)
Leukopenia	31 (37.8%)	9 (11.0%)
Weight loss	30 (36.6%)	2 (2.4%)
Thrombopenia	30 (36.6%)	7 (8.5%)
Lymphopenia	26 (31.7%)	7 (8.5%)
Anemia	24 (29.3%)	3 (3.7%)
Hypokalemia	23 (28.1%)	6 (7.3%)
Blood bilirubin increased	18 (22.0%)	0
Decreased appetite	16 (19.5%)	3 (3.7%)
Glycosylated hemoglobin (A1c) elevated	16 (19.5%)	2 (2.4%)
Proteinuria	15 (18.3%)	0
Alanine aminotransferase increased	15 (18.3%)	2 (2.4%)
Hypoalbuminemia	14 (17.1%)	0
Glucosuria	14 (17.1%)	1 (1.2%)
Pneumonitis	13 (15.9%)	5 (6.1%)
Nausea	13 (15.9%)	0
Vomiting	12 (14.6%)	1 (1.2%)
Fatigue	12 (14.6%)	3 (3.7%)
Infectious pneumonia	12 (14.6%)	6 (7.3%)
Increased C-reactive protein	12 (14.6%)	3 (3.7%)
Aspartate aminotransferase increased	12 (14.6%)	2 (2.4%)
Lipase increased	12 (14.6%)	4 (4.9%)
Positive urinary ketone body	11 (13.4%)	0
Creatinine increased	11 (13.4%)	0
Blood lactate dehydrogenase increased	11 (13.4%)	0
CD4 lymphocytes decreased	9 (11.0%)	3 (3.7%)
Elevated conjugated bilirubin	9 (11.0%)	0
Hyponatremia	9 (11.0%)	0

Data were presented as n (%)

*TRAEs* treatment-related adverse events

Only 1 death occurred and was considered possibly TQ-B3525 treatment related: a 56-year-old male with baseline ruptured neck mass experienced sepsis after approximately 1.5 months of first treatment and finally died following the discontinuation of TQ-B3525 and symptomatic treatment (Table S10).

All 82 patients experienced treatment-emergent AEs (TEAEs) at stage 2, of which 81.7% had grade 3 or higher events (Table S11). Commonly reported grade 3 or higher TEAEs included neutropenia (18/82, 22.0%), hyperglycemia (16/82, 19.5%), and diarrhea (13/82, 15.9%). Serious TEAEs occurred in 46 (56.1%) patients.

## Discussion

This phase II study met the primary endpoint with statistical significance, demonstrating an ORR of 86.6% (CRR, 34.2%). A median TTR of 1.8 months and an 18-month DOR rate of 51.6% suggested rapid and durable responses. Median PFS and OS were respectively 18.5 months and not reached. Favorable response and survival were consistent across patient subgroups, which was in favor of TQ-B3525 treatment for R/R FL regardless of baseline characteristics.

Currently, several PI3K inhibitors, including idelalisib, copanlisib, duvelisib, and linperlisib, reported an ORR of 42–79.8% and CRR ranging from 1 to 15.5% in patients with R/R FL.^12–14,32^ In contrast, the efficacy data for TQ-B3525 suggested a superior response rate (86.6%) with particularly a higher CRR of 34.2%. Also, median DOR (IRC-assessed not reached, investigator-assessed 14.8 vs. 10.8–12.3 months) and PFS (18.5 vs. 9.5–13.4 months) with TQ-B3525 were also longer than these agents. Despite limitations of cross-trial comparisons, TQ-B3525 monotherapy elicited significant efficacy.

Compared to pivotal trials with PI3K inhibitors in indolent lymphoma,^12–14^ high and durable TQ-B3525 responses may be partly due to the histologic subtype with all FL in our study; as demonstrated in the copanlisib trial, the frequent and dominant up-regulation of the BCR/PI3K signaling in FL may result in FL patients being more likely to respond to treatment.^16^ Usually, inhibition of PI3K-δ led to compensatory activation of PI3K-α, undermining the intended therapeutic effect^33^; consequently, it was speculated that the better activity of TQ-B3525 compared to PI3K-δ or PI3K-α inhibitors may be due to simultaneous blockade of PI3K-α and PI3K-δ and overcoming subsequent resistance. However, further explorations are warranted to elucidate the mechanism for antitumor effects of TQ-B3525 in lymphoma, including the collection of tumor samples from patients developing resistance to PI3Kδ inhibitors to assess the activation status of the PI3Kα and the response to TQ-B3525 and the evaluation of PI3Kδ inhibitors and TQ-B3525 in lymphoma animal models with PI3Kα constitutive activation.

The baseline characteristics in TQ-B3525 study were comparable to R/R FL with approved PI3K inhibitors in pivotal clinical trials. Thereinto, the majority were diagnosed with advanced-stage disease (78.0%) and had ECOG PS of 0–1 (96.3%) and histologically confirmed low-grade malignancy (84.1%) at enrollment. Thrillingly, the subpopulation with Lugano stage III-IV attained numerically superior ORR (85.9% vs. 42–79.8%) in our study compared with the entire population in copanlisib, linperlisib, idelalisib, and duvelisib trials.^12–14,32^ As evidenced by extensive literature reporting FLIPI-2 as a prognostic tool for FL,^10,34^ the fact that most enrolled patients classified as low- intermediate risk by the FLIPI-2 may be one plausible explanation for favorable response and survival, consistent with the findings from subgroup stratified by FLIPI-2 (low vs. intermediate vs. high risk: ORR, 94.6% vs. 82.6% vs. 76.2%). Besides, data from predefined subgroup analysis also indicated that rare bone marrow involvement (11.0%) representing a potential consideration conferred an overall benefit, which was typical for Chinese FL patients.^35^ Importantly, patients with bone marrow involvement (77.8%) and high FLIPI-2 scores (76.2%) also achieved a deep response of nearly 80%. Notably, compared to ones previously receiving first- or second-line therapies, patients experiencing prior third-line or later treatments had favorable improvements in DOR (HR, 0.55) and PFS (HR, 0.61); while cautious interpretation should be noted for the relation between the TQ-B3525 treatment sensitivity and pretreatment extent due to limited overall sample size. While the previous studies that breast cancer patients resistant to endocrine therapy exhibiting stronger PI3K inhibitor treatment sensitivity had frequent occurrence of PI3K mutations and overactivation of the PI3K/Akt/mTOR signaling^36^ suggested the need for future focus on the characterization of the molecular and genomic signatures to identify lymphoma patients who could better benefit from TQ-B3525.

In this study, over half (56.1%) had disease refractory to previous last therapies and all priorly received rituximab-based regimens and alkylating agents. Most patients (73.2%) progressed within 2 years (POD24) after first treatment regimen. It has been established that POD24 was identified as a convincing adverse prognostic factor for FL.^37,38^ Subgroup analyses showed an ORR of 85.0% for POD24 patients compared with 90.9% for non-POD24. Besides, better DOR (HR, 0.69) and PFS (HR, 0.68) were observed in patients with POD24 than non-POD24. Irrespective of the POD24 status, patients treated with TQ-B3525 had a numerically impressive response and survival than with linperlisib.^32^ Overall, by all of the stratified criteria combined, TQ-B3525 achieved a clinically meaningful therapeutic effect for R/R FL patients regardless of patient baseline characteristics, making it a promising treatment option in the evolving therapeutic landscape.

The safety of TQ-B3525 warranted intense scrutiny following the black box warnings for several PI3K inhibitors.^39,40^ Overall, the AE profiles observed here were consistent with those with single-agent TQ-B3525 from phase I study and other PI3K inhibitors^12,14,26,32,41–43^; no unexpected or new safety signals were found. The frequent hyperglycemia reported here, the known on-target effect of PI3Kα inhibition as mediating insulin signaling,^44^ was expectedly higher than several PI3Kδ inhibitors.^12,32,43^ However, hyperglycemia was mostly grade 1–2 and controllable using dose modification or supportive care. Notably, grade 3 or higher hyperglycemia (19.5%) was less frequent compared to copanlisib (41%).^13^ Besides, fewer hypertension events (any-grade, 4.9% vs. 30%; grade ≥3, 1.2% vs. 24%) were documented here over those in copanlisib trials.^13^ Considering the risk of hyperglycemia and hypertension potentially relating to the intravenous infusion mode of copanlisib,^45,46^ our oral dosing schedule continuously may also represent a feasible strategy instead of the intravenous dosing of copanlisib intermittently. Although gastrointestinal and liver toxicities and cutaneous reactions were commonly seen with the inhibition of PI3Kδ,^18,47^ alanine aminotransferase (18.3% vs. 23–47%) and aspartate aminotransferase increased (14.6% vs. 28%-35%) were presented here at a lower incidence compared with idelalisib and copanlisib^12,13^; and no colitis was reported. In addition, diarrhea occurred frequently (56.1%) and was generally self-limiting (median time of onset of the first episode, 15 [range, 5–83] days), which did not lead to related deaths. Similarly to prior studies,^12–14,32^ hematologic AEs were also frequent in our study; importantly in this study, no patients experienced severe myelosuppression and discontinuation of TQ-B3525 as a result of hematologic toxicities. Of note, pneumonitis occurred in 15.9% of patients, which highlighted the importance of close monitoring and early intervention of respiratory symptoms in the prescribing information for TQ-B3525. Among 301 patients from all TQ-B3525 studies, 58 patients experiencing TRAEs with pneumonia-like symptoms, of whom 37.9% received glucocorticoids. Only 1 death occurred due to sepsis. TQ-B3525 was not recommended for diabetic patients. Of note, personalized dosing strategy with proactive dose modifications when patients achieved CR or experienced certain grade 1–2 toxicities specified in our protocol may partly contribute to favorable safety of TQ-B3525 monotherapy in treatment with R/R FL patients.

Taken together, TQ-B3525, an oral dual PI3Kα/δ inhibitor, demonstrated a deep and durable response in clinically challenging FL patients. In terms of safety, TQ-B3525 reduced toxicities with hyperglycemia and hypertension compared with intravenous copanlisib and also presented differentiated AE profiles relative to PI3Kδ inhibitors. Besides, no life-threatening hepatitis, colitis, intestinal perforation, or skin reactions were reported in TQ-B3525 compared to PI3Kδ inhibitors. Given the limited third- or later-line therapies, TQ-B3525 may represent a novel potential and attractive therapeutic approach for R/R FL. However, the safety results comparing TQ-B3525 with other PI3K inhibitors should be interpreted with caution considering the differences in chemical structure, dosing mode, and ethnicity.^12,17,45,46^ Overall, larger-scale randomized trials and long-term safety follow-up are required to ascertain safety profiles of TQ-B3525; besides, conducting Meta-analysis of existing studies on PI3K inhibitors and real-world studies could enhance our understanding of the effects of TQ-B3525. In parallel, more future efforts ensuring the clinical safety application of TQ-B3525 should also focus on appropriate intermittent dosing regimens and effective toxicity management (such as CD4 monitoring, prophylactic antibiotics [Abx], intravenous immunoglobulin [IVIG] administration, and optimization of the timing, dosage, and duration of steroids).

Of note, a randomized phase III trial in relapsed indolent non-Hodgkin lymphoma demonstrated that the combination of copanlisib and rituximab significantly improved survival compared to placebo plus rituximab^48^; it suggested that PI3K inhibitors could combined with drugs frequently used in the FL treatment (e.g. lenalidomide and rituximab) to potentially synergistically enhance the antitumor efficacy. Furthermore, beyond the impressive antitumor activity of TQ-B3525 monotherapy, several preclinical investigations and clinical studies have guided future efforts in translational and clinical research towards combining TQ-B3525 with EZH2 inhibitors, bispecific antibodies, CAR-T therapy, BTK inhibitors, and PD-1 monoclonal antibodies in dual or triple therapies.^49–52^

Several limitations of our study should be noted. First, single-arm design without an external control group posed a challenge to accurately interpret cross-trial comparisons and provide a compelling evaluation of the impact of TQ-B3525. Second, extended survival and safety follow-up will be necessary to further assess clinical impacts of TQ-B3525. Finally, all patients were Chinese, potentially limiting the generalizability of our findings to other racial/ethnic groups.

In conclusion, oral TQ-B3525 exhibited favorable efficacy and manageable safety profiles, positioning it as a novel valuable therapeutic modality for heavily pretreated Chinese R/R/ FL patients. More thrillingly, TQ-B3525 received breakthrough therapy designation in July 2021 for the second or later-line R/R/ FL treatments.

## Materials and methods

### Study design

This open-label, single-arm, phase II study (ClinicalTrial.gov Identifier: NCT04324879) comprised a run-in stage followed by a stage 2 and was conducted at 41 sites in China. It primarily evaluated the clinical outcomes of TQ-B3525 in R/R FL patients after ≥2 prior systemic therapies. The study protocol was approved by ethics committees of primary centers with Sun Yat-sen University Cancer Center (NO. A2020-001-03) and Tianjin Union Medical Center (NO. 2021 [06]) and ethics committees of other centers. All patients have signed informed consent. This study was in compliance with the international standards of good clinical practice, the Declaration of Helsinki, and local laws and regulations.

### Patient population

Patients aged at least 18 years with histopathologically proven grade 1-3a FL and at least one radiologically measurable malignant lesion of lymph nodes as well as extranodal lymphoma were eligible. Patients experienced relapsed and/or refractory diseases after ≥2 lines of therapies, including rituximab or anti-CD20 monoclonal antibody. Refractoriness was defined per protocol as being unresponsive to at least one-line rituximab-containing therapy or progressing during the therapy or 6 months following treatment completion. Patients with life expectancy of ≥3 months, adequate organ functions, and Eastern Cooperative Oncology Group performance status (EGOG PS) ≤ 2 were enrolled.

Patients with active central nervous system lymphoma, diabetes, transformation of FL to diffuse large B-cell lymphoma, previous treatments with PI3K inhibitors or CAR-T, or autologous stem-cell transplant within 3 months before first TQ-B3525 dose or previous allogeneic (at any time) or autologous (within 3 months before the first dosing) hematologic stem-cell transplant were excluded. Complete eligibility criteria were detailed in Table S12.

### Procedures

Patients were administered orally 20 mg TQ-B3525 (Chia Tai Tianqing Pharmaceutical Group Co., Ltd., Nanjing, China) once daily in 28-day cycles unless unacceptable toxicity occurred or disease progressed. Patients attaining complete response (CR) were required dose reductions of TQ-B3525 to 15 or 10 mg daily, with later doses at the investigator’s discretions. Patients were permitted a maximum of two dose reductions when clinically significant toxicities occurred; re-escalation was not allowed. Once cumulative duration of dosing interruption due to toxicities exceeded 4 weeks, treatment was discontinued. Detailed dose titration criteria were described in Table S13.

### Endpoints and assessments

The primary endpoint was independent review committee (IRC)-assessed ORR, including patients achieving a CR and partial response (PR) as per the 2014 Lugano classification response criteria.^53^ Case experiencing bone marrow involvement at baseline were required evaluation of bone marrow biopsies to confirm CR. Secondary endpoints were ORR by investigator assessment; the IRC- and investigator-assessed disease control rate (DCR, percent of a CR, PR, and stable disease [SD]), duration of response (DOR, time from the first response to progressive disease [PD] or death, whichever occurred first), time to response (TTR, interval between starting TQ-B3525 and achieving CR or PR), progression-free survival (PFS, time between treatment initiation and PD or any-cause death, whichever came first), overall survival (OS, time between first dose of TQ-B3525 and all-cause death), and safety. Adverse events (AEs) were graded based on the National Cancer Institute Common Terminology Criteria for Adverse Events (version 5.0).^54^

Tumors were assessed by positron emission tomography-computed tomography (PET-CT) and computed tomography (CT)/magnetic resonance imaging (MRI) per 2014 Lugano classification response criteria during the 28-day screening. CT/MRI and PET-CT for response assessments were respectively carried out at 8-week and 16-week intervals at the first 48 weeks of treatment, and 16-week and 32-week intervals thereafter. PET-CT was required to further confirm CR assessed using CT/MRI within 7 days, followed by being waived within the subsequent 8 weeks.

### Statistical analysis

There was no formal estimation of sample size due to the exploratory design of the run-in stage study. The stage 2 registration study was carried out based on the data from the run-in stage. Sample size at the stage 2 was determined by the primary endpoint ORR. A sample of 64 patients had a power of 90% to test the hypothesis that the ORR would be 60% against the null hypothesis that it would be 40% or lower, with 0.025 as one-sided significance level. Totally 80 subjects were required, considering the dropout incidence of 20%.

The efficacy was primarily analyzed in intent-to-treat (ITT) population, consisting of all enrolled subjects irrespective of using the study drug. Efficacy data in the per-protocol set (PPS), which comprised the ITT population who had no major protocol violations, exhibited good compliance, and completed at least one-cycle therapy and one post-baseline tumor assessment, was analyzed as supportive results. Safety was evaluated in safety analysis set (SS), including cases with at least one dosing and safety records.

Patient characteristics and safety analyses were summarized descriptively. Descriptive measures for continuous variables were interquartile range (IQR) or range, and for categorical variables were frequencies (percentage [%]). The 95% confidence intervals (CI) of DCR and ORR were assessed by binomial Clopper-Pearson method. The correlation between response rates and clinicopathological factors was evaluated with chi-square test. OS, PFS, DOR, and TTR were analyzed by Kaplan-Meier method and between-group differences were compared by the log-rank test. Survival data from subjects without disease progression or death were censored at the last tumor assessment. A two-sided *p* value < 0.05 represents statistically significant in all analyses by SAS software (v.9.4; SAS Institute, Cary, NC, USA).

### Supplementary information

TTQ-B3525-II-01 protocol

Sigtrans_Supplementary_Materials

## Data Availability

The datasets in this study are available upon request from the corresponding author.

## References

[1] Carbone A (2019). Follicular lymphoma. Nat. Rev. Dis. Primers.

[2] Casulo C (2015). Early relapse of follicular lymphoma after rituximab plus cyclophosphamide, doxorubicin, vincristine, and prednisone defines patients at high risk for death: an analysis from the national LymphoCare study. J. Clin. Oncol..

[3] Casulo C, Barr PM (2019). How I treat early-relapsing follicular lymphoma. Blood.

[4] Hanel W, Epperla N (2021). Evolving therapeutic landscape in follicular lymphoma: a look at emerging and investigational therapies. J. Hematol Oncol..

[5] Denlinger N, Bond D, Jaglowski S (2022). CAR T-cell therapy for B-cell lymphoma. Curr. Probl. Cancer.

[6] Gordon MJ, Smith MR, Nastoupil LJ (2023). Follicular lymphoma: The long and winding road leading to your cure?. Blood Rev..

[7] Rivas-Delgado A (2019). Response duration and survival shorten after each relapse in patients with follicular lymphoma treated in the rituximab era. Br. J. Haematol.

[8] Taverna C (2016). Rituximab maintenance for a maximum of 5 years after single-agent rituximab induction in follicular lymphoma: Results of the randomized controlled phase III trial SAKK 35/03. J. Clin. Oncol.

[9] Fischer L, Dreyling M (2023). Follicular lymphoma: an update on biology and optimal therapy. Leuk Lymphoma.

[10] Jacobsen E (2022). Follicular lymphoma: 2023 update on diagnosis and management. Am. J. Hematol.

[11] De Santis MC, Gulluni F, Campa CC, Martini M, Hirsch E (2019). Targeting PI3K signaling in cancer: Challenges and advances. Biochim. Biophys. Acta Rev. Cancer.

[12] Gopal AK (2014). PI3Kδ inhibition by idelalisib in patients with relapsed indolent lymphoma. N. Engl. J. Med..

[13] Dreyling M (2017). Phosphatidylinositol 3-kinase inhibition by copanlisib in relapsed or refractory indolent lymphoma. J. Clin. Oncol..

[14] Flinn IW (2019). DYNAMO: a phase II Study of duvelisib (IPI-145) in patients with refractory indolent Non-Hodgkin lymphoma. J. Clin. Oncol.

[15] Fowler NH (2021). Umbralisib, a dual PI3Kδ/CK1ε inhibitor in patients with relapsed or refractory indolent lymphoma. J. Clin. Oncol..

[16] Liu L (2017). Tumor gene expression signatures of BCR/PI3K dependence in association with copanlisib monotherapy activity in heavily pretreated patients with indolent NHL and follicular lymphoma. Ann. Oncol..

[17] Shin N (2020). Parsaclisib is a next-generation phosphoinositide 3-kinase δ inhibitor with reduced hepatotoxicity and potent antitumor and immunomodulatory activities in models of B-cell malignancy. J. Pharmacol Exp. Ther..

[18] Yu M (2023). Development and safety of PI3K inhibitors in cancer. Arch. Toxicol.

[19] Iyengar S (2013). P110α-mediated constitutive PI3K signaling limits the efficacy of p110δ-selective inhibition in mantle cell lymphoma, particularly with multiple relapse. Blood.

[20] Paul J (2017). Simultaneous inhibition of PI3Kδ and PI3Kα induces ABC-DLBCL regression by blocking BCR-dependent and -independent activation of NF-κB and AKT. Cancer Cell.

[21] Vanhaesebroeck B, Perry MWD, Brown JR, André F, Okkenhaug K (2021). PI3K inhibitors are finally coming of age. Nat. Rev. Drug Discov..

[22] Serrat N (2020). PI3Kδ inhibition reshapes follicular lymphoma-immune microenvironment cross talk and unleashes the activity of venetoclax. Blood Adv..

[23] Lannutti BJ (2011). CAL-101, a p110delta selective phosphatidylinositol-3-kinase inhibitor for the treatment of B-cell malignancies, inhibits PI3K signaling and cellular viability. Blood.

[24] Winkler DG (2013). PI3K-δ and PI3K-γ inhibition by IPI-145 abrogates immune responses and suppresses activity in autoimmune and inflammatory disease models. Chem. Biol..

[25] Krause G, Hassenrück F, Hallek M (2018). Copanlisib for treatment of B-cell malignancies: the development of a PI3K inhibitor with considerable differences to idelalisib. Drug. Des. Devel Ther..

[26] Wang H (2020). Safety and efficacy of TQ-B3525, a novel and selective oral PI3K α/δ inhibitor, in Chinese patients with advanced malignancies: A phase I dose-escalation and expansion trial. J. Clin. Oncol..

[27] Yang C, Nguyen J, Yen Y (2023). Complete spectrum of adverse events associated with chimeric antigen receptor (CAR)-T cell therapies. J. Biomed. Sci..

[28] Jacobson CA (2022). Axicabtagene ciloleucel in relapsed or refractory indolent non-Hodgkin lymphoma (ZUMA-5): a single-arm, multicentre, phase 2 trial. Lancet Oncol.

[29] Fowler NH (2022). Tisagenlecleucel in adult relapsed or refractory follicular lymphoma: the phase 2 ELARA trial. Nat Med..

[30] Budde LE (2022). Safety and efficacy of mosunetuzumab, a bispecific antibody, in patients with relapsed or refractory follicular lymphoma: a single-arm, multicentre, phase 2 study. Lancet Oncol.

[31] Morschhauser F (2020). Tazemetostat for patients with relapsed or refractory follicular lymphoma: an open-label, single-arm, multicentre, phase 2 trial. Lancet Oncol.

[32] Wang T (2023). The Oral PI3Kδ inhibitor linperlisib for the treatment of relapsed and/or refractory follicular lymphoma: A phase II, single-arm, open-label clinical trial. Clin Cancer Res.

[33] Sorensen R (2015). Investigation of the mechanism of idelalisib resistance in the follicular lymphoma WSU-Fsccl cell line. Blood.

[34] Federico M (2009). Follicular lymphoma international prognostic index 2: a new prognostic index for follicular lymphoma developed by the international follicular lymphoma prognostic factor project. J. Clin. Oncol.

[35] Zha J (2021). Clinical features and outcomes of 1845 patients with follicular lymphoma: a real-world multicenter experience in China. J. Hematol Oncol.

[36] Presti D, Quaquarini E (2019). The PI3K/AKT/mTOR and CDK4/6 pathways in endocrine resistant HR+/HER2− metastatic breast cancer: biological mechanisms and new treatments. Cancers.

[37] Sortais C (2020). Progression of disease within 2 years (POD24) is a clinically relevant endpoint to identify high-risk follicular lymphoma patients in real life. Ann. Hematol.

[38] Leonard JP (2022). POD24 in follicular lymphoma: time to be “wise. Blood.

[39] *US Food and Drug Administration. COPIKTRA (duvelisib) highlights of prescribing information*,https://www.accessdata.fda.gov/drugsatfda_docs/label/2018/211155s000lbl.pdf (2018).

[40] *US Food and Drug Administration. ZYDELIG (idelalisib) highlights of prescribing information*, https://www.accessdata.fda.gov/drugsatfda_docs/label/2014/206545lbl.pdf (2014).

[41] Younes A (2015). An open-label phase II study of buparlisib (BKM120) in patients with relapsed and refractory diffuse large B-cell lymphoma (DLBCL), mantle cell lymphoma (MCL) and follicular lymphoma (FL). Blood.

[42] Juric D (2018). Phosphatidylinositol 3-kinase α-selective inhibition with alpelisib (BYL719) in PIK3CA-altered solid tumors: results from the first-in-human study. J. Clin. Oncol.

[43] Lynch RC (2021). Efficacy and safety of parsaclisib in patients with relapsed or refractory follicular lymphoma: primary analysis from a phase 2 study (CITADEL-203). Blood.

[44] Knight ZA (2006). A pharmacological map of the PI3-K family defines a role for p110alpha in insulin signaling. Cell.

[45] Dreyling M (2017). Phase II study of copanlisib, a PI3K inhibitor, in relapsed or refractory, indolent or aggressive lymphoma. Ann. Oncol.

[46] Wang J (2022). Efficacy and safety of copanlisib in relapsed/refractory B-cell non-Hodgkin lymphoma: A meta-analysis of prospective clinical trials. Front Immunol.

[47] Guarente V, Sportoletti P (2021). Lessons, challenges and future therapeutic opportunities for PI3K inhibition in CLL. Cancers.

[48] Matasar MJ (2021). Copanlisib plus rituximab versus placebo plus rituximab in patients with relapsed indolent non-Hodgkin lymphoma (CHRONOS-3): a double-blind, randomised, placebo-controlled, phase 3 trial. Lancet Oncol.

[49] Mishra VS, Kumar N, Raza M, Sehrawat S (2020). Amalgamation of PI3K and EZH2 blockade synergistically regulates invasion and angiogenesis: combination therapy for glioblastoma multiforme. Oncotarget.

[50] Chandrasekaran, S. et al. Strategies to overcome failures in T-cell immunotherapies by targeting PI3K-δ and –γ. *Front Immunol*. **12**, 718621 (2021). 10.3389/fimmu.2021.718621 PMC8427697 34512641

[51] Stewart CM (2022). Phase I/Ib study of the efficacy and safety of buparlisib and ibrutinib therapy in MCL, FL, and DLBCL with serial cell-free DNA monitoring. Clin Cancer Res.

[52] Bennani NN (2019). Copanlisib in combination with nivolumab in subjects with relapsed/refractory diffuse large B-cell lymphoma and primary mediastinal large B-cell lymphoma: a phase 2 study. Blood.

[53] Cheson BD (2014). Recommendations for initial evaluation, staging, and response assessment of Hodgkin and non-Hodgkin lymphoma: the Lugano classification. J. Clin. Oncol.

[54] Freites-Martinez A, Santana N, Arias-Santiago S, Viera A (2021). Using the common terminology criteria for adverse events (CTCAE - Version 5.0) to evaluate the severity of adverse events of anticancer therapies. Actas Dermosifiliogr (Engl Ed).

