# Supplementary material for: Phase II study of novel orally PI3Kα/δ inhibitor TQ-B3525 in relapsed and/or refractory follicular lymphoma
Source: Signal Transduct Target Ther. 2024 Apr 17;9:99. doi: 10.1038/s41392-024-01798-0 (PMC11021411; doi:10.1038/s41392-024-01798-0)
Supplement: Supplementary file 1 — TTQ-B3525-II-01 protocol [file 41392_2024_1798_MOESM1_ESM.docx]

Registration classification: Class I chemical drug clinical approval letter No.: 2017L04051/3

TQ-B3525 in relapsed/refractory follicular lymphoma (FL)

Single-arm, multicenter phase II clinical trial

| Study No. | TQ-B3525-II-01 |
| --- | --- |
| Version No. | 3.0 |
| Version Date | 25 January 2021 |
| Sponsor | Chia Tai Tianqing Pharmaceutical Group Co., Ltd. |
| Study site | Tianjin Union Medical Center of Nankai University  Sun Yat-sen University Cancer Center |
| Clinical Study Director | Professor Huaqing Wang, Professor Zhiming Li |

Confidential information

| The information contained in this clinical trial protocol is confidential and proprietary to CHIA TAI TIANQING Pharmaceutical Group CO., LTD. It shall not be disclosed to any third party (group or individual) without prior written permission. If you hold this protocol without prior authorization, please contact CHIA TAI TIANQING Pharmaceutical Group CO., LTD in a timely manner and return the protocol and its copy to the company. |
| --- |

Organization Information Page

This study shall be jointly undertaken by the sponsor of clinical trial, clinical trial institution and clinical trial statistical analysis institution.

Sponsor of clinical trial: Chia Tai Tianqing Pharmaceutical Group Co., Ltd.

Contact address: No. 1099, Fuying Road, Jiangning District, Nanjing, Jiangsu Province

Function:

Initiate and apply for the study, provide the investigator's brochure, investigational drugs, study fund, organize, monitor, audit and assist in the development of the clinical trial protocol.

Main participants are as follows:

| Position | Name | Telephone | E-mail |
| --- | --- | --- | --- |
| Project Manager | Li Chen | 15989187311 | chenlicttq@163.com |
| Medical Manager | Fan Feng | 13260882293 | fengfan2014cpu@163.com |

Study site:

Tianjin Union Medical Center of Nankai University

Contact address: No. 190, Jieyuan Road, Hongqiao District, Tianjin

Functions: Recruitment of subjects, development of clinical trial protocol, organization and implementation of clinical trial, and acceptance of inspection by regulatory authorities at any time

Principal Investigator: Huaqing Wang

Tel.: 022-87729595

Sun Yat-sen University Cancer Center

Contact address: No. 651 Dongfeng Road, Yuexiu District, Guangzhou

Functions: Recruitment of subjects, development of clinical trial protocol, organization and implementation of clinical trial, and acceptance of inspection by regulatory authorities at any time

Principal investigator: Zhiming Li

Tel.: 020-87343765

Clinical study data management unit: Chia Tai Tianqing Pharmaceutical Group Co., Ltd.

Contact address: No. 1099, Fuying Road, Jiangning District, Nanjing, Jiangsu Province

Function: Responsible for data management of clinical trial

Contact Person: Yadong Miu

Contact number: 18551674600

Department of Biostatistics, School of Public Health, Nanjing Medical University

Contact address: No. 101, Longmian Avenue, Jiangning District, Nanjing

Function: Responsible for statistical analysis of data from clinical trials

Contact Person: Yu Hao

Tel.: 025-86868444

Note: Unless there is an important contact change (such as the sponsor or the responsible unit of the clinical study), the contact change of the team during the study will not be revised to the protocol, and the participants will be informed of the update and filing of the team information.

Signature Consent Page

I will earnestly fulfill the responsibilities of the sponsor and participate in this clinical study according to the current GCP regulations in China. I have read and confirmed this protocol (Study No.: TQ-B3525-II-01, Version No.: 3.0, Version Date: January 25, 2021) and agree to perform the relevant responsibilities according to the Chinese law, Declaration of Helsinki, Chinese GCP and this study protocol.

Sponsor of clinical study: CHIA TAI TIANQING Pharmaceutical Group CO., LTD.

| Wang Xunqiang |  |  |
| --- | --- | --- |
| Technical Lead (Print) | Signature | Date of Signature (DD/MMM/YYYY) |

Signature Consent Page

I will earnestly fulfill the responsibilities of the sponsor and participate in this clinical study according to the current GCP regulations in China. I have read and confirmed this protocol (Study No.: TQ-B3525-II-01, Version No.: 3.0, Version Date: January 25, 2021) and agree to perform the relevant responsibilities according to the Chinese law, Declaration of Helsinki, Chinese GCP and this study protocol.

Sponsor of clinical study: CHIA TAI TIANQING Pharmaceutical Group CO., LTD.

| Miao Yadong |  |  |
| --- | --- | --- |
| Lead Data Manager (Print) | Signature | Date of Signature (DD/MMM/YYYY) |

Signature Consent Page

I have read and confirmed this protocol (Study No.: TQ-B3525-II-01, Version No.: 3.0, Version Date: January 25, 2021). I agree to perform the relevant responsibilities in accordance with the laws of China, Declaration of Helsinki, China GCP and this study protocol.

Sponsor: Tianjin People's Hospital

| Huaqing Wang |  |  |
| --- | --- | --- |
| Principal Investigator (Print) | Signature | Date of Signature (DD/MMM/YYYY) |

Signature Consent Page

I have read and confirmed this protocol (Study No.: TQ-B3525-II-01, Version No.: 3.0, Version Date: January 25, 2021). I agree to perform the relevant responsibilities in accordance with the laws of China, Declaration of Helsinki, China GCP and this study protocol.

Study site: Sun Yat-sen University Cancer Center

| Zhiming Li |  |  |
| --- | --- | --- |
| Principal Investigator (Print) | Signature | Date of Signature (DD/MMM/YYYY) |

Signature Consent Page

I have read and confirmed this protocol (Study No.: TQ-B3525-II-01, Version No.: 3.0, Version Date: January 25, 2021). I agree to perform the relevant responsibilities in accordance with the laws of China, Declaration of Helsinki, China GCP and this study protocol.

Study sites:

|  |  |  |
| --- | --- | --- |
| Principal Investigator (Print) | Signature | Date of Signature (DD/MMM/YYYY) |

Signature Consent Page

I have read and confirmed this protocol (Study No.: TQ-B3525-II-01, Version No.: 3.0, Version Date: January 25, 2021). I agree to perform the relevant responsibilities in accordance with the laws of China, Declaration of Helsinki, China GCP and this study protocol.

Clinical study statistical analysis unit: Department of Biostatistics, School of Public Health, Nanjing Medical University

| Yu Hao |  |  |
| --- | --- | --- |
| Responsible (Print) | Signature | Date of Signature (DD/MMM/YYYY) |

Protocol Update Log

| Version No. | Version Date | Reason for Modification Description and Summary of Modifications |
| --- | --- | --- |
| 1.0 | 12 December 2019 | Initial version |
| 2.0 | October 10, 2020 | Improve the inclusion and exclusion requirements  Update the time requirement for tumor tissue samples  Corrected time points for PET-CT assessments  Update SAE handling reporting requirements |
| 3.0 | 25 January 2021 | Improve the inclusion and exclusion criteria  Modify sample size design  Refine dose adjustment and laboratory tests  Updated adverse reaction handling recommendations |
|  |  |  |
|  |  |  |
|  |  |  |

LIST OF ABBREVIATIONS

| Abbreviation | Full name in Chinese | Abbreviation | Full name in Chinese |
| --- | --- | --- | --- |
| AE | Adverse events | IRC | Independent Review Committee |
| ALT | Alanine aminotransferase | LDi | Longest transverse diameter of the lesion |
| AKT | Protein kinase B | LDH | Lactate dehydrogenase |
| ANC | Absolute neutrophil count | LDL | Low density lipoprotein |
| AST | Aspartate aminotransferase | LPD | Longest vertical diameter |
| AUC | Area under the plasma concentration-time curve | LVEF | Left ventricular ejection fraction |
| BTK | Bruton's tyrosine kinase | MCL | Mantle cell lymphoma |
| BUN | Blood urea nitrogen | MedDRA | Medical Dictionary for Regulatory Activities |
| CD20 | B-lymphocyte antigen CD20 | MRD | Minimal residual disease |
| CLL | Chronic lymphocytic leukemia | MRI | Magnetic resonance imaging |
| Cmax | Maximum plasma concentration | MTOR | Mammalian target of rapamycin |
| CNS | Central nervous system | MTD | Maximum tolerated dose |
| CMV | Cytomegalovirus | NHL | Non-Hodgkin's lymphoma |
| CR | Complete response | NYHA | New York Heart Association |
| CT | Computed tomography | ORR | Objective response rate |
| CTCAE | Common Terminology Criteria for Adverse Events | OS | Overall survival |
| CYP3A | Cytochrome P450 family 3 subfamily A | PD | Disease progression |
| DLT | Dose limiting toxicity | PET | Positron emission tomography |
| DLBCL | Diffuse large B-cell lymphoma | PET-CT | Positron emission tomography-computed tomography |
| DOR | Duration of Response | PFS | Progression-free survival |
| ECG | Electrocardiogram | PI3K | Phosphatidylinositol-3 kinase |
| ECOG | Eastern Cooperative Oncology Group | PK | Kinetics |
| ECRF | Electronic Case Report Form | PJP | Pneumocystis jirovecii pneumonia |
| EOT | End of treatment | PK | Kinetics |
| FAS | Full Analysis Set | PLT | Platelets |
| FDG | Fluorodeoxyglucose | PPD | Vector product of longest transverse diameter and vertical diameter |
| FFPE | Formalin-fixed paraffin-embedded | PPS | Per Protocol Set |
| FL | Follicular lymphoma | PR | Partial response |
| FLIPI-2 | Follicular Lymphoma International Prognostic Index 2 | PTEN | Phosphatase and tensin homolog |
| GCP | Good Clinical Practice | QD | Once daily |
| HBsAg | Hepatitis B surface antigen | QTc | QT interval corrected for heart rate |
| HBV | Hepatitis B Virus | R-chemo | Rituximab + Chemotherapy |
| HCV | Hepatitis C Virus | R-CHOP | Rituximab C Virus herapy heart rate gnost vincristine, and prednisone |
| HDL | High density lipoprotein | R/R | Relapsed or refractory |
| HIV | Human Immunodeficiency Virus | SAE | Serious adverse event |
| HL | Hodgkin lymphoma | SCT | Stem cell transplant |
| HSCT | Hematopoietic stem cell transplantation | SD | Stable disease |
| IC50 | Half inhibitory concentration | SLL | Small lymphocytic lymphoma |
| ICF | Informed Consent Form | SPD | Sum of the product of the maximum perpendicular diameters of multiple lesions |
| ICH | International Conference on Harmonisation | SUSAR | Suspected unexpected serious adverse event |
| IHC | Immunohistochemistry | TTP | Time to progression |
| INHL | Indolent non-Hodgkin's lymphoma | ULN | Upper limit of normal |

Contents

[Organization Information Page 1](#_Toc146097460)

[Signature Consent Page 3](#_Toc146097461)

[Signature Consent Page 4](#_Toc146097462)

[Signature Consent Page 5](#_Toc146097463)

[Signature Consent Page 6](#_Toc146097464)

[Signature Consent Page 7](#_Toc146097465)

[Signature Consent Page 8](#_Toc146097466)

[Protocol Update Log 9](#_Toc146097467)

[Study Abstract 14](#_Toc146097468)

[1 Study background 21](#_Toc146097469)

[2 Study objectives and endpoints 26](#_Toc146097470)

[2.1 Study Objectives 26](#_Toc146097471)

[2.2 Study Endpoints 26](#_Toc146097472)

[3 Study Design 26](#_Toc146097473)

[3.1 Overall Design 26](#_Toc146097474)

[3.2 Rationale for selection of primary study endpoints 27](#_Toc146097475)

[3.3 Sample Size Design 27](#_Toc146097476)

[4 Study subjects 27](#_Toc146097477)

[4.1 Inclusion Criteria 28](#_Toc146097478)

[4.2 Exclusion criteria 29](#_Toc146097479)

[4.3 Screen Failure 31](#_Toc146097480)

[4.4 Subjects Numbering principle 31](#_Toc146097481)

[4.5 Withdrawal Criteria 31](#_Toc146097482)

[4.6 Post-exit treatment 32](#_Toc146097483)

[5 Study drug 32](#_Toc146097484)

[5.1 General Information of Study Drug 32](#_Toc146097485)

[5.2 Study Drug Management 33](#_Toc146097486)

[5.3 Study Drug Dosing Regimen 34](#_Toc146097487)

[5.3.1 General Principles 34](#_Toc146097488)

[5.3.2 Dosing Regimen Modifications 34](#_Toc146097489)

[5.3.2.1 General Principles for Dosing Regimen Modifications 34](#_Toc146097490)

[5.3.2.2 Dose Modification 35](#_Toc146097491)

[5.4 Concomitant Medications and Treatments 40](#_Toc146097492)

[5.4.1 Concomitant Medications 40](#_Toc146097493)

[5.4.1.1 Prohibited concomitant medications 40](#_Toc146097494)

[5.4.1.2 Cautious concomitant use Drug 40](#_Toc146097495)

[5.4.1.3 Permitted concomitant medications 41](#_Toc146097496)

[5.4.2 Concomitant Procedures 41](#_Toc146097497)

[5.4.3 Concomitant Medication and Treatment Collection Requirements 41](#_Toc146097498)

[6 STUDY ASSESSMENTS AND PROCEDURES 42](#_Toc146097499)

[6.1 Study Procedures and Notes 42](#_Toc146097500)

[6.2 Visit content and requirements 44](#_Toc146097501)

[6.2.1 Baseline characteristics of subjects 44](#_Toc146097502)

[6.2.1.1 Demographic data and past medical history 44](#_Toc146097503)

[6.2.1.2 Vital Signs 45](#_Toc146097504)

[6.2.1.3 Physical examination 46](#_Toc146097505)

[6.2.1.4 B Symptom assessment 46](#_Toc146097506)

[6.2.2 Laboratory Tests 46](#_Toc146097507)

[6.2.3 Cardiac function test 48](#_Toc146097508)

[6.2.4 Imaging examination 48](#_Toc146097509)

[7 EFFICACY ASSESSMENTS 49](#_Toc146097510)

[8 Biomarker Assessments 50](#_Toc146097511)

[9 SAFETY ASSESSMENTS 50](#_Toc146097512)

[9.1 Adverse Events 50](#_Toc146097513)

[9.2 Evaluation of Adverse Events 51](#_Toc146097514)

[9.3 Adverse events Of records 51](#_Toc146097515)

[9.4 Follow-up of Adverse Events 54](#_Toc146097516)

[9.5 Criteria for judging the correlation between drugs and adverse events 55](#_Toc146097517)

[9.6 Serious Adverse Events 55](#_Toc146097518)

[9.7 Handling of Serious Adverse Events 56](#_Toc146097519)

[9.8 Unexpected Serious Adverse Events Definitely Related/Suspected to the Study Drug Reaction 56](#_Toc146097520)

[9.9 Recommendations for Symptomatic Treatment of Common Adverse Reactions 56](#_Toc146097521)

[9.10 Adverse Events of Special Interest 62](#_Toc146097522)

[10 Study Data Management 62](#_Toc146097523)

[10.1 Data Entry 63](#_Toc146097524)

[10.2 Data Verification and Review 63](#_Toc146097525)

[10.3 Data Cleaning 63](#_Toc146097526)

[10.4 Electronic Signatures 63](#_Toc146097527)

[10.5 Database Lock 63](#_Toc146097528)

[10.6 Data Transfer 63](#_Toc146097529)

[11 Statistical analysis management 64](#_Toc146097530)

[11.1 Statistical Analysis Datasets 64](#_Toc146097531)

[11.2 Statistical Analysis Plan 64](#_Toc146097532)

[11.2.1 General Principles 64](#_Toc146097533)

[11.2.2 Hypothesis test and test level 65](#_Toc146097534)

[11.3 Study Population 65](#_Toc146097535)

[11.3.1 Case Distribution 65](#_Toc146097536)

[11.3.2 Protocol Violations and Deviations 65](#_Toc146097537)

[11.3.3 Baseline Descriptive Statistics 66](#_Toc146097538)

[11.4 Efficacy Analysis 66](#_Toc146097539)

[11.4.1 Primary Efficacy Measures 66](#_Toc146097540)

[11.4.2 Secondary Efficacy Measures 66](#_Toc146097541)

[11.4.3 Listing of Individual Efficacy Data 67](#_Toc146097542)

[11.5 Safety Analysis 67](#_Toc146097543)

[11.5.1 Drug Exposure 67](#_Toc146097544)

[11.5.2 Adverse Events 67](#_Toc146097545)

[11.5.3 Laboratory Tests 68](#_Toc146097546)

[11.5.4 Vital Signs 68](#_Toc146097547)

[11.5.5 Electrocardiogram 68](#_Toc146097548)

[11.5.6 ECOG Score 69](#_Toc146097549)

[11.5.7 Concomitant Medications 69](#_Toc146097550)

[11.6 Analysis of Biomarker Assessment 69](#_Toc146097551)

[11.7 Statistical Analysis Software 69](#_Toc146097552)

[12 STUDY MANAGEMENT 69](#_Toc146097553)

[12.1 Information Disclosure 69](#_Toc146097554)

[12.2 Regulatory and Ethical Review and Approval 70](#_Toc146097555)

[12.3 Informed Consent 70](#_Toc146097556)

[12.4 Protocol Amendments 70](#_Toc146097557)

[12.5 Study Protocol Deviations 71](#_Toc146097558)

[12.6 Study Documentation 71](#_Toc146097559)

[12.7 Study Termination 71](#_Toc146097560)

[12.8 End of Study 71](#_Toc146097561)

[12.9 Quality Control and Assurance 71](#_Toc146097562)

[13. Investigator's Division of the Sponsor 72](#_Toc146097563)

[13.1 Sponsor 72](#_Toc146097564)

[13.2 Investigators 72](#_Toc146097565)

[14 Publication and patent of study results 73](#_Toc146097566)

[15 REFERENCES 73](#_Toc146097567)

[Appendix I Calculation formula 75](#_Toc146097568)

[Appendix II Eastern Cooperative Oncology Group Performance Status Scoring Criteria (ECOG score) 75](#_Toc146097569)

[Appendix III New York Heart Association (NYHA) Functional Classification 75](#_Toc146097570)

[Appendix IV Definition of Relapsed/Refractory Follicular Lymphoma 76](#_Toc146097571)

[Appendix V 2014 Lugano Staging Criteria 77](#_Toc146097572)

[Appendix VI Follicular Lymphoma International Prognostic Index 2 (FLIPI-2) 77](#_Toc146097573)

[Appendix VII 2014 Lugano Evaluation Criteria 79](#_Toc146097574)

Study Abstract

| Study No. | TQ-B3525-II-01 |
| --- | --- |
| Study title | A single-arm, multicenter phase II clinical trial of TQ-B3525 in the treatment of relapsed/refractory follicular lymphoma (FL) |
| Study Drug Registration Classification/  Phase | Chemical drug class 1  Phase II |
| Sponsor | Chia Tai Tianqing Pharmaceutical Group Co., Ltd. |
| Study site/  Principal Investigator | Tianjin Union Medical Center of Nankai University, Professor Huaqing Wang;  Sun Yat-sen University Cancer Center, Professor Zhiming Li |
| Statistical analysis unit of clinical study data | Department of Biostatistics, School of Public Health, Nanjing Medical University |
| Study objectives | Primary objective  To assess the effectiveness of TQ-B3525 in treating patients with relapsed/refractory follicular lymphoma.  Secondary objectives  To assess the safety of TQ-B3525 in patients with relapsed/refractory follicular lymphoma.  To assess biomarkers related to efficacy, mechanism of action/resistance mechanism, safety of TQ-B3525. |
| Study Endpoints | Primary study endpoint:  Objective Response Rate (ORR) by IRC  Secondary study endpoints:  Efficacy  Objective response rate (ORR), duration of response (DOR), progression-free survival (PFS), disease control rate (DCR), overall survival (OS) assessed by the investigator  Safety  Includes: Adverse Reactions, Adverse Events, and Serious Adverse Events, and Withdrawals for Safety or Tolerability  Biomarker  To assess genetic changes associated with PI3K pathway and its bypass in tissue and/or plasma of patients |
| Study Design | This was a single-arm, multicenter phase II clinical ttrial to evaluate the efficacy and safety of TQ-B3525 in patients with relapsed/refractory follicular lymphoma who have failed verall survival (OS) assessed by the investigator accordance with the laws of Cht meeting exclusion criteria, subjects entered the treatment period and received the treatment regimen of TQ-B3525 tablets 20 mg once daily every 28 days until disease progression/intolerance.  This study was divided into 2 stages, the first stage was a single-arm, exploratory study; the second stage was a pivotal registration study. The second stage of the statistical analysis of effectiveness was performed on subjects.  The primary efficacy analysis was performed after the last subject in stage II had a first efficacy assessment, or the subject withdrew consent, or started other antineoplastic therapy, or died, whichever occurred first. |
| Study subjects | Patients with relapsed/refractory follicular lymphoma who have received , or started systemic therapies |
| Inclusion and exclusion criteria | Inclusion Criteria:  Patients who meet all of the following inclusion criteria can be included in this trial:   1. Subjects voluntarily participate in the study and sign the informed consent form; 2. Male or female, aged participate in the study and sign the informed consent form; trial 3. Histopathologically confirmed diagnosis of grade 1-3a follicular lymphoma (FL); 4. Relapsed/refractory FL patients who have received fractory FL f grade 1-3a ic therapies (at least 1 regimen containing rituximab or other anti-CD20 monoclonal antibodies) (refer to Appendix IV for the definition of"relapsed/refractory"); 5. Presence of at least one radiographically measurable nodal or perilymphatic malignancy by CT or MRI (intranodal lesions are defined as > 15 mm in long diameter, extranodal lesions are > 10 mm in long diameter); 6. Major organ function meeting the following criteria at screening:  - Routine blood tests (no use of growth factors or received blood transfusions within 7 days ): - Absolute neutrophil count (ANC) ≥ 1.0 × 10^9^/L; - Lymphocyte count (LYM) ≥y0.5 × 10^9^/L; - CD4 + T lymphocyte count ≥ 0.2 × 10^9^/L; - Platelet (PLT) ≥ 75 × 10^9^/L (patients with lymphoma bone marrow infiltration are allowed to be included if PLT owed to b^9^/L); - Hemoglobin (Hb) 0e include - Blood biochemistry criteria: - Alanine aminotransferase (ALT) and aspartate aminotransferase (AST) in lon× upper limit of normal (ULN) (Norm× ULN for patients with lymphoma involving the liver or biliary obstruction); - Serum total bilirubin (TBIL) ma involving - Serum creatinine (Cr) (TBIL) ma involving the liver or biliary obst ml/min (Refer to Appendix 1 for the calculation formula of creatinine clearance); - Coagulation test criteria: - Activated partial thromboplastin time (APTT), international normalized ratio (INR), prothrombin time (PT) for the defin  1. Females should agree to use contraception (intrauterine device [IUD], pill, or condom) during the study and for 6 months after the end of the study; female subjects have a negative serum or urine pregnancy test within 7 days prior to study entry and must be non-lactating; and males should agree to use contraception during the study and for 6 months after the end of the study.   Exclusion Criteria:  Subjects with any of the following will not be enrolled in the study:   1. Known follicular lymphoma to diffuse large B-cell lymphoma transformation; 2. Patients with known central nervous system (CNS) involvement of lymphoma; 3. Prior treatment with other PI3K inhibitors or CAR-T; 4. Patients who have experienced or currently suffer from other malignant tumors within 3 years, except for cured cervical carcinoma in situ, non-melanoma skin cancer and superficial bladder tumors [Ta (non-invasive tumor), Tis (carcinoma in situ) and T1 (tumor infiltrating basal lamina)]; 5. Type I and type II diabetes mellitus, unless one of the following conditions is met:  - Patients with type 2 diabetes mellitus controlled by exercise and diet only, fasting blood glucose < 7.0 mmol/L and glycosylated hemoglobin (HbA1c) < 7.0% during screening period; - Patients with type 2 diabetes mellitus who need only oral administration of single hypoglycemic agent for stable control of blood glucose, have fasting blood glucose < 7.0 mmol/L and glycosylated hemoglobin (HbA1c) < 7.0% during screening period;  1. History of interstitial lung disease, severely impaired lung function, severe pulmonary fibrosis, drug-induced lung disease (as judged by the investigator), and evidence of active pulmonary inflammation by chest CT at screening; 2. Patients requiring systemic drug therapy (prophylactic drug therapy is not excluded if there is no active infection); 3. Cytomegalovirus (CMV) infection (positive CMV PCR test result during the screening period); 4. Patients with a history of immunodeficiency, including but not limited to HIV positive or other acquired, congenital immunodeficiency diseases, or a history of active autoimmune diseases/autoimmune diseases; 5. Patients with multiple factors affecting oral administration and drug absorption (such as inability to swallow, post-gastrointestinal resection, ulcerative colitis, symptomatic/inflammatory bowel disease, chronic diarrhea, intestinal obstruction, and other gastrointestinal diseases); 6. Patients with toxicity caused by previous treatment that is not recovered to ≤ CTCAE grade 1 (excluding alopecia; for hematological toxicity, refer to Inclusion Criteria No.6); 7. Treatment with systemic steroid hormone (prednisone or equivalent at a dose equivalent to > 10 mg/day) within 7 days prior to the first dose, with the following exceptions: ① topical, intraocular, intra-articular, intranasal or inhaled corticosteroids are allowed; ② short-term corticosteroids are allowed for prevention (such as allergy to contrast agent) or treatment of non-autoimmune conditions (such as delayed hypersensitivity caused by contact allergen); 8. Subjects who received other systemic anti-tumor drug treatments within the 4 weeks preceding the first administration of the study drug or are still within the 5 half-life periods of the drug (whichever occurs first); 9. Major surgical procedures or significant traumatic injury that is not healed before the first dose; 10. Patients with evidence or history of bleeding diatheses; any entsAE grade 3 bleeding events (such as gastrointestinal bleeding, perforation, etc.) within 4 weeks before the first dose; 11. Patients who received autologous hematopoietic stem cell transplantation within 3 months before the first dose, or allogeneic hematopoietic stem cell transplantation or organ transplantation (except corneal transplantation); 12. Patients with eceived 2 cardiovascular disease within 6 months prior to the first dose, including unstable angina, myocardial infarction, arrhythmia requiring treatment, congestive heart failure (refer to Appendix III for New York Heart Association (NYHA) functional classification), cerebrovascular accident (including transient ischemic attack); 13. QTc > 480 ms (refer to Appendix 1 for QTc calculation formula), or LVEF < 50% during the screening period; 14. Urinalysis results in the screening period show urine protein 50% during the screening period; ; eriod; d; ction, > 1.0 g within 7 days; 15. The results of epidemiological test during the screening period meet any of the following criteria:  - HBsAg positive and HBV DNA exceeds the ULN (those who decrease to within the normal range after antiviral therapy can be included); - Anti-HCV positive;  1. Patients with a history of psychotropic drug abuse and unable to quit or with mental disorders; 2. Other conditions considered inappropriate by the investigator. |
| Study drug | Drug name: TQ-B3525  Dosage Form: Tablet  Drug specification: 5 mg/tablet, 20 mg/tablet |
| Dosing Regimen | Oral administration (fasting or fed, at a relatively fixed time in the morning), 20 mg once daily for 28 days as one cycle. If patients benefited (CR, PR or SD) based on efficacy evaluation, treatment should be continued until disease progression or intolerable toxicity. When CR was achieved, treatment continued at 1 or 2 dose levels lower (down to which dose level at the discretion of the investigator). The dose levels of study drug were as follows:   \| TQ-B3525 tablets \| \| \| --- \| --- \| \| Starting dose level \| 20 mg QD \| \| Dose Level -1 \| 15 mg QD \| \| Dose level-2 \| 10 mg QD \| |
| Study period | Planned start time: January 2020  Planned end: December 2021 |

1 Study background

- 1. Disease background

Lymphomas are a heterogeneous group of malignancies arising from lymphoid tissue (1). Approximately 85% of lymphomas are non-Hodgkin's lymphomas (NHL) and have different subtypes, clinical features, and response to therapy. Most NHLs originate from B lymphocytes (85% - 90%) and can be classified based on cell origin, tumor proliferation rate, and tissue student long type (2).

Non-Hodgkin's lymphoma has different clinical characteristics and can be divided into two prognostic groups according to its clinical behavior: aggressive NHL and indolent NHL. Aggressive NHLs are characterized by high tumor proliferation, symptomatic disease, and can lead to death if left untreated (3), and complete cure can be achieved in 50% of patients using immunochemotherapy regimens and stem cell transplantation. Indolent NHLs present clinically with few symptoms and low tumor proliferation, have a relatively good prognosis and a survival of more than 10 years, but are incurable with currently available treatment options, especially in the advanced stages of the disease. Although indolent NHLs respond well to standard chemotherapy regimens and radiotherapy, their natural course is characterized by a continuous pattern of relapse, and although most relapses can usually be successfully treated, their quality of response gradually decreases and the time to the next relapse gradually decreases, eventually evolving into refractory disease or into aggressive NHL, with a poor prognosis.

Follicular lymphoma (FL), the most common indolent non-Hodgkin's lymphoma, accounts for 35% of NHL cases in North America, 9% to 22% of NHL in the world, and 8.1% to 23.5% of NHL in China, and is an indolent B-cell tumor derived from follicular germinal center cells, and its incidence is increasing year by year (4). Although the degree of invasion is low, it is easy to transform into diffuse large B-cell lymphoma (DLBCL) and has a high recurrence rate after remission, which is still an incurable disease. Therefore, improving ORR and prolonging PFS in relapsed patients is the main goal of current treatment.

FL patients with initially low tumor burden can receive rituximab or rituximab combined with chemotherapy (R-chemo); while patients with high tumor burden can receive R-chemo, which can be followed by rituximab maintenance therapy or no maintenance therapy. Although most patients will respond to these first-line therapies, the natural course of FL is characterized by a sustained relapse. Analysis of 588 FL patients who received R-CHOP as initial therapy showed that approximately 20% experienced early (within 2 years of diagnosis) progressive disease (PD), and the 5-year overall survival (OS) in this population was only 50% [5]. At present, the standard treatment for relapsed/refractory follicular lymphoma has not been unified in China. R-CHOP, R-CVP and rituximab + lenalidomide are commonly used regimens in clinical practice. However, there are still unmet needs for the treatment of such patients. How to improve the ORR and PFS of FL patients and inhibit the transformation of FL to DLBCL has become the key to improve the quality of life of FL patients. At present, it is difficult for conventional treatment regimens to achieve this purpose. The emergence of many new drugs provides more options for the treatment of patients, such as PI3K inhibitors.

Phosphatidylinositol 3-kinases (PI3Ks) are lipid kinases that control cell proliferation, survival, apoptosis, etc. PI3K transmits the signals of various growth factors and cytokines into cells by generating activated AKT and downstream phospholipids. The PI3K family can be divided into three classes, which vary in structure and function. Among them, the most widely studied or important is class I PI3K, which consists of a regulatory subunit P85 and a catalytic subunit P110. There are four catalytic subunits, namely, P110 αhosphatidylinositol 3-kinases (PI3Ks) are lipid kinases that control cell pr αhosphatidylinositol 3-kinases (PI3Ks) are lipid kinases that control cell proliferation, survival, apoptosis, etc. PI3K transmits the signals of various growth factors and cytopleckstrin homology domain) domain, prompting PDK1 to phosphorylate Ser308 and Ser473 of AKT protein, resulting in the activation of AKT. Activated AKT activates the mTOR complex by phosphorylating downstream factors such as a variety of enzymes, kinases, and transcription factors, ultimately leading to the expression of a variety of cellular functional genes, including the entire cell growth cycle, metabolism, ribosome biogenesis, RNA transcription, and apoptosis. At the same time, PIP3 is also a substrate of tumor suppressor (PTEN, phosphatase and tensin homology deleted on chromosome 10), a PIP3-phosphatase, which can convert PIP3 to PIP2 by dephosphorylation and reduce AKT activation and prevent all downstream signaling events regulated by AKT [7 - 8].

PI3Kα and PI3Kβ are expressed in various organs, while PI3Kδ and PI3Kγ are mainly distributed in bone marrow cells. The physiological functions of the four isoforms are also different, each of which regulates different physiological functions. PI3Kα mediates the metabolic insulin signal transmission in tissues, PI3Kβ is involved in the regulation of integrin signaling in platelets, and PI3Kγ and PI3Kδ regulate different protein signals in leukocytes, especially in mast cells, neutrophils and eosinophils. The PI3K signal transduction pathway not only directly mediates the physiological regulation of cells, but also is directly related to many diseases. It has been found in a large number of solid tumors that amplification and activating mutations in PIK3CA, the gene encoding PI3Kα, result in increased catalytic capacity of PI3Kα; thrombosis is closely related to activation of PI3Kβ, which has been confirmed as an important target for antithrombotic therapy, and there is ongoing evidence that inhibitors of PI3Kβ have great potential in the treatment of autoimmune diseases, and PI3Kβ also plays a role in the development and progression of some tumors; PI3Kδ has been found to play a central role in the recruitment and activation of a wide range of immune and inflammation-related cells and is a key target for the treatment of hematological malignancies such as acute myelogenous leukemia, and the PI3Kδ signaling pathway is often hyperactivated in B-cell cancer cells, making PI3K inhibitors an effective treatment for indolent non-Hodgkin lymphoma (iNHL), while the B-cell receptor signaling pathway also plays an important role in the pathology of chronic lymphocytic leukemia (CLL), the most critical subtype of which is associated with the malignant phenotype. PI3KPI3Ks clos to be abnormally expressed in inflammatory and immune diseases and has great potential in the targeted therapy of inflammation (9).

In recent years, inhibitors targeting this target have become a hot spot in anti-tumor drug research and development. As of November 2019, there have been a total of nearly 50 PI3K inhibitors under clinical development worldwide, mainly for lymphoma and solid tumors, while 4 PI3K inhibitors have been approved by FDA for marketing abroad, mainly for the treatment of lymphoma. See the table below for the basic information of the marketed drugs. It can be seen from the indications that PI3K inhibitors are very effective in the treatment of relapsed/refractory indolent lymphoma, especially follicular lymphoma, but there is no PI3K inhibitor marketed in China.

Table 1 Basic information of PI3K inhibitors that have been approved by FDA for marketing abroad

| Drug | Time | Target of Action | Indications for Use |
| --- | --- | --- | --- |
| Idelalisib [10] | 2014 | PI3Kδ | Relapsed/refractory FL, CLL/SLL |
| Copanlisib [11] | 2017 | PI3Kα/δ | Relapsed/refractory FL |
| Duvelisib [12] | 2018 | PI3Kδ/γ | Relapsed/refractory FL, CLL/SLL |
| Alpelisib [13] | 2019 | PI3Kα | Hormone receptor positive breast cancer (PIK3CA mutated) |

- 1. Drug profile

TQ-B3525 is a novel dual PI3Kα/δ inhibitor developed by Chia Tai Tianqing Pharmaceutical Group Co., Ltd., which can overcome the resistance problem caused by the up-regulation of PI3Kα subunit activity caused by PI3Kδ subunit inhibition alone, and is intended for the treatment of lymphoma and solid tumors.

TQ-B3525 has completed various preclinical studies, and the results showed strong selective inhibition of PI3Kα/δ subunit. Compared with the preclinical kinase activity of drugs with the same target, the in vitro IC50 value of TQ-B3525 was much lower than that of marketed idelalisib and duvelisib, and comparable to the IC50 level of copanlisib. The preclinical pharmacological and toxicological results of TQ-B3525 showed that it had no significant abnormal effect on the cardiovascular system and respiratory system; in the long-term toxicity test in rats and cynomolgus monkeys, the main target organs were gastrointestinal tract, liver, immune hematopoietic organs (spleen, thymus), etc., which returned to normal after drug withdrawal for 28 days, and there was no accumulation of plasma concentrations and parameters after continuous administration. The clinical results showed that TQ-B3525 had high bioavailability in crabs, was mainly metabolized by CYP3A4, and had no inhibitory effect on P450 enzymes.

A total of 60 subjects (solid tumors and lymphoma) have been enrolled in the phase 1 clinical study of TQ-B3525 (NCT03510767) as of November 2020. Twenty of these were evaluable for follicular lymphoma (FL), for an ORR of 75% (15/20). The Grade 3 adverse reaction and dose-limiting toxicity (DLT) related to the study drug was hyperglycemia, which could be controlled and recovered after drug withdrawal or symptomatic treatment. Other major adverse reactions are shown in Table 2 below. The kinetic results showed that the elimination half-life T1/2 of TQ-B3525 was about 10-12h, and the time to peak Tmax was about 1-2 h. In the dose range of 2 mg QD to 20 mg QD and multiple doses, Cmax and AUC increased linearly with increasing dose.

Table 2 Adverse Reactions Occurring in d lymphoma) have been enrolled in the phase 1 clinica

| Most Common Adverse Reactions (≥ 20%) | 2 mg QD to 20 mg BID dose groups (n = 60) | |
| --- | --- | --- |
|  | All Grades n (%) | Grade ades n (%) |
| Hyperglycaemia | 36 (60.0%) | 6 (10.0%) |
| Glycosylated haemoglobin increased | 23 (38.3%) | - |
| Diarrhea | 23 (38.3%) | 2 (3.3%) |
| Neutrophil count decreased | 15 (25.0%) | 5 (8.3%) |
| White blood cell count decreased | 15 (25.0%) | 1 (1.7%) |
| Proteinuria | 14 (23.3%) | - |
| Alanine aminotransferase increased | 13 (21.7%) | - |
| Lymphocyte count decreased | 12 (20.0%) | 4 (6.7%) |
| Asthenia | 12 (20.0%) | - |

- 1. Phase I clinical study phase summary

As of November 2020, the results of the phase I clinical study of TQ-B3525 tablets showed significant efficacy in subjects with relapsed/refractory non-Hodgkin's lymphoma, especially follicular lymphoma (FL), with an ORR of 79% (15/19) for FL in the effective dose range (≥ 10 mg QD). The adverse reactions of TQ-B3525 were generally consistent with those of other similar PI3K inhibitors, and the main dose-limiting toxicity was hyperglycemia, a reversible adverse reaction caused by inhibition of PI3Kα targets, which was controlled and returned to baseline levels by discontinuation or symptomatic treatment.

As of November 2020, the 20 mg QD dose group in the phase I study was well tolerated. The main Grade 3 adverse reactions related to the study drug were hyperglycemia, neutrophil count decreased, diarrhea, hypokalemia, pneumonia, lung infection, platelet count decreased, etc. At the same time, 10 FL patients in this dose group had a good response (ORR = 80%). Therefore, 20 mg QD was selected as the recommended starting dose for this study.

Based on the above study data, it is planned to develop the indication of relapsed/refractory follicular lymphoma for the TQ-B3525 variety.

2 Study objectives and endpoints

2.1 Study Objectives

- Primary objective:
- To assess the effectiveness of TQ-B3525 in treating patients with relapsed/refractory follicular lymphoma.
- Secondary objectives:
- To assess the safety of TQ-B3525 in patients with relapsed/refractory follicular lymphoma.
- To assess biomarkers related to efficacy, mechanism of action/resistance mechanism, safety of TQ-B3525.

2.2 Study Endpoints

- Primary study endpoints:
- Objective response rate (ORR): The proportion of patients who are evaluated as PR or CR at any time during study drug treatment by the Independent Radiographic Evaluation Committee (IRC) according to the 2014 version of the Lugano evaluation criteria.
- Secondary study endpoints:
- Objective response rate (ORR) by investigator: the proportion of patients with PR or CR at any time during study drug treatment as assessed by the investigator according to the 2014 version of the Lugano evaluation criteria.
- Duration of Response (DOR): The time from the first evaluation of CR or PR until the first evaluation of PD or death, whichever occurs first.
- Progression-free survival (PFS): The time from the first dose to disease progression or death, whichever occurs first.
- Disease control rate (DCR): the proportion of evaluable patients with response (PR + CR) and stable disease (SD) after treatment.
- Overall survival (OS): time from first dose to death due to any cause.
- Safety: including adverse reactions, adverse events and serious adverse events, as well as withdrawal from the trial due to safety or tolerability.
- Biomarkers: To assess changes in genes involved in the PI3K pathway and its bypass in patient tissue and/or plasma.

3 Study Design

3.1 Overall Design

1. A single-arm, multicenter phase II clinical study to evaluate the efficacy and safety of TQ-B3525 in patients with relapsed/refractory follicular lymphoma who have failed ≥ 2 prior lines of treatment. After screening, meeting the inclusion criteria and none of the exclusion criteria, subjects entered the treatment period and received the treatment regimen of TQ-B3525 tablets 20 mg QD every 28 days until disease progression/intolerance.

The study is divided into two stages. The first stage is a single-arm, exploratory study; the second stage is the key registration study. The efficacy statistical analysis is performed for the subjects in the second stage.

The primary efficacy analysis was performed after the last subject in Stage II had a first efficacy assessment, or the subject withdrew consent, or started other antineoplastic therapy, or died, whichever occurred first.

3.2 Rationale for selection of primary study endpoints

This trial uses objective response rate (ORR) as the primary study endpoint. The Technical Guidelines for Clinical Trials of Antineoplastic Drugs state that ORR is a preliminary and reliable basis for the antitumor activity of responsive drugs and is the main efficacy outcome measure commonly used in phase II clinical studies. In FDA accelerated approval, ORR is also the most commonly used surrogate endpoint, and the observation of ORR in a single-arm study can provide sufficient basis to support accelerated approval. Therefore, ORR was selected as the primary study endpoint in this study.

3.3 Sample Size Design

The first phase of this study was a single-arm, exploratory study with no more than 50 subjects planned.

The second stage is a single-arm, multi-center designed pivotal registration study. The primary efficacy endpoint of this study was objective response rate (ORR). Referring to previous clinical studies, the ORR of historical treatment in the target population of this study is 40%, and it is expected that the ORR of the investigational drug will increase to 60%. Assuming that the power (1-β) is 90%, the effectiveness hypothesis test (H0: ORR ≤ 40%, H1: ORR > 40%) is performed at one-sided α = 0.025 level. According to the above parameters, the exact probability method is selected by PASS 2019 software for calculation. The sample size is required to be 64. Considering 20% dropout rate, 80 subjects are planned to be enrolled in the second stage of the study.

4 Study subjects

Patients with relapsed/refractory follicular lymphoma who have received tion study. Tsystemic therapy (refer to Appendix IV for the definition of"relapsed/refractory").

4.1 Inclusion Criteria

Patients who meet all of the following inclusion criteria can be included in this trial:

1. Subjects voluntarily participate in the study and sign the informed consent form;
2. Male or female, aged partiyears; ECOG (PS) score: 0 ~ 2; expected survival form; trial
3. Histopathologically confirmed diagnosis of grade 1-3a follicular lymphoma (FL);
4. The subject has relapsed/refractory FL who has received FL); lar lymphoma (FL); onths; : emic therapy (refer to Aontaining rituximab or other anti-CD20 monoclonal antibodies) (refer to Appendix IV for the definition of"relapsed/refractory");
5. Presence of at least one radiographically measurable nodal or perilymphatic malignancy by CT or MRI (intranodal lesions are defined as > 15 mm in long diameter, extranodal lesions are > 10 mm in long diameter);
6. Major organ function meeting the following criteria at screening:

- Routine blood tests (7 without growth factors or blood transfusion):
- Absolute neutrophil count (ANC) ≥ 1.0 × 10^9^/L;
- Lymphocyte count (LYM) nt (ANC) ≥ 0.5 × 10^9^/L;
- CD4 ^+^ T lymphocyte count ≥ 0.2 × 10^9^/L;
- Platelet (PLT) ≥ 75 × 10^9^/L (patients with lymphoma bone marrow infiltration ≥ 50 × 10^9^/L are allowed to be included);
- Hemoglobin (Hb) o be inclu
- Blood biochemistry criteria:
- Alanine aminotransferase (ALT) and aspartate aminotransferase (AST) n long diameter, by CT or MRI (intrith lymphoma involving the liver or biliary obstruction);
- Serum total bilirubin (TBIL) ) and aspartatSerum creatinine (Cr) (TBIL) ) and aspartate aminotransferase (AST) n long diameter, by CT or MRI (intrith lymphoma involving the liver or biliary
- Coagulation test criteria:
- Activated partial thromboplastin time (APTT), international normalized ratio (INR), prothrombin time (PT) AST) n long

1. Females should agree to use contraception (intrauterine device [IUD], pill, or condom) during the study and for 6 months after the end of the study; have a negative serum or urine pregnancy test within 7 days prior to study entry and must be non-lactating subjects; and males should agree to use contraception during the study and for 6 months after the end of the study.

4.2 Exclusion criteria

Subjects with any of the following will not be enrolled in the study:

1. Known follicular lymphoma to diffuse large B-cell lymphoma transformation;
2. Patients with known central nervous system (CNS) involvement of lymphoma;
3. Prior treatment with other PI3K inhibitors or CAR-T;
4. Patients who have experienced or currently suffer from other malignant tumors within 3 years, except for cured cervical carcinoma in situ, non-melanoma skin cancer and superficial bladder tumors [Ta (non-invasive tumor), Tis (carcinoma in situ) and T1 (tumor infiltrating basal lamina)];
5. Type I and type II diabetes mellitus, unless one of the following conditions is met:

- Patients with type 2 diabetes mellitus controlled by exercise and diet only, fasting blood glucose < 7.0 mmol/L and glycosylated hemoglobin (HbA1c) < 7.0% during screening period;
- Patients with type 2 diabetes mellitus who need only oral administration of single hypoglycemic agent for stable control of blood glucose, have fasting blood glucose < 7.0 mmol/L and glycosylated hemoglobin (HbA1c) < 7.0% during screening period;

1. History of interstitial lung disease, severely impaired lung function, severe pulmonary fibrosis, drug-induced lung disease (as judged by the investigator), and evidence of active pulmonary inflammation by chest CT at screening;
2. Patients requiring systemic drug therapy (prophylactic drug therapy is not excluded if there is no active infection);
3. Cytomegalovirus (CMV) infection (positive CMV PCR test result during the screening period);
4. Patients with a history of immunodeficiency, including but not limited to HIV positive or other acquired, congenital immunodeficiency diseases, or a history of active autoimmune diseases/autoimmune diseases;
5. Patients with multiple factors affecting oral administration and drug absorption (such as inability to swallow, post-gastrointestinal resection, ulcerative colitis, symptomatic/inflammatory bowel disease, chronic diarrhea, intestinal obstruction and other gastrointestinal diseases);
6. Patients with toxicity caused by previous treatment that is not recovered to ≤ CTCAE grade 1 (excluding alopecia; for hematological toxicity, refer to Inclusion Criteria No.6);
7. Treatment with systemic steroid hormone (prednisone or equivalent at a dose equivalent to > 10 mg/day) within 7 days prior to the first dose, with the following exceptions: a. topical, intraocular, intra-articular, intranasal or inhaled corticosteroids are allowed; b. short-term corticosteroids are allowed for prevention (such as allergy to contrast agent) or treatment of non-autoimmune conditions (such as delayed hypersensitivity caused by contact allergen);
8. Patients who have received other systemic anti-tumor drug treatment before the first dose, or still within 5 half-lives of the drug (whichever occurs first);
9. Major surgical procedures or significant traumatic injury that is not healed before the first dose;
10. Patients with evidence or history of bleeding diatheses; Any ≥ CTCAE grade 3 bleeding events (such as gastrointestinal bleeding, perforation, etc.) before the first dose;
11. Patients who received autologous hematopoietic stem cell transplantation within 3 months before the first dose, or allogeneic hematopoietic stem cell transplantation or organ transplantation (except corneal transplantation);
12. Patients with eceived 2 cardiovascular disease within 6 months prior to the first dose, including unstable angina, myocardial infarction, arrhythmia requiring treatment, congestive heart failure (refer to Appendix III for New York Heart Association (NYHA) functional classification), cerebrovascular accident (including transient ischemic attack);
13. QTc > 480 ms (refer to Appendix 1 for QTc calculation formula), or LVEF < 50% during the screening period;
14. Urinalysis results in the screening period show urine protein , or LVEF < 50% during the screening period; rction,> 1.0 g within 7 days;
15. When the results of epidemiological test during the screening period show that any of the following conditions are met:

- HBsAg positive and HBV DNA exceeds the upper limit of normal (those who decrease to within the normal range after antiviral therapy can be included);
- Anti-HCV positive;

1. Patients with a history of psychotropic drug abuse and unable to quit or with mental disorders;
2. Other conditions considered inappropriate by the investigator.

4.3 Screen Failure

The maximum allowed time interval between signing informed consent and starting treatment is 28 days, and failure to meet the study criteria beyond 28 days is considered a screening failure. Screen failure subjects may be re-screened after appropriate treatment or observation at the investigator's discretion, and re-screened subjects will be assigned a new screening number and sign a new informed consent form. Each subject may be rescreened up to once.

4.4 Subjects Numbering principle

According to the sequence of signing informed consent form, subjects will be numbered sequentially with screening number. Principle of screening number: 2-digit center number + 3-digit serial number. For example, for the first subject screened at Site 01, the screening number is 01001.

After confirming that the subjects meet the inclusion criteria and do not meet the exclusion criteria, the subjects will be numbered sequentially with the inclusion number. Principle of inclusion number: c + 2 site number + 3 serial number. For example, for the first subject at site 01, the enrollment number is c01001, and the subject enrollment number withdrawn halfway will not be assigned to other subjects.

4.5 Withdrawal Criteria

If the enrolled subject develops any condition that makes it inappropriate to continue the study during the study, the investigator has the right to decide whether the subject should withdraw from the study or not, and the subject is not willing to continue to participate in the clinical study. According to the provisions of the informed consent form, the subject has the right to withdraw from the study at any stage of the study without discrimination or retaliation; or the subject is not clearly withdrawn from the study, but lost to follow-up due to no further medication or testing, it is also a "withdrawal" or "dropout". There are several main reasons for withdrawal:

1. Disease progression;
2. Adverse events;
3. Voluntary withdrawal of subjects;
4. Protocol violation;
5. Death;
6. Other reasons.

4.6 Post-exit treatment

The investigator should clearly collect the reasons and time for the withdrawal of subjects, perform the corresponding observation and evaluation for the withdrawn subjects, complete the evaluation content of withdrawal specified in the study plan, fill in the corresponding original records and describe the reasons for withdrawal.

Subjects who discontinue from the study should continue to be followed for adverse events (AEs). These subjects should continue to be followed for AEs, if possible, until they have returned to baseline or the Investigator deems follow-up observation unnecessary. If the adverse event is a chronic condition, the investigator may agree that no further follow-up is required. In this case, the investigator must record his/her opinion in the subject's original medical record and inform the sponsor of the subject's discontinuation as soon as possible.

During the study period, all subjects who receive administration but fail to complete the relevant study contents in the protocol shall be transferred from the last test results to the final results. The investigator should contact the subjects who fail to make return visit as required, and record the contact evidence in the corresponding original records and eCRF (such as: time and date of telephone contact, posting receipt of registered letter, etc.), and properly preserve it.

Subsequent data collection is not required for withdrawals due to:

1) Subject withdraws informed consent;

2) Death of the subject;

3) Study termination by sponsor.

5 Study drug

5.1 General Information of Study Drug

The package of the investigational drug is printed with the name, strength, package, usage and dosage, storage conditions, batch number, shelf life, manufacturer and other information of the investigational drug, and marked with "for clinical study only". The sample manuscript of the label is as follows, subject to actual use.

Table 3 Basic information of study drug

| Drug name | Strength | Storage conditions | Batch number and shelf life | Manufacturer |
| --- | --- | --- | --- | --- |
| TQ-B3525 tablets | 5 mg/tablet  20 mg/tablet | Sealed and stored below 25e | See CoA for details | Chia Tai Tianqing Pharmaceutical Group Co., Ltd. |

| Approval Letter No.: 2017L04051/3 Study No.: TQ-B3525-II-01 | |
| --- | --- |
| TQ-B3525 Clinical Investigational Product (Follicular Lymphoma) | |
| (For clinical study use only) | |
| Strength: 5 mg/tablet or 20 mg/tablet | Storage condition: Sealed and stored below 25ll |
| Package: × × | Batch No.: × × |
| DOSAGE AND ADMINISTRATION: 1 tablet once daily,  Oral | Valid to: × × |
| Chia Tai Tianqing |  |

Sample of IP label

5.2 Study Drug Management

The sponsor will provide the study drug in sufficient quantity to complete the study. According to the requirements of GCP, the study drugs were kept by the drug administrator specifically, stored under appropriate storage conditions, dispensed according to the prescription/doctor's advice, and the last remaining study drugs were recovered. The distribution and recovery of study drug should be recorded completely.

For subjects who met the protocol requirements, the drug was prescribed/prescribed by the investigator; the drug manager dispensed the study drug according to the prescription/medical order and labeled the subject inclusion number on the drug package.

When dispensing the study drug for the first time, a certain amount should be dispensed to ensure that the subject has enough drug to take. During the study, when the subject returns to the study site, the investigator will dispense the study drug for the next stage to the subject according to the drug use of the subject and the visit frequency of the subject, return the remaining packages, and return all the remaining drugs and packages of the subject at the last treatment visit.

The remaining drugs not dispensed to the subjects or recovered will be periodically counted and recovered by the sponsor, and the investigator should cooperate with the sponsor to complete this work.

5.3 Study Drug Dosing Regimen

5.3.1 General Principles

Each 28-day treatment cycle will constitute one treatment cycle during the study period. If the administration is delayed for more than 3 days in each treatment cycle, it will be considered as dose delay due to reasons. Any dose delay/suspension/omission of drug will not affect the regular days in each cycle.

TQ-B3525 tablets: starting dose is 20 mg QD. Oral administration (fasting or postprandial, relatively fixed time in the morning), once daily, continuously. When CR was achieved, treatment continued at 1 or 2 dose levels lower (down to which dose level at the discretion of the investigator).

Subjects should swallow the tablets whole and not handle them before taking them, e.g., split and take them after dissolving in liquid.

- If a subject received 1 additional dose on a single day, the next dose was taken.
- If the subject vomits after administration on that day, no extra dose is allowed.
- If the subject missed the dose on that day, it should be less than 12 hours since the next dose, the subject should not take it again on that day.

The investigator should instruct the subject to record the abnormalities in administration in the diary card and describe them in the original medical records in detail. On the day of return visit, the subject should be instructed to bring the drug taken on that day back to the study site and take it after fasting examination on that day (the drug can be dispensed from the previous visit or to be dispensed at this visit).

5.3.2 Dosing Regimen Modifications

In the event of toxicity, the investigator may refer to the following rules for dose delays or modifications. The reason for delay or dose modification, supportive therapy taken and results were recorded in the subject's medical record and EDC.

If, at the discretion of the investigator, the dose modification differs from the suggested modification rule, it should be adjusted accordingly by the investigator with reference to clinical practice guidelines or previous clinical experience after communication with the sponsor, and the relevant reasons should be recorded in the subject's medical record.

5.3.2.1 General Principles for Dosing Regimen Modifications

- Toxicities should be graded according to the NCI-CTC AE 5.0 grading system;
- In case of concurrent toxicity of different severity grades, adjustments should be made according to the highest grade observed;
- If the treatment is interrupted for > 4 weeks due to toxicity caused by the drug, the subject must discontinue the study treatment. If the subject still needs to remain in the study, it is necessary to communicate with the sponsor and principal investigator and obtain the consent of the subject before continuing the study can be considered and recorded in detail;
- Before initiation of treatment in any cycle, all toxic events caused by treatment should be recovered to at least study treatment. If the subject still needs to remaiise, dose delay is required. For special circumstances beyond those specified in the protocol, the investigator should assess the rationality of medication and make a detailed record.

5.3.2.2 Dose Modification

Any subject who requires a dose reduction will continue treatment at a reduced dose in subsequent cycles. If a subject has had 2 dose reductions, study treatment must be discontinued if a third dose reduction is required due to toxicity. Re-escalation is not allowed after a dose reduction.

Table 4 Study Drug Dose Levels

| Starting dose level | 20 mg QD |
| --- | --- |
| Dose Level -1 | 15 mg QD |
| Dose level -2 | 10 mg QD |

When subjects experience study drug-related toxicity, the investigator should give corresponding symptomatic treatment according to the tolerance of the subjects and adjust the dosing regimen of the study drug if necessary. The specific recommended principles for dose adjustment are as follows. If the suggested principles are different from those provided by the sponsor, for clinical practice, the investigator is allowed to perform adjustment according to actual situation. However, the reasons for adjustment should be recorded in the original medical records in detail. Recommended principles for dose modification for relevant toxicities are as follows.

Table 5 Dose Regimen Modifications – Hematologic Toxicities

| Grading | Dosing Modification Scheme | Recommended handling measures |
| --- | --- | --- |
| - Hemoglobin decreased, Grade 3 - Grade 3 neutrophil count decreased - Grade 2 platelet count decreased (normal baseline) - Grade 3 platelet count decreased (baseline < LLN) | 1. Delay dosing until toxicity recovers to ne < LLN) xicities ve 2. Reduce by 1 dose level and continue   (For grade 2 platelet count decrease with normal baseline, continuation at the original dose may be considered after toxicity recovers to r grade 2 p | Treat symptomatically and monitor at least weekly until recovery from toxicity |
| - Grade 3 lymphocyte count decreased - Grade 3 CD4 positive lymphocyte decrease | 1. Delay dosing until toxicity recovers to t weekly untilseline 2. Reduce by 1 dose level and continue | 1. Treat symptomatically and monitor at least weekly until recovery from toxicity 2. Sulfonamides are recommended to prevent opportunistic infections due to Pneumocystis Yersiniae |
| - Hemoglobin decreased, Grade 4 - Grade 4 neutrophil count decreased - Grade 4 platelet count decreased - Grade 4 platelet count decreas - ≥rade 4 platelet count decreased d vent opportunist | 1. Delay dosing until toxicity recovers to opportunistic infectio 2. Continue treatment at 1 to 2 dose levels lower or discontinue at the discretion of the investigator | Treat symptomatically and monitor at least weekly until recovery from toxicity |

Table 6 Dosing Regimen Modifications – Elevated Fasting Blood Glucose (FBG)

| **FBG change (mmol/L)** | Dosing Modification Scheme | Recommended handling measures  (Refer also to Table 17 in Section 9.9) |
| --- | --- | --- |
| ULN er also to T | Original dose, administration continued | 1. Continuous observation, oral hypoglycemic agents can be given for symptomatic treatment 2. Prophylactic use of oral hypoglycemic agents may be considered during subsequent treatment if blood glucose levels continue to rise |
| 8.9 < FBG ic use | 1. The investigator can determine whether to delay the administration based on the fasting and fasting blood glucose levels 2. Maintain the original dose or reduce by 1 dose level, and continue medication | 1. Give oral hypoglycemic agents for symptomatic treatment; if oral hypoglycemic agents are poorly controlled, give oral hypoglycemic agents with different mechanisms of action in combination, use insulin if necessary, and repeat monitoring within 7 days until FBG ≤ 8.9 2. Pay attention to the changes of urine ketone and other indicators 3. Prophylactic use of oral hypoglycemic agents may be considered during subsequent treatment if blood glucose levels continue to rise |
| FBG > 13.9 Uncomplicated symptoms  (Duration < 3 days) | 1. Delay dosing until FBG recovers to ed dur 2. Maintain the original dose or reduce the dose by 1 dose level at the investigator's discretion, and continue medication | 1. Immediate symptomatic treatment, repeated monitoring within 7 days until FBG ator's 2. Close monitoring of FBG, urine ketone and other indicators 3. Prophylactic use of oral hypoglycemic agents or insulin for glycemic control during subsequent therapy |
| FBG > 13.9 Uncomplicated symptoms  (duration Uncomplic | 1. Delay dosing until FBG recovers to ic age 2. Reduce the dose by 1-2 dose levels at the investigator's discretion and continue treatment |  |
| FBG > 13.9 with symptoms | Treatment termination | Discontinued after symptomatic treatment |

Note: FBG refers to fasting blood glucose, including fasting blood glucose measured by laboratory tests and fasting blood glucose measured by home monitoring. At any time, if the investigator judges that the subject's blood glucose is intolerable, symptomatic hypoglycemic therapy is allowed. See Table 17 for the management measures for hyperglycemia.

Table 7 Dosing Regimen Modifications - Infection

| Grading | Dosing Modification Scheme | Recommended handling measures |
| --- | --- | --- |
| 1 | Original dose, administration continued | Continuous observation for preventive medication |
| 2 | 1. Delay until resolved to or prevent 2. Continue treatment at 1 to 2 dose levels lower at the discretion of the investigator | Symptomatic treatment |
| 3 | 1. Delay until resolved to to 2 dose 2. Continue treatment at 1 to 2 dose levels lower or discontinue at the discretion of the investigator | Symptomatic treatment |
| 4 | Treatment termination | Discontinued after symptomatic treatment |

Notes:

- In case of severe infection such as Yersinia pneumocystis pneumonia (PJP), the drug should be immediately discontinued and the study drug should be discontinued.
- For patients with CMV (cytomegalovirus) infection, herpes simplex virus infection and varicella zoster infection, regardless of grade, immediate dose interruption and symptomatic treatment are required until the infection subsides. At the discretion of the investigator, treatment may be resumed at the original dose or a reduced dose level.

Table 8 Dose Regimen Modifications – Pneumonitis (NIP)

| Grading | Dosing Modification Scheme | Recommended handling measures |
| --- | --- | --- |
| 1 | Original dose, administration continued | Continuous observation and symptomatic treatment |
| 2 | 1. Delay until resolved to nd symptom 2. Continue treatment at 1 to 2 dose levels lower at the discretion of the investigator | Symptomatic treatment |
| 3-4 | Treatment termination | Discontinued after symptomatic treatment |

Notes:

- The investigator was required to differentiate between pneumonitis (NIP), pneumonia (viral, bacterial, fungal), aspiration pneumonia, or other pneumonia that was not attributable to a study drug-related hypersensitivity reaction; and provide the basis for considering it as an infectious or other pneumonia, as appropriate. The investigator was required to report specific clinical terms addressing this case of pneumonia to the greatest extent possible and not to use only "pneumonia".
- In principle, treatment should be terminated if grade 3 pneumonitis occurs. If the patient still requires to continue the treatment, after the investigator assesses the possibility of benefit, he/she should communicate with the sponsor and PI, and the investigator should determine to reduce the dose by 1-2 doses before restarting the treatment, and record the reasons in the original medical records in detail.

Table 9 Dose Regimen Modifications – Gastrointestinal Adverse Reactions (Diarrhea/Enteritis/Nausea/Vomiting)

| Grading | Dosing Modification Scheme | Recommended handling measures |
| --- | --- | --- |
| 1-2 | Original dose, administration continued | 1. After the first appearance, prophylactic treatment (Grade 1-2) or symptomatic treatment (Grade 3-4) 2. If persistent diarrhea subsequently occurs despite prophylactic or symptomatic treatment, the possibility of concomitant medication (e.g., metformin), gastrointestinal infection (including CMV, piroplasms difficile, etc.) or gastrointestinal inflammation (by endoscopy or tissue examination, etc.) should be comprehensively evaluated |
| 3-4 | 1. For the first occurrence, delay administration until recovery to examination, etc.) should be comprehensively evaluated valuat 2. Continue treatment at 1 to 2 dose levels lower or discontinue treatment if persistent diarrhea, mprehensively evaluated valuatetions persist at the investigator's discretion |  |

Table 10 Dose Regimen Modifications vels lower or discontinue treatment if persistent diarrhea, mpr

| Grading  **(ALT/AST)** | Dosing Modification Scheme | Recommended handling measures |
| --- | --- | --- |
| 1-2 | Original dose, administration continued | 1. Continuous observation, and the investigator decides whether to perform hepatoprotective treatment 2. If accompanied by clinical symptoms, repeat monitoring to er to perform hepatoprotective treatment |
| 3-4 | 1. Delay dose until toxicity recovers to epeat moni 2. Continue at 1 dose level lower | Take hepatoprotective treatment, and repeat monitoring within 7 days until recovered to treatment |

Table 11 Dosing Regimen Modifications epeat monitoring within 7 days until recovered to treatment ehensthout Cholestasis or Hemolysis)

| Grading  **(ALT/AST)** | Dosing Modification Scheme | Recommended handling measures |
| --- | --- | --- |
| 1 | Original dose, administration continued | Repeat monitoring within 48 hours until return to normal or abnormality is not clinically significant |
| 2 | 1. Delay dose until toxicity recovers to l return t 2. Continue at 1 dose level lower | Take hepatoprotective treatment, and repeat monitoring within 7 days until recovered to y signific |
| 3-4 | Treatment termination | Discontinued after symptomatic treatment |

Table 12 Dose Regimen Modifications – Other Non-hematological Toxicity Events

| Grading | Dosing Modification Scheme | Recommended handling measures |
| --- | --- | --- |
| 1 | Original dose, administration continued | Continued observation, symptomatic treatment at the discretion of the investigator |
| 2-3 | 1. Judged by investigator Dose Delayed Until Recovered to Until tor Dose Delayed 2. Continue treatment at 1 to 2 dose levels lower or discontinue at the discretion of the investigator | Continuous observation, symptomatic treatment, repeated monitoring within 7 days |
| 4 | Treatment termination | Discontinued after symptomatic treatment |

5.4 Concomitant Medications and Treatments

During the study, except for the treatment measures specified in the study, other treatment measures (including concomitant medication, surgery, etc.) are all concomitant treatment. Investigators are required to record the contents of concomitant treatment in the original data and EDC in detail.

5.4.1 Concomitant Medications

5.4.1.1 Prohibited concomitant medications

The following therapeutic drugs are prohibited during the study. In case of any special circumstances beyond those specified in the protocol, the investigator should assess the rationality of medication, communicate with the sponsor to make a decision and make a detailed record.

- Drugs with antitumor effects

Systemic immunosuppressive agents, chemotherapeutic drugs, immunomodulators, modern traditional Chinese medicine preparations with anti-tumor indications.

- Systemic steroid hormone

Higher doses of systemic steroids tionsgator should assess the rationalitye.

5.4.1.2 Cautious concomitant use Drug

The following therapeutic drugs should be used with caution during the study. For special circumstances beyond those specified in the protocol, the investigator should assess the rationality of the medication, communicate with the sponsor for discussion and make a detailed record.

- Drugs that Interfere with Liver P450 Enzymes

Strong CYP3A inducers (rifampin, rifabutin, phenobarbital, phenytoin, carbamazepine, St. John's wort, etc.) and strong CYP3A inhibitors (ketoconazole, itraconazole, erythromycin, clarithromycin, etc.).

- Drugs that modulate P-gp

Strong inhibitors of P-GP (verapamil, cyclosporine A, dexverapamil, diaspora, bilicodar, etc.) and strong inducers of P-GP (rifampicin, etc.).

- Drugs causing cardiac QTc prolongation

It mainly includes but is not limited to the following types of drugs:

Antibacterials (clarithromycin, streptomycin, erythromycin, roxithromycin, metronidazole, moxifloxacin);

Antiarrhythmics (quinidine, sotalol, amiodarone, propylamine, procainamide);

Antifungals (fluconazole, ketoconazole);

Antiemetics (domperidone);

Antimalarials (mefloquine, chloroquine).

5.4.1.3 Permitted concomitant medications

Subjects may receive best supportive care or prophylactic measures such as prophylaxis for hyperglycemia, asthenia, decreased appetite, nausea, diarrhea, suspected opportunistic infection (PJP or CMV infection).

5.4.2 Concomitant Procedures

The risk of wound healing or bleeding with the study drug is unknown. Investigators should carefully assess the necessity of concomitant procedures. It is recommended that study drug be discontinued at least 2 days prior to surgery. After surgery, the time of retreatment is at the discretion of the investigator based on postoperative healing and recovery, but not less than 48 hours at the earliest.

5.4.3 Concomitant Medication and Treatment Collection Requirements

Table 13 Concomitant Medications and Treatment Collection Requirements

| Time period | RECORD REQUIREMENTS |
| --- | --- |
| From signing the ICF to before taking the first dose of study drug | All medications and significant non-drug therapies were recorded, including: generic drug name and daily dose; reason for drug therapy; start and end dates of drug therapy or whether drug was continued at study entry. |
| From the first dose to withdrawal from the trial | All drug treatments and significant non-drug treatments were recorded. |
| Withdrawn from the trial until 28 days after initiation of study drug or initiation of another treatment for the target indication (whichever occurred first) | Record all medications corresponding to the disposition of the AE. |
| Post-treatment period (after administration of 28 doses or initiation of other treatment for the target indication through the end of the trial) | Record the treatment associated with the target indication. |

6 STUDY ASSESSMENTS AND PROCEDURES

6.1 Study Procedures and Notes

Table 14 Study Procedures

| Study visits/assessments | Screening Period | Treatment period | | | | | | End of Treatment Visit | Follow-up after end of treatment | | | | |
| --- | --- | --- | --- | --- | --- | --- | --- | --- | --- | --- | --- | --- | --- |
|  |  | Cycle 1 | | Cycles 2-12 | | After Cycle 12 | | Study Completion/Early Withdrawal | **Safety follow-up #** | Non-PD Exit Follow-up | **Survival follow-up ¥** | |  |
|  | V0 | D14 | D28 | C2n  D28 | C2n + 1  D28 | C2nD28  (n D28 | **VEOT** | | **VS** | VPFS | **Vos** | |  |
| Window period | - 28-0 | ± 3 | ± 3 | ± 3 | ± 3 | ± 3 | - 28-+ 7 | | / | ± 7 | ± 7 | |  |
| Informed Consent | × |  |  |  |  |  |  | |  |  |  | |  |
| Demographic data | × |  |  |  |  |  |  | |  |  |  | |  |
| Past medical history | × |  |  |  |  |  |  | |  |  |  | |  |
| Concomitant diseases and abnormalities | × |  |  |  |  |  |  | |  |  |  | |  |
| Histopathological review ① | × |  |  |  |  |  |  | |  |  |  | |  |
| B symptom assessment | × |  |  |  |  |  |  | |  |  |  | |  |
| Height | × |  |  |  |  |  |  | |  |  |  | |  |
| Weight | × |  | × | × | × | × | × | |  |  |  | |  |
| Vital Signs | × | × | × | × | × | × | × | |  |  |  | |  |
| Physical examination | × |  | × | × | × | × | × | |  |  |  | |  |
| ECOG score | × |  |  | × |  | × | × | |  |  |  | |  |
| Epidemiology | × |  |  |  |  |  |  | |  |  |  | |  |
| Pregnancy test | × |  |  |  |  |  |  | |  |  |  | |  |
| Blood routine | × | × | × | × | × | × | × | |  |  |  | |  |
| Blood biochemistry | × | × | × | × | × | × | × | |  |  |  | |  |
| Glycosylated hemoglobin | × |  | × | × | × | × | × | |  |  |  | |  |
| Infection and immune indicator examination | × |  | × | × | × | × | × | |  |  |  | |  |
| CMV-DNA test | × |  | × | × | × | × | × | |  |  |  | |  |
| Urine routine | × | × | × | × | × | × | × | |  |  |  | |  |
| Stool routine | × |  |  | × |  | × | × | |  |  |  | |  |
| Amylase | × |  |  | × |  | × | × | |  |  |  | |  |
| Lipase | × |  |  | × |  | × | × | |  |  |  | |  |
| Coagulation function | × |  |  | × |  | × | × | |  |  |  | |  |
| Β2 microglobulin | × |  |  | × |  | × | × | |  |  |  | |  |
| Finger oxygen monitoring | × |  | × | × | × | × | × | |  |  |  | |  |
| Blood gas analysis | × | Add when there are symptoms such as (suspected) respiratory tract infection/pneumonia/ketoacidosis | | | | | | |  |  |  | |  |
| Pulmonary function monitoring | × |  |  |  |  |  |  | |  |  |  | |  |
| 12-Electrocardiogram | × |  | × | × | × | × | × | |  |  |  | |  |
| Echocardiogram | × |  |  |  |  |  |  | |  |  |  | |  |
| CT/MRI ② | × |  |  | ★ |  | ★ | ★ | |  | ★ |  | |  |
| PET-CT ② | × |  |  | ▲ | | | | |  | ▲ |  | |  |
| Bone marrow examination ③ | × |  |  | Add when CR is achieved | | | | |  |  |  | |  |
| Adverse events |  | × | | | | | | | |  |  | | |
| Concomitant Medications/Treatments | × | × | | | | | | | | | | | |
| Home blood glucose monitoring ④ |  | × | | | | | | | |  |  | | |
| Home urine ketone monitoring ④ |  | × | | | | | | | |  |  | | |
| Biological sample collection ⑤ | ■i | | | | | | | | | | |  | |

1. Visit Nomenclature

Screening: V0; Treatment: Cn, n represents number of cycles, 2n represents even cycles, 2n + 1 represents odd cycles.

End of treatment visit: VEOT; safety follow-up: Vs; follow-up for non-PD patients: VPFS; survival follow-up: Vos.

1. Frequency of Scheduled Visits

Day 14 and 28 visits of Cycle 1; Cycles 2-12: final (28-day) visit every week;

After Cycle 12: End of every 2 cycle (56 days) visit, ie, C14D28, C16D28, C18D28, C20D28, , (56 days) vi

1. General Principles of Visits

- Screening visit: It should be performed after the informed consent form is signed but before the first dose. For ethical reasons, the examinations that comply with the study requirements that have been performed before the subject signs the informed consent form may not be repeated.
- Treatment Period Visits: The next scheduled visit date is scheduled using the first dose of each cycle as the node.
- End of Treatment Visit (VEOT, defined as the date on which the investigator confirms that a subject requires discontinuation of study treatment for any reason):

If PET-CT within 56 days prior to VEOT, CT/MRI within 28 days prior to VEOT, cardiac function within 14 days prior to VEOT, and the remainder 7 days prior to VEOT, they do not need to be repeated at VEOT.

- Follow-up after end of treatment:

#: completed within 28 days of initiation, see Protocol "9 Safety Assessment" for safety event requirements.

¥: Survival follow-up will be performed by phone, outpatient or inpatient every 12 weeks with drug administration as the endpoint, including survival status, and other anti-tumor treatment received after the end of study (including treatment regimen, start and stop time, cycle, best efficacy, and reasons for treatment termination).

1. Special Instructions

**① Histopathological review: All subjects must provide pathological sections (stained sections and/or white films) and send them to the central pathology laboratory for pathological review (which can be freshly collected pathological tissue samples, or samples within 2 years before signing the informed consent form). If the archival tissue is not within 2 years prior to signing the informed consent and the subject is deemed unable to be re-collected based on clinical judgment, the site must communicate with the central laboratory to confirm whether the archival tissue is suitable for pathological diagnosis, or freshly collected prior to enrollment. For the specific process of centralized pathology consultation, please refer to the SOP.**

**② Imaging examination: including CT/MRI (symbol) and PET-CT (symbol ▲). See "6.2.4 Imaging examination" in the protocol for details.**

**③ Bone marrow examination: Including bone marrow aspiration and/or biopsy, which must be performed during the screening period. If it has been performed within 60 days before the first dose, it can be used as the screening examination result. For subjects with evidence of bone marrow involvement at baseline, if CR is assessed by imaging during treatment, bone marrow examination is required to confirm the efficacy (this should be completed 2 times after imaging).**

**④ Home blood glucose/urine ketone monitoring: It is noted to monitor blood glucose on C1D1, C1D3, C1D7, C1D10 and C1D14, at least twice a day, including fasting before breakfast and 2h after dinner (± 1h). Urine ketone monitoring is required while monitoring blood glucose, once a day. For subjects with fasting blood glucose > 8.9 mmol/L (2 or more consecutive times), home blood glucose monitoring (fasting before breakfast, 2h after dinner) should be performed continuously (at the specific frequency recommended by the investigator) during treatment.**

**⑤ Collection of biological samples (symbol): The subjects should provide samples during the screening period and at the time of disease progression (VEOT for non-PD subjects). At the same time, subjects may optionally provide tumor tissue samples for biomarker assessment during the screening period. See "8 Biomarker Assessment" in the study protocol for specific requirements and purposes.**

6.2 Visit content and requirements

6.2.1 Baseline characteristics of subjects

6.2.1.1 Demographic data and past medical history

Prior history and demographic data of the subjects should be collected as far as possible before enrollment, confirmed to meet inclusion/exclusion criteria, and the omitted or corrected prior history and demographic data found in the whole study process should also be recorded in the subsequent original data.

1. Demographic data: date of birth, gender, ethnicity and occupation;
2. Height, weight and ECOG score (see Appendix II);
3. Previous history:

- Histopathological diagnosis: pathological diagnosis, date of first diagnosis, pathological tissue acquisition method, histopathological grade;
- Clinical diagnosis before enrollment: Lugano stage (see Annex V), Follicular Lymphoma International Prognostic Index 2 (FLIPI-2) (see Annex VI);
- History of surgical treatment: date, surgical indications, description of surgical treatment;
- History of stem cell transplantation: date, type of transplantation, source of stem cells, description of transplantation, whether recurrence or progression and its date;
- History of chemical/targeted/immunotherapy (including adjuvant therapy): name of drug used, dose, best response, medication cycle, start and end time, whether treatment failure or progression and its date;
- History of radiotherapy: start and stop time, radiotherapy site, total dose, best efficacy.

1. Other past medical history:

- Disease history: Disease history within 5 years prior to signing ICF, such as diabetes and hypertension, should be recorded;
- Allergic history: allergen;
- Smoking history;
- Concomitant disease And abnormalities : Clinically significant abnormalities that are ongoing at Screening will be considered concurrent medical conditions or symptoms and will be observed continuously during the study after Investigator's judgment/diagnosis.

6.2.1.2 Vital Signs

Baseline vital signs should be completed within 3 days prior to the first dose. Pulse rate, blood pressure, body temperature and respiration. All vital signs should be measured with the subject at rest. If body temperature is measured more than twice in a day, the highest measurement should be recorded.

6.2.1.3 Physical examination

Complete physical examination at baseline should be completed within 7 days prior to the first dose. Including: general appearance examination, skin system, head (head, eyes, ears, nose and oral cavity), neck, lymph nodes, chest (lungs, heart, etc.), abdomen (liver and gallbladder, spleen, etc.), limb examination and nervous system examination. Targeted physical examinations were limited to systems that were clinically relevant (ie, cardiovascular, respiratory, lymph node, liver, and spleen) and associated with clinical signs/symptoms. In the event of clinical suspicion of disease progression at any time, a prompt physical examination will be required rather than waiting for the next scheduled imaging assessment.

6.2.1.4 B Symptom assessment

Baseline B symptom assessment should be completed within 7 days prior to the first dose. B symptom assessment content: unexplained weight loss of 10% (within 6 months before diagnosis), fever > 38℃ and rule out other causes of fever, night sweats (heavy sweating at night, need to change clothes and bedding).

6.2.2 Laboratory Tests

The baseline laboratory test results (the results of infection and immune indicators test, CMV-DNA test, epidemiology, blood gas analysis and pulmonary function monitoring were acceptable) 7 months before the first dose were beyond the acceptable time range. The test should be performed again to meet the requirements of the clinical study.

Table 15 Contents of laboratory tests

| Inspection category | Inspection item |
| --- | --- |
| Blood routine | White blood cell count (WBC), neutrophil count (ANC), lymphocyte count (LYM), red blood cell count (RBC), hemoglobin (HB), platelet count (PLT) |
| Blood biochemistry | Liver function: total bilirubin (TBIL), direct bilirubin (DBIL), alanine aminotransferase (ALT), aspartate aminotransferase (AST), alkaline phosphatase (ALP), serum albumin (ALB), serum total protein (TP), γ-glutamyltransferase (GGT), lactate dehydrogenase (LDH);  Renal function: blood urea nitrogen (BUN)/urea (UREA), creatinine (Cr); fasting blood glucose (GLU); uric acid (UA);  Electrolytes: potassium (K), sodium (Na), chloride (Cl), calcium (Ca), magnesium (Mg),  Inorganic phosphorus (P);  Four items of blood lipids: total cholesterol (TC), triglyceride (TG), high-density lipoprotein (HDL), low-density lipoprotein (LDL) |
| Glycosylated hemoglobin | Glycosylated hemoglobin (HbAlc) |
| Infection and Immunity  Indicator inspection | Infection indicators: procalcitonin (PCT) and C-reactive protein (CRP)  Immune parameters: CD4 + T lymphocyte count, CD8 + T lymphocyte count, CD4 +/CD8 + ratio |
| CMV-DNA  Inspection | Serum CMV-DNA levels (checked by CMV PCR) |
| Urine routine | Urine protein (PRO), urine glucose (GLU), urine red blood cell (RBC), urine white blood cell (WBC), urine ketone (KET), urine pH (PH) |
| Stool routine | Fecal white blood cells (WBC), fecal red blood cells (RBC), fecal occult blood (OB) |
| Β2 microglobulin | Β2 microglobulin (β2-MG) |
| Amylase | Serum Amylase (AMS) |
| Lipase | Serum Lipase (LPS) |
| Coagulation function | International normalized ratio (INR), prothrombin time (PT), activated partial thromboplastin time (APTT), thrombin time (TT), fibrinogen (FBG), D-dimer |
| Pregnancy test | Urine or serum pregnancy test, only for women of childbearing potential |
| Epidemiology | Anti-HIV ;  Hepatitis B surface antigen (HbsAg) (hepatitis B virus deoxyribonucleic acid HBV-DNA is added when positive), hepatitis B surface antibody (HBsAb), hepatitis B e antigen (HBeAg), hepatitis B e antibody (HBeAb) and hepatitis B core antibody (HBcAb);  Hepatitis C Antibody (Anti-HCV) |
| Finger oxygen monitoring | Saturation (SaO2) |
| Blood gas analysis | Saturation (SaO2), partial pressure of oxygen (PaO2), partial pressure of carbon dioxide (PaCO2), pH, oxygenation index (P/F) |
| Pulmonary function monitoring | Forced expiratory volume in the first second (absolute value of FEV1), pulmonary diffusion capacity (DLCO) |

Note: Laboratory tests specified in the protocol should be performed in our center if there are no special circumstances. If our center has no examination conditions, it is recommended to perform the test in a fixed outside hospital.

6.2.3 Cardiac function test

12-ECG: The ECG report and paper should be traceable 7 times before the first dose is administered during the screening period. The name of the subject and examination time should be clearly recorded by the thermosensitive spectrum, and the spectrum measured by the bedside ECG should be photocopied for reference only and not included in the statistical analysis.

Echocardiography: 14 results before the first dose are accepted in the screening period, and the examination results should include at least the score.

If chest pain, palpitation and other symptoms occur, it is recommended to additionally check echocardiography and myocardial enzymes (CK, CK-MB), troponin (cTnT, cTnI).

6.2.4 Imaging examination

**Examined site: The screening period must include brain, neck, chest, abdomen and pelvis; the subsequent cycles should include neck, chest, abdomen and pelvis at least. The sites with other suspicious lesions should also be examined as clinically indicated.**

**Examination methods: CT/MRI (CT is the first choice for neck, chest, abdomen and pelvis, MRI is the first choice for brain, and enhancement is required for all), whole-body PET-CT.**

**Requirements for examination: The examination during the treatment period should be performed under the same conditions as the screening period. For subjects enrolled without PD, PFS should be collected after enrollment, i.e., they should continue to return to the hospital for imaging examination as scheduled until PD (or initiation of other systemic anti-tumor treatment or death, whichever occurs first). The examination specification should refer to the 2014 edition of Lugano evaluation criteria to ensure that the efficacy can be reasonably assessed.**

**Time requirement: Imaging time does not change due to dose regimen modification. CT/MRI/PET-CT results meeting the study requirements 28 days before the first dose in the screening period.**

The specific time of CT/MRI examination:

① Screening period (within 28 days prior to the first dose);

② During treatment, the first dose is taken as the time point until disease progression (or initiation of other systemic anti-tumor therapy or death, whichever occurs first); CT/MRI assessment is performed every 8 weeks in the first 48 weeks ( time point until disease progression (or initiation of other systemic anti-tu

③ CT/MRI should be performed at the end of treatment visit (VEOT) regardless of confirmation of enrollment for any reason (if CT/MRI was performed 28 days before VEOT, it is not necessary to repeat at VEOT).

The specific time of PET-CT examination is:

① Screening period (within 28 days prior to the first dose);

② When CR was assessed by CT/MRI, complete remission (CR) was confirmed using PET-CT within 7 days after assessment;

③ PET-CT was performed on Day 112 (i.e., Week 16), Day 224 (i.e., Week 32), and Day 336 (i.e., Week 48) after the first dose (± 7 days), followed by PET-CT every 32 weeks (± 7 days), that is, at Weeks 80 and 112... PET-CT evaluation was performed at the same time point; when CT/MRI evaluation was CR and PET-CT confirmation was performed at 7 weeks, the latest 8 times of PET-CT examination plan could be exempted, and subsequent PET-CT was performed as planned normally (for example, when CT/MRI evaluation at Week 24 was CR and PET-CT confirmation was performed at 7 weeks, PET-CT at Week 32 was not performed);

④ PET-CT should be performed at the end of treatment visit (VEOT) regardless of confirmation of enrollment for any reason (if PET-CT was performed 56 days before VEOT, it is not necessary to repeat at VEOT).

**Record requirements: Radiographers should save the uncompressed images in DICOM format (film, JPG, BMP and other image formats are not acceptable) in the preferred media (CD/DVD) of the study site, and retain a copy of image data at the study site for review by regulatory authorities.**

7 EFFICACY ASSESSMENTS

The efficacy assessment of this study was performed by referring to the 2014 version of the Lugano Evaluation Criteria (Appendix VII), which was divided into investigator assessment at the study site and independent radiologic evaluation committee (IRC) assessment. Whether or not a subject continued treatment during the study was assessed by the authorized investigator at the study site.

For subjects who withdraw from the study prematurely and fail to obtain the primary efficacy endpoint, their primary endpoints should be followed up (unless the subject withdraws informed consent, or starts other anti-tumor treatment, or dies).

Further details regarding the independent central imaging assessment can be found in the "Central Imaging Operations Manual" provided by the sponsor.

8 Biomarker Assessments

Tumor tissue specimens and samples are planned to be collected in this study. Biological samples will be uniformly transported to the designated central laboratory for ctDNA-based detection using the second-generation sequencing (NGS) method to evaluate the biomarkers related to the efficacy, mechanism of action/resistance mechanism and safety of TQ-B3525. See the operation manual provided by the sponsor for the specific requirements for the collection, processing and transportation of biological samples.

1. Tumor tissue

Time requirement: Tumor tissue samples will be collected once during the screening period, either from the previous archive (within 2 years prior to signing the informed consent form) or freshly collected.

Requirements for samples: The provided tumor tissues should be formalin-fixed and paraffin-embedded and contain sufficient tumor tissues. If paraffin-embedded tumor tissue blocks are not available, 10 slides, each containing a lymph node or tissue section approximately 4-6 μm in thickness, may also be provided.

Note: Due to the difficulty in the collection of tumor tissue samples, the investigator may choose to collect tumor tissue samples after the subject signs the informed consent form according to the actual situation.

1. Epilepsy

8-10 ml at screening (within 7 days prior to first dose) and at disease progression (VEOT for non-PD rollover subjects).

Note: For sample collection, subjects who cannot be collected will not be eligible to participate in this study as necessary for the study.

9 SAFETY ASSESSMENTS

9.1 Adverse Events

An adverse event (AE) is any untoward medical occurrence in a clinical investigation subject administered a pharmaceutical product and which is manifested by symptoms, signs, disease, or laboratory abnormalities and which does not necessarily have a causal relationship with this treatment.

Investigators should record in detail any adverse events occurring during the medication of subjects. Recording of adverse events includes: description of adverse events and all related symptoms, onset time, severity, duration, measures taken, final outcome and outcome.

9.2 Evaluation of Adverse Events

The nature and severity of adverse events were evaluated according to the National Cancer Institute Common Terminology Criteria for Adverse Events [NCI-CTC AE 5.0].

9.3 Adverse events of records

During the AE reporting period stipulated in the protocol, the investigator is responsible for collecting all AEs (including SAEs) and recording them in the eCRF. When recording AEs, the investigator should use correct and standardized medical terminology to avoid colloquialisms and abbreviations. The start time of AE, the highest degree of NCI-CTC AE 5.0 grade, the end time, the correlation with the study drug, the influence with the study, the presence or absence of concomitant treatment and the recovery should be recorded.

Diagnosis vs Symptoms and Signs

If a diagnosis is already present, the diagnosis should be recorded in the eCRF rather than individual signs and symptoms (e.g., record liver failure rather than jaundice, elevated transaminases, and flapping tremor). However, if signs and symptoms cannot be attributed to a single diagnosis at the time of reporting, each individual event should be recorded as an AE on the eCRF. If a diagnosis is later established, it should be updated on the eCRF to record the diagnosis.

Adverse Events Secondary to Other Events

In general, AEs secondary to other events (e.g., caused by other events or clinical sequelae) should have their primary event recorded, unless the secondary event is more severe or an SAE. However, secondary events with significant clinical significance should be recorded as independent AEs in eCRF if they occur at a different time from the primary event; if the relationship between the events is unclear, the primary event and secondary event should be recorded separately.

Continuous, intermittent or isolated adverse events (frequency of adverse events)

Continuous AE refers to an AE persisting during the whole process without remission, for example, upper respiratory tract infection lasting for 5 days. Only one AE should be recorded in the eCRF. For severity assessment, the most severe severity throughout the event should be recorded.

Intermittent AEs are AEs that change or are relieved in symptoms, signs or laboratory parameters during the whole process, but do not have clinically significant outcomes, such as nausea and vomiting that last for many days, during which there is relative remission; subjects with hypertension have a relatively continuous course of hypertension despite intermittent remission in multiple blood pressure tests. Such AEs may only be recorded in one eCRF. The most severe severity over the course of the event should be recorded during the intensity assessment.

A separate AE (Single AE) is defined as an AE that can logically only occur alone, or that occurs only once during the trial, for example, an accident in which a subject falls while taking a drug; or vomiting that a subject only experiences once during the trial. Only one such AE should be recorded on the eCRF.

It should be noted that if the above AE has recovered from significant clinical significance, but the same AE occurs later and the latter is considered to have no continuous course with the former, the occurrence of the event should be recorded separately in the eCRF.

Laboratory abnormalities or vital signs

All laboratory test results may be recorded on the laboratory results page of the eCRF. Not all laboratory abnormalities/vital sign abnormalities should be recorded as AEs, and it is the responsibility of the investigator to review all laboratory abnormalities and abnormal vital signs and make medical judgment as to whether they should be recorded as AEs. Any of the above abnormalities with significant clinical significance, for example, one or more of the following conditions should be recorded as AE:

- Accompanying clinical symptoms
- Results in a change in study medication (e.g., dose modification, temporary or permanent discontinuation)
- Requires medical intervention or change in concomitant therapy (e.g., increase, suspension, discontinuation, or other change in concomitant medication, therapy, or treatment)
- Clinically significant as judged by the investigator

If a clinically significant laboratory abnormality or vital sign abnormality is a sign of a disease or syndrome (e.g., increased ALT/AST and blood bilirubin due to hepatic impairment), only the diagnosis (i.e., hepatic impairment) should be recorded on the Adverse Event form of the eCRF. Otherwise, laboratory abnormalities or vital sign abnormalities should be recorded on the Adverse Event form of the eCRF and indicate whether the test value is above or below the normal range. If there are standard clinical terms corresponding to the laboratory abnormalities or vital sign abnormalities, the clinical term should be recorded in the eCRF (e.g., increased blood potassium to 7.0 mmol/L should be recorded as"hyperkalemia").

Death

When recording a death event, if there is an AE leading to death, the AE leading to death should be recorded in the eCRF as an SAE using a single medical concept and the event should be reported as an SAE; if the cause of death is unknown, "unexplained death" should be recorded in the AE form of the eCRF and reported as an SAE as "unexplained death", followed by further investigation into the exact cause of death, and the record and SAE report should be updated after the cause of death is known.

Pre-existing medical conditions

The preexisting symptoms/signs a subject has already presented during the screening period of the trial will be recorded as AEs only when there is worsening in severity, frequency, or nature (except for worsening of the medical condition under study) after entry into the trial. Changes from the previous state should be reflected in the records, such as "increased frequency of headache", "aggravated hypertension", etc.

Hospitalization, prolonged hospitalization

The following situations result in hospitalization or prolonged hospitalization, which should not be reported as SAE:

- Planned hospitalization or prolongation of hospitalization as required by the protocol (e.g., for dosing, efficacy assessment, etc.);
- Hospitalization for a medical condition that was present and unchanged prior to study participation, such as an elective surgery or treatment scheduled before study participation, or an inpatient hospitalization for surgery or treatment scheduled after study participation, is not considered an adverse event. However, if the condition of the existing disease worsens during the study (e.g., surgery or treatment should be performed earlier than originally planned), hospitalization for surgery or treatment is required due to the deterioration of the disease, and the deterioration of the condition will be considered as an SAE.

Surgery

If the condition being treated by the procedure is known, the condition should be recorded as the AE rather than the procedure itself (e.g., if the subject has undergone inguinal hernia repair, an"inguinal hernia"should be recorded instead of"inguinal hernia repair"); however, if the reason for the procedure is unclear, the procedure may be recorded as the AE (e.g., if the subject has undergone abdominal exploration, an"abdominal exploration"may be recorded as the AE).

Pregnancy

If a female subject or female partner of a male subject becomes pregnant during the study, the investigator should be notified immediately. The investigator should report the completed pregnancy report form to the sponsor according to the SAE reporting procedure within 24 hours after learning of the pregnancy. Female subjects who become pregnant should discontinue study drug immediately. The investigator should discuss with the subject about the risks of continued pregnancy and possible effects on the fetus. Subjects should be monitored until the end of pregnancy. Pregnancies that occur within 28 days of study drug administration should be reported to the investigator.

Whether artificial or spontaneous abortion should be recorded and reported as SAE. Any congenital abnormality or birth defect of the infant born by female subject or female partner of male subject who has taken study drug should be recorded and reported as SAE.

Disease progression

An AE was to be considered if the occurrence of an event was clearly consistent with the expected pattern of progression of the primary tumor. Hospitalization solely due to progression of this disease is also considered an SAE. If symptoms cannot be confirmed to be completely caused by disease progression, or it is inconsistent with the expected pattern of tumor progression, relevant clinical symptoms may be recorded as AE, which conforms to SAE reportable SAE.

9.4 Follow-up of Adverse Events

The Investigator should follow all AEs until occurrence of any of the following:

- AE is relieved or improved to baseline level;
- Investigator confirms no further improvement is expected;
- Subject died;
- Subject lost to follow-up;
- The investigator confirmed that the AE was not related to the study treatment;
- Subject starts a new anticancer therapy;
- No clinical or safety data will be collected, or the database will be finally closed.

The final outcome of each AE, including date of AE resolution or death, must be recorded on the eCRF.

9.5 Criteria for judging the correlation between drugs and adverse events

The investigator should assess the possible association between adverse events and the study drug. See the following 5 criteria for judgment.

1. Whether there is a reasonable sequential relationship between the time of medication initiation and the time of occurrence of adverse reactions;
2. Whether the suspected adverse reactions comply with the types of known adverse reactions of the drug;
3. Whether the suspected adverse reactions can be explained by the effect of concomitant medication, the patient's clinical status or the effect of other therapies;
4. Whether the reaction is relieved or disappeared after drug withdrawal or dose reduction;
5. Whether the same reaction occurs again after re-exposure to the suspected drug.

Table 16 Criteria for determining the correlation between drug and adverse event

|  | 1 | 2 | 3 | 4 | 5 |
| --- | --- | --- | --- | --- | --- |
| Definitely related | + | + | - | + | + |
| Probably related | + | + | - | + | ? |
| Possibly related | + | + | ± | ± | ? |
| Unlikely related | + | - | ± | ± | ? |
| Definitely unrelated | - | - | + | - | - |

Note: + indicates yes, — indicates no, ± indicates difficult to confirm or deny, ? indicates that the situation is unknown

The three items judged as definitely related, probably related, and possibly related to the results were counted as adverse reactions, and the incidence of adverse reactions was calculated accordingly.

9.6 Serious Adverse Events

Any untoward medical occurrence that results in death, is life-threatening, results in persistent or significant disability or incapacity, requires inpatient hospitalization or prolongation of existing hospitalization, or is a congenital anomaly or birth defect after the subject has received investigational product. If a patient or his/her spouse becomes pregnant, it shall be reported to the relevant unit as a serious adverse event.

Disease progression, including signs and symptoms of progression, should not be reported as a serious adverse event, but death due to disease progression should be reported as a serious adverse event if it occurs during the trial or safety reporting period. Hospitalization for signs and symptoms of disease progression should not be reported as a serious adverse event. During the trial or safety reporting period, if the final outcome of cancer is death, the event that led to death must be reported as a serious adverse event.

9.7 Handling of Serious Adverse Events

For any serious adverse event occurred during the clinical trial, the investigator should report to the designated mailbox of PV Department of the sponsor in written form within 24 hours after being informed ( TQB3525@cttq.com ), followed by detailed and written follow-up reports in a timely manner. For the reporting of death events, the investigator should provide the sponsor and the Ethics Committee with other required data, such as autopsy report and final medical report. Upon receipt of a serious adverse event, the sponsor shall analyze and evaluate it immediately, including the seriousness, correlation with the investigational drug and whether it is an expected event. For suspected and unexpected serious adverse reactions, the sponsor should report them to all investigators participating in the clinical trial of investigational drug, clinical trial institutions and ethics committees in an expedited manner; and the investigator should report suspected and unexpected serious adverse reactions provided by the sponsor to the ethics committee. Suspected and unexpected serious adverse reactions should also be reported by the sponsor to drug regulatory authorities and health authorities.

9.8 Unexpected Serious Adverse Events Definitely Related/Suspected to the Study Drug Reaction

Suspected Unexpected Serious Adverse Reactions (SUSARs) that are definitely related/suspected to the study drug during this study should be subject to the Expedited Reporting Standards and Procedures for Safety Data during Clinical Trials issued by the Center for Drug Evaluation on April 27, 2018, which stipulates that the sponsor will perform the expedited reporting of SUSARs during the study, and the investigator is obliged to cooperate with the sponsor in the collection and discussion of relevant data.

9.9 Recommendations for Symptomatic Treatment of Common Adverse Reactions

Based on the characteristics of TQ-B3525, it is presumed that some of the following drug-related adverse events may occur, either immediately after the first dose or several weeks after the dose. When an AE occurs, the subject should receive supportive care or discontinue study drug as deemed necessary by the investigator. Recommendations for the treatment or management of some possible adverse events are listed below. For each adverse event, attempts should be made to rule out other causes, such as metastatic disease or bacterial or viral infections, which may require additional supportive care. The following treatment guidelines will be referenced in the event that the investigator determines the event is related to TQ-B3525. The following treatment recommendations may not be followed by the investigator if, after assessment, the adverse event is determined to be unrelated to the trial drug.

1. Hyperglycaemia

Antitumor targeted drugs that inhibit the PI3K/AKT/mTOR signaling pathway may cause hyperglycemia, and TQ-B3525 has been found to produce adverse effects of elevated blood glucose in some populations in previous clinical studies. Therefore, blood glucose levels should be closely monitored during study treatment, which may increase the frequency of blood glucose monitoring while increasing the monitoring of urine ketones.

The subject should follow the dietary guidance recommendations given by the physician or use hypoglycemic drugs to control blood glucose level based on the investigator's judgment. If the following treatment measures are not well controlled, the dose of the investigational drug TQ-B3525 should be reduced or even interrupted, and a consultation with a diabetes specialist should be made.

Table 17 TQ-B3525 Management of Hyperglycaemia

| Change in fasting blood glucose (FBG) (mmol/L) | Suggestions on blood glucose control | Study Drug Dose Modification Regimen |
| --- | --- | --- |
| ULN y Drug Dose | 1. Appropriate hydration, attention to dietary regimen, and enhanced exercise. 2. Oral hypoglycemic agents were given for prophylaxis or treatment, and metformin was recommended. | Original dose, administration continued |
| 8.9 < FBG ose, ad | 1. Appropriate hydration, attention to dietary regimen, and enhanced exercise. 2. Oral hypoglycemic agents were given for prophylaxis or treatment. Regular use of metformin, DPP-4 inhibitors, TZDs, SGLT-2 inhibitors and other drugs is recommended. If FBG is persistently poorly controlled in the range of 8.9 ~ 13.9, or postprandial blood glucose persists in the range of 15 ~ 18, metformin combined with oral hypoglycemic agents by another mechanism mentioned above can be considered for treatment (preferably in combination with the advice of diabetes specialist), and insulin can be used when necessary. Prophylactic use of oral hypoglycemic agents may be considered during subsequent therapy. 3. Meanwhile, pay attention to the changes in urine ketone and other indicators. 4. Strengthen patient education and improve patient compliance. | The investigator can determine whether to delay the administration and maintain the original dose or reduce the dose by 1 level in combination with fasting and blood glucose levels |
| FBG > 13.9 Uncomplicated (duration < 3 days) | 1. Assess whether hydration status is normal, remind patients to hydrate more, pay attention to dietary regimen, and strengthen exercise. 2. Consultation with a diabetologist is recommended as soon as possible for treatment. 3. Give oral hypoglycemic agents (it is recommended to combine two oral drugs, preferably metformin and DPP-4 inhibitors or TZDs). If the control effect is poor, consider subcutaneous injection of rapid-acting/short-acting/conventional insulin. Prophylactic use of oral hypoglycemic agents may be considered during subsequent treatment. 4. If postprandial blood glucose persists in the range of 18 ~ 20, the above dual oral hypoglycemic regimen or insulin can be considered for treatment. 5. Closely monitor the changes of FBG, urine ketone and other indicators to prevent the occurrence of complications. | Continue treatment at the original dose or 1 dose level lower at the investigator's discretion |
| FBG > 13.9 Uncomplicated symptoms (duration dose leve | 1. Assess whether hydration status is normal, remind patients to hydrate more, pay attention to dietary regimen, and strengthen exercise. 2. It is recommended to consult a diabetologist as soon as possible and hospitalize if necessary. 3. Controlled by subcutaneous rapid-acting/short-acting insulin, or intravenous insulin over a short period of time, then switching to basal + mealtime insulin. Subsequently, oral hypoglycemic agents can be given for prevention or treatment (it is recommended to combine two oral drugs, and metformin and DPP-4 inhibitors or TZDs are preferred). 4. If postprandial blood glucose persists /short-acting insulin, or intravenous insulin over a short period of time, then switching to basal + mealti 5. Closely monitor the changes of FBG, urine ketone and other indicators to prevent complications. | Continue treatment at 1 to 2 dose levels lower at the discretion of the investigator |
| FBG > 13.9 with symptoms | 1. Hospitalization was recommended and a diabetes specialist was contacted as soon as possible for consultation. 2. Fluid replacement should be performed as soon as possible to correct disturbances in the hydration status, electrolyte environment, and acid-base status of the body and control and treat complications. Insulin (0.1 U · kg − 1 · h − 1) was also continuously infused intravenously. 3. Closely monitor the changes of FBG, urine ketone and other indicators, and adjust the insulin dosage according to the changes of blood glucose. | Treatment termination |

Notes:

1. FBG refers to fasting blood glucose, including fasting blood glucose measured by laboratory tests and fasting blood glucose monitored at home.

2. dietary regimen recommendations: for example, small meals, low carbohydrates, high fiber, balanced carbohydrate intake during the day, three small meals and two snacks rather than a large meal, exercise, etc.

3. When urine ketone is positive (+) or serum β-hydroxybutyrate is > 0.3 mmol/L, it is recommended to discontinue SGLT-2 inhibitors and switch to other drugs for hypoglycemic therapy to prevent complications such as ketoacidosis. When transaminases (ALT, AST) are greater than 3 times the upper limit of normal or greater than 3 times the baseline value (when the baseline value is abnormal), TZDs should be discontinued and switched to other drugs for hypoglycemic therapy.

4. DPP-4 inhibitors: sitagliptin, vildagliptin, salbutamol, alogliptin and reserpine.

5. TZDs: rosiglitazone, pyrazone, etc.

6. SGLT-2 inhibitors: dapagliflozin, canagliflozin, empagliflozin, etc. In order to guard against complications of SGLT-2 inhibitor use, the use of such hypoglycemic agents requires consultation with a diabetes specialist.

When the time from the disappearance of the current hyperglycemia to the start time of the next hyperglycemia is f SGLT-2 inhibitor use, the use of such hypoglycemic agents requires consultation wihe previous event, two consecutive events can be recorded as the same adverse event, and the degree is taken as the highest. The start time is defined as the time of the previous hyperglycemia, and the end time is defined as the last outcome time of the next hyperglycemia.

1. Diarrhea, enteritis, nausea, or vomiting

Before the subjects start to take the study drug orally, the investigators should inform the subjects of the possibility of diarrhea and the treatment measures for diarrhea in detail. Symptomatic treatment should be given for diarrhea, and close follow-up or observation should be performed (≤ 7 days). It is clinically recommended to start oral montmorillonite powder, 3 g/bag, 3 times/day, or loperamide on the day of diarrhea, with a recommended dose of 4 mg, followed by 2 mg every 2 - 4 hours until there is no episode of diarrhea within 12 hours, and the maximum daily dose does not exceed 16 mg.

For diarrhea or accompanying complications that cannot be relieved after preventive treatment or symptomatic treatment, the drug should be suspended, and immediate supportive treatment should be given (such as intravenous drip of fluid to maintain water and electrolyte balance, intravenous drip of multivitamins, and potassium supplement in case of hypokalemia). The possibility caused by concomitant medication (e.g., metformin), gastrointestinal infection (including CMV, Portunus difficile, etc.) or gastrointestinal inflammation (through endoscopy or tissue examination, etc.) should be comprehensively assessed.

For unresolved diarrhea or conditions accompanied by complications, try to rule out other causes, such as metastatic disease or bacterial/viral infection (including CMV), which may require additional supportive care. If infection is ruled out and colitis is suspected or cannot be ruled out, steroids may be considered (with reasons if outside the protocol-specified dosing range).

Subjects should be closely monitored for signs and symptoms of enteritis, colitis (such as diarrhea, abdominal pain or cramps, blood in stool or mucous stool, with or without fever), and bowel perforation (such as peritoneal signs and bowel obstruction).

For nausea and vomiting, symptomatic treatment should be given first, and close follow-up should be performed. For grade amps, blood in stool or mucous stool, with or without fever), and bowel perforation (such as peritoneal signs and bowel obstruction). i restoring the original dose after the adverse event recovers to below grade 2. If vomiting is close to administration time on that day, occurrence time of vomiting should be recorded in detail. However, no matter whether vomiting affects the absorption of the study drug or not, the study drug will be continued in the administration cycle according to the protocol without supplement or cycle adjustment.

1. Hepatic function abnormal

The investigator shall give symptomatic treatment or observation for protecting liver according to the subject and adverse event, and increase the frequency of blood biochemical examination according to clinical needs. If hepatic function abnormality ≥ Grade 2 persists after active treatment or observation (≤ 14 days), dose delay may be considered. Resume the original dose after the adverse event recovers to grade 1 or normal. For subjects with lymphoma involving the liver or biliary obstruction, if ALT/AST exceeds 2.5 × ULN at enrollment, close monitoring of liver function is required.

1. Pneumonia/lung infection

PI3K inhibitor-mediated lung injury is mainly manifested as pneumonitis, pneumonia, of which pneumonitis is mainly considered to be immune-mediated, and pneumonia includes opportunistic infections due to decreased immune function of patients.

The data show that patients taking PI3K inhibitors have an increased risk of opportunistic infections. Fatal cases in clinical trials have been found to be mainly Pneumocystis jirovecii pneumonia (PJP, commonly known as carinii) and CMV-related diseases (CMV-emia or end-organ damage, such as hepatitis, enteritis, pneumonia, and retinal damage).

Patients may be initiated on prophylaxis for Pneumocystis Yersinii Pneumonia (PJP) prior to or during the study at the discretion of the investigator based on the potential benefit/risk ratio, regardless of the presence or absence of a high-risk profile. Also monitor T lymphocyte counts during treatment and consider initiating PJP prophylaxis if CD4 + T lymphocyte counts are < 0.2 × 10^9^/L. Prophylactic drugs against kapneumonia, mainly trimethoprim-sulfamethoxazole (cotrimoxazole), atovaquone, dapsone, and pentamidine. Because of reports of cross-sensitivity between sulfonamides and dapsone, subjects with known or suspected hypersensitivity to sulfonamides may receive inhaled pentamidine or atovaquone for PJP prophylaxis.

PI3K inhibitor-associated pneumonitis may be mildly symptomatic initially and is poorly characterized. It is necessary to guard against cold-like symptoms such as fever, cough, fear of cold and arthralgia of four extremities, as well as shortness of breath, chest distress, chest pain, dyspnea, fatigue and diarrhea. Even if mild, it is also necessary to be vigilant and timely reexamine. Subjects with (suspected) pneumonia/lung infection should be monitored for pulmonary symptoms (high-resolution chest CT). Once symptoms of pneumonia/pulmonary infection occur or are suspected, such as persistent fever, mild persistent cough, 5% decrease in saturation, dyspnea on exertion, or interstitial infiltration, it is recommended to immediately suspend the use of study drug TQ-B3525 and conduct a comprehensive examination.

When symptoms such as (or suspected) pneumonia/infection occur, in addition to chest CT examination, blood routine/infection/immunity, CMV and saturation examination, the following etiological examinations can also be considered to distinguish different types of pneumonia and/or infection: bronchoalveolar lavage, fiberoptic bronchoscopic lung biopsy, sputum culture, blood culture, G/GM test, Mycobacterium tuberculosis examination, etc. A variety of special fungi/bacteria/viruses such as Yersinia may be identified by NGS submission of bronchoalveolar lavage fluid or peripheral blood. If grade 3 febrile neutropenia or grade 4 neutrophil count decrease occurs, blood culture may be considered to identify sepsis, etc.

Once pneumonia or pulmonary infection symptoms occur, regardless of grade, study treatment should be suspended, and appropriate anti-infective treatment or hormone therapy should be given according to whether it is pneumonia/interstitial pneumonia, and the possibility of viral infection (CMV) and Pneumocystis jirovecii pneumonia (PJP) should be considered.

Recommended treatment measures are as follows:

(1) For patients diagnosed or highly suspected of interstitial pneumonia, comprehensively assess their oxygenation status and determine whether to use appropriate doses of glucocorticoid therapy (such as 0.5 mg/kg ~ 1.0 mg/kg methylprednisolone), and pay attention to calcium and vitamin D supplementation, monitor blood glucose, and prevent gastrointestinal bleeding.

(2) For those diagnosed or highly suspected of pneumonia, immediate empirical antibiotic treatment is recommended, while the etiology is identified as much as possible and sensitive anti-infective drugs are selected based on microbiological examination results). If the investigator evaluates that the possible cause of infection may be Yersinia pneumocystis pneumonia (PJP), trimethoprim-sulfamethoxazole, fluconazole, caspofungin and other drugs can be selected for treatment; for CMV infection, antiviral drugs (more, more, etc.) can be used.

(3) Mechanical assisted ventilation is required when respiratory failure symptoms occur.

9.10 Adverse Events of Special Interest

TQ-B3525, as an investigational drug, was not exposed to a sample size sufficient to elucidate known adverse events of special interest, and therefore awareness of adverse events associated with this drug remains limited. Potential adverse events that, similar to any new chemical, are generally class-related. Similar PI3K inhibitor drugs have been reported to have hyperglycemia complications, pneumonia/respiratory tract infection, enteritis, rash and other cases. Therefore, throughout the study, investigators should pay special attention to the occurrence and early signs of the above adverse events. In the event of such an event, the investigator should actively discuss with the sponsor representative to confirm the continuation of the event and take active therapeutic measures.

10 Study Data Management

10.1 Data Entry

An electronic data capture system (EDC) was used for data collection and management in this trial. The investigator or clinical coordinator should timely, accurately, completely and normatively enter the source data information into EDC. Only the investigator or clinical coordinator trained in EDC operation is authorized to perform data entry.

10.2 Data Verification and Review

The clinical research associate (CRA) should timely check the EDC data against the original medical documents to ensure the data are accurate, complete, consistent and standardized. Clinical Data Administrator shall review EDC data according to the Data Verification Plan to ensure the data logic, completeness and standardization.

10.3 Data Cleaning

Data cleaning methods include edit check of EDC system, SAS program verification and manual verification. CRA or Clinical Data Administrator raised a query on the trial data found in EDC in a timely manner, and the Investigator or Clinical Coordinator answered the query or corrected the data in a timely manner. If the query was resolved, the query was closed. If the query still existed, it was required to raise a query for further confirmation, until the query was finally resolved.

10.4 Electronic Signatures

After data cleaning, the investigator finally confirmed the authenticity and accuracy of the entered data, and electronically signed the data in EDC. The investigator who performed the electronic signature was required to sign an Electronic Signature Statement prior to implementation stating that the electronic signature had the same legal effect as a written, handwritten signature.

10.5 Database Lock

All the trial data are entered, data verification, data review and query resolution are completed, electronic signature is completed, medical verification is completed, and after the data review report is finalized, a data review meeting is held to jointly confirm the relevant matters before locking. After the Clinical Data Manager confirms the completion of database lock list, perform database lock. In principle, the database will not be unlocked after locking.

10.6 Data Transfer

After the database is locked, the Clinical Data Manager exports the data for submission to the Statistical Analyst and exports the subject eCRFs to each site for archiving.

11 Statistical analysis management

11.1 Statistical Analysis Datasets

**Full Analysis Set (FAS): all subjects who used the drug at least once for efficacy analysis. In the FAS analysis, subjects who have no tumor assessment after baseline will be considered as non-response.**

FAS set in Stage I and Stage II will be divided independently. The FAS in Stage II was the primary analysis set and was used for all analyses of efficacy and baseline characteristics.

**Per-protocol Set (PPS): radiographic evaluators who have completed 2 or more cycles of treatment, have at least one post-baseline follow-up, and meet the study protocol, have good compliance, and have not used prohibited drugs during the trial.**

The PPS set will be partitioned only for subjects in Stage II and not for subjects in Stage I. The PPS will be used for the per protocol analysis of the primary efficacy endpoint and key secondary efficacy endpoints. Prior to database lock, the principal investigator, statistician and sponsor will jointly negotiate to determine the final per protocol set at the data review meeting.

**Safety Analysis Set (SS): All patients who received at least one dose of the investigational drug and had safety records after medication were included in the safety analysis set.**

The SS set will include all subjects who meet the definition in Stage I and Stage II. The SS data set was used for all safety analyses.

In this trial, the FAS was used for the analysis of baseline data while the FAS and PPS were used for the analysis of main efficacy indicators. The FAS conclusion was used as the criterion. The SS was used for the analysis of laboratory test data, adverse events and adverse reactions.

11.2 Statistical Analysis Plan

11.2.1 General Principles

This study is divided into two stages. The first stage is only for exploration and not included in the analysis of the second stage. Therefore, the first stage is mainly based on descriptive statistics, and only the main efficacy indicators of the second stage are statistically inferred. Baseline analysis and safety analysis will be summarized in two phases for description.

For continuous variables, the number of non-missing subjects, mean, standard deviation, median, minimum, and maximum will be presented. The number of decimal places for the minimum and maximum values will be consistent with that recorded in the database. The mean and median will be one more decimal place than the original data recorded in the database, and the standard deviation will be two more decimal places than the original data recorded in the database. All decimals up to 4 decimal places.

Categorical variables will be presented as frequency tables (frequencies and percentages). Percentages will be presented to 2 decimal places.

11.2.2 Hypothesis test and test level

The test hypothesis is as follows:

H0: ORR hypoth

H1: ORR > 40%.

Test level α = 0.025 one-sided.

Statistical inference of ORR was performed using the confidence interval method. ORR is a highly superior indicator. Exact binomial method based on F distribution is used to calculate its (1-2 α)% confidence interval (CI). If its lower limit is higher than the target value (40%), the investigational drug is considered to be effective.

11.3 Study Population

11.3.1 Case Distribution

The number and percentage of subjects screened, treated with at least one dose of study drug, and prematurely discontinued treatment were summarized by study phase and study site. Subjects who fail screening will be summarized by center and primary reason. Premature termination (intermediate) treatment cases will be described by center and main reason grouping.

The case distribution in each data set, including the summary of subject distribution in each analysis population and the summary of the reasons for excluding subjects in each analysis population. Subjects who were excluded from each analysis population were listed by site (including protocol violations and reasons for exclusion).

Subjects who prematurely discontinue treatment will be tabulated by site.

Provide a detailed list of reasons for screen failure. Subject disposition plots were provided.

11.3.2 Protocol Violations and Deviations

Important protocol deviations will result in exclusion of subjects from the PPS, including the inclusion and exclusion criteria affecting the efficacy assessment, trial implementation, subject management or subject assessment.

Based on the FAS, the number and percentage of subjects without protocol deviation, with at least one protocol deviation, and with at least one important protocol deviation will be summarized; the number and percentage of subjects with important protocol deviations will be summarized and described by center grouping, including the number and percentage of subjects without protocol deviation, with at least one protocol deviation, and the number and percentage of subjects with each category of protocol deviation. All important protocol deviations will be tabulated.

11.3.3 Baseline Descriptive Statistics

The baseline analysis was based on the full analysis set, which was divided into Stage I, Stage II, and pooled totals. Baseline analysis is mainly performed by descriptive analysis, including demographic data, past medical history, vital signs, B symptom assessment, laboratory tests, cardiac function tests, etc. The mean, standard deviation, median, quartile, minimum and maximum values of continuous variables and the frequency, frequency or constituent ratio of categorical variables are described.

11.4 Efficacy Analysis

The efficacy analysis was based on the FAS and PPS, divided into Stage I and Stage II, in which the statistical inference was based on the results of Stage II.

11.4.1 Primary Efficacy Measures

Objective response rate (ORR) assessed by IRC:

The percentage of objective response (PR + CR) of the total number of cases and its 95% CI will be calculated. The 95% CI for ORR was calculated based on the exact binomial method using the F-distribution.

11.4.2 Secondary Efficacy Measures

Investigator-assessed (ORR): The percentage of objective responses (PR + CR) among the total number of cases was calculated with 95% CI. The 95% CI for ORR was calculated based on the exact binomial method using the F-distribution.

Duration of response (DOR): PFS and its 95% CI were estimated using the Kaplan-Meier method, and survival curves were plotted. Only calculated for responders.

Progression-free survival (PFS): PFS was estimated using the Kaplan-Meier method and survival curves were plotted.

Disease control rate (DCR): The percentage of disease control cases (CR + PR + SD) to the total cases and its 95% CI will be calculated. The 95% CI for DCR was calculated based on the exact binomial method of the F-distribution.

Overall survival (OS): OS was estimated using the Kaplan-Meier method and survival curves were plotted.

11.4.3 Listing of Individual Efficacy Data

All efficacy indicator data, including IRC assessment and investigator assessment, will be tabulated at subject level (two lines per subject, one for IRC assessment and the other for investigator assessment). All efficacy indicator data, including IRC assessment and investigator assessment, will be tabulated by visit interval (multiple records for each subject).

11.5 Safety Analysis

The safety analysis was based on the SS set, which was divided into Stage I, Stage II, and pooled total.

11.5.1 Drug Exposure

Drug exposure will be described by mean, standard deviation, maximum, minimum and median.

Summarize the exposure of subjects to study drug treatment, the number of cycles completed by patients, dose adjustments during treatment, and the cumulative number of dose adjustments during treatment.

Statistical description was performed on the treatment time of the study drug, total dose of the study drug and daily average dose during the treatment period.

11.5.2 Adverse Events

Adverse event data, after coding according to the current version of MedDRA at the start of coding, were processed in the statistical analysis.

**Treatment Emergent Adverse Events (TEAE): TEAE is defined as an adverse event that appears after or worsens after the use of the study drug.**

Adverse events, treatment-emergent adverse events (TEAEs), Grade 3 and higher TEAEs, serious adverse events, TEAEs related to the study drug, SAEs related to the study drug, TEAEs leading to dose modification, permanent treatment discontinuation, trial discontinuation, and subject death were summarized by number, number, and incidence; adverse events were summarized by SOC and PT; and NCI-CTC AE 5.0 severity grades were calculated by SOC and PT (repeated occurrence of the same AE was analyzed by the most severe number of participants and incidence of severity).

Death, other serious adverse events and adverse events of special interest (AESI). Deaths were defined as all on-study deaths and deaths due to procedures initiated during the study. Other serious adverse events refer to serious adverse events other than death (including SAEs temporally related to or prior to death). AESIs include significant hematological and other laboratory abnormalities (other than those meeting the definition of serious) as well as any event that results in an intervention, including dose reduction, interruption or discontinuation, or significant other concomitant therapy, unless reported as a serious adverse event. The number, number and incidence of the above adverse events and those related to the study drug will be described by SOC and PT. The number, number and incidence of NCI-CTC AE 5.0 will be calculated by SOC and PT, respectively.

A list of subjects with adverse events and a list of subjects with SAEs will be provided.

11.5.3 Laboratory Tests

Continuity The indicators describe the baseline mean and standard deviation, mean and standard deviation at each visit , Max, Min, Median Meanwhile, the mean, standard deviation and 95% will be described for the change from baseline at each visit CI.

Laboratory parameters were classified as low, normal, or high according to the normal range, and the changes from normal or normal baseline to low postbaseline and from normal or low baseline to high postbaseline were described. Postbaseline low or high was calculated using the lowest or highest postbaseline observation.

Alanine aminotransferase (ALT), aspartate aminotransferase (AST), alkaline phosphatase (ALP), and total bilirubin (TBIL) were classified according to NCI-CTC AE 5.0 criteria and described using cross classification tables.

All laboratory parameters will be listed and abnormal values will be annotated.

11.5.4 Vital Signs

For vital signs, the mean, standard deviation, median, quartiles, minimum and maximum will be listed, and measurements and changes before and after treatment will be described.

11.5.5 Electrocardiogram

For continuous variables, the baseline mean and standard deviation, mean and standard deviation at each visit, maximum, minimum and median will be described. Meanwhile, mean, standard deviation and 95% CI will be described for changes from baseline at each visit.

The overall evaluation of ECG was classified as normal, abnormal without clinical significance, abnormal with clinical significance and not done, and the cases of clinically significant abnormalities at least once after administration from normal or abnormal without clinical significance changes at baseline were described. Changes in baseline from the last examination will also be described. A cross classification table was used to describe the results.

All ECG examinations were tabulated.

The proportion of subjects with "clinically significant abnormalities" among subjects with abnormal changes will be described, and whether the abnormality is clinically significant will be judged by the investigator. List the abnormalities for administration.

11.5.6 ECOG Score

ECOG scores will be summarized descriptively by frequency and percentage using cross classification tables.

11.5.7 Concomitant Medications

The use of concomitant medications during the study (including any changes in concomitant medications during the screening period, new concomitant medications after screening, and concomitant medications during the follow-up period) and the frequency of use of each medication were summarized.

ATC classification coding will be performed by WHO Drug Global. Frequency summary description will be performed based on SS population by anatomic primary family (ATC first layer coding) and therapeutic group (ATC second layer coding).

11.6 Analysis of Biomarker Assessment

Tumor mutation status at baseline and after disease progression, and tumor mutation rates at baseline and after disease progression were calculated and compared using McNemar's paired χ2 test.

For other genes related to tumors, the frequency, frequency or constituent ratio shall be described according to the description method of categorical variables.

11.7 Statistical Analysis Software

Chia Tai Tianqing Pharmaceutical Group Co., Ltd. was responsible for the data management of this trial, and the Department of Biostatistics, School of Public Health, Nanjing Medical University undertook the statistical analysis of the study. Statistical analysis was performed using SAS 9.4 software programming analysis.

12 STUDY MANAGEMENT

12.1 Information Disclosure

After obtaining the ethical approval letter from the leading site of the clinical study, the study will be reviewed in CDE Drug Clinical Trial Registration and Information Publicity Platform, ClinicalTrials. Gov for registration publicity.

12.2 Regulatory and Ethical Review and Approval

This clinical trial must comply with the Declaration of Helsinki (2013 Edition), Good Clinical Practice (GCP) promulgated by the China Food and Drug Administration (NMPA), and relevant regulations. The approval from the Ethics Committee of the leading site must be obtained before the trial can be initiated. During the clinical study, any amendments to the study protocol shall be reported to the Ethics Committee and filed.

The clinical investigators will follow all applicable rules and regulations to protect subjects. The informed consent form used during the informed consent process must be reviewed by the sponsor, approved by the ethics committee, and available for inspection.

12.3 Informed Consent

The clinical investigator must inform the subject that participation in the clinical trial is voluntary, that the subject has the right to withdraw from the trial at any stage of the trial at any time without discrimination or retaliation, and that his/her medical treatment and rights and interests are not affected, and that he/she can continue to receive other forms of treatment. The subjects must be informed that their personal data in the trial will be kept confidential. The subjects shall also be informed of the nature of clinical trial, trial objective, expected possible benefits and possible risks and inconveniences, other alternative treatment options and the rights and obligations of the subjects conforming to the provisions of Declaration of Helsinki, so as to give the subjects sufficient time to consider whether they are willing to participate in the trial and sign the ICF.

Prior to performing any protocol-required procedures, subjects must:

- Be informed of the study and all contents and terms of the informed consent form.
- Sufficient time was given to ask questions and consider participation.
- Voluntary consent to participate in the study.
- Signed and dated the Ethics Committee approved consent form.

If the informed consent form is updated or amended during the study, written informed consent will be obtained from subjects who continue to participate in the study using the updated/amended informed consent form.

12.4 Protocol Amendments

During the actual implementation of this clinical trial, if it is necessary to revise this protocol, the revised trial protocol should be submitted to the Ethics Committee for approval before implementation. Unless it is necessary to eliminate an apparent immediate hazard to the subjects, the investigators will not make any changes to the study without prior approval from the Ethics Committee and the Sponsor. Changes to the protocol to eliminate an apparent immediate hazard to subjects may be implemented immediately, but must be documented in the protocol amendment, reported to the Ethics Committee, and submitted to the appropriate regulatory authority within the required timelines.

If important new information involving the investigational drug is found, the informed consent form must be revised in writing and submitted to the ethics committee for approval before obtaining the consent of the subject again.

12.5 Study Protocol Deviations

The investigator should record and describe all protocol deviations, and should timely report all protocol deviations that may affect the safety of subjects and data integrity to the ethics committee and the sponsor according to the requirements of the ethics committee.

12.6 Study Documentation

The investigator must maintain accurate records to enable the conduct of the study to be fully documented, including but not limited to the protocol, protocol amendments, informed consent forms, and Ethics Committee and governmental approval documents.

12.7 Study Termination

1. serious safety problems identified by the investigator;

2. the efficacy is too poor to be necessary to continue the trial;

3. The protocol has major errors and it is difficult to evaluate the drug effect;

4. If the administrative authority cancels the trial, the trial may be stopped halfway.

12.8 End of Study

The study director shall summarize the results of trial statistical analysis in an objective and detailed manner, actively complete the summary report of clinical trial so as to meet the requirements of regulatory authorities for unified regulation of clinical review of new drugs, and complete the sub-site summary by all participating units.

12.9 Quality Control and Assurance

In order to ensure the quality of the trial, the clinical study plan was discussed and formulated by the sponsor and the investigator before the formal start of the trial. Conduct GCP training for relevant study personnel participating in the trial.

Each study site must manage study drug according to the protocol and SOPs, including receipt, storage, dispensing, recovery, and destruction, as applicable.

According to GCP guidelines, necessary steps should be taken during the design and implementation stage of the study to ensure that the data collected are accurate, consistent, complete and credible. All the observed results and abnormal findings in the clinical trial shall be timely verified and recorded to ensure the reliability of data. The instruments, equipment, reagents and standards used for various inspection items in the clinical trial shall have strict quality standards and ensure that they work in the normal state.

The investigator entered the information required by the protocol into the eCRF, and the monitor verified whether it was completed completely and accurately, and instructed the site staff to make necessary corrections and additions.

The drug regulatory authorities, the Ethics Committee, the monitor and/or auditor of the sponsor may systematically inspect the activities and documents related to the clinical trial to evaluate whether it is conducted in accordance with the trial protocol, SOP and relevant regulations, and whether the trial data are recorded in a timely, true, accurate and complete manner. The audit does not directly involve the personnel conducting the clinical trial.

13. Investigator's Division of the Sponsor

13.1 Sponsor

1. Provide the investigator with materials and other support, and explain the protocol and filling of various materials to the investigator before the clinical initiation;
2. Dispatch CRA to make regular visits;
3. The monitor should ensure that he/she can keep in contact with the investigator at any time by phone, fax and mail;
4. The monitor will supervise the investigator to carry out the clinical study in accordance with the approved protocol, check the distribution and recovery of investigational drugs according to relevant regulations, and ensure the consistency between the trial records in the clinical trial and the data in the original report.

13.2 Investigators

1. Have received training on GCP and this trial protocol, and have time to carry out this trial according to the study protocol;
2. Prior to enrollment, subjects shall be informed of the study-related information in detail, and consent shall be obtained from subjects and informed consent shall be signed;
3. The investigator is obliged to take necessary measures to ensure the safety of subjects; in case of any adverse reaction, the investigator should immediately deal with it and report to the principal investigator according to relevant regulations; and follow up the serious adverse reaction;
4. Carefully fill in the study medical records in a timely manner;
5. Actively cooperate with the CRA in regular visits;
6. Complete records of laboratory tests, clinical records, and the subject's original medical records were retained.

14 Publication and patent of study results

The study results are owned by Chia Tai Tianqing Pharmaceutical Group Co., Ltd. The investigator (study site) should obtain the consent of Chia Tai Tianqing Pharmaceutical Group Co., Ltd. before publishing the paper. Before the main study results are officially published, the investigator (study institution) should exchange the clinical trial results at the academic conference with the consent of Chia Tai Tianqing Pharmaceutical Group Co., Ltd.

The patent application for the technical solution (including but not limited to the selection of indications and dose) agreed in this project shall be exclusively owned by CHIA TAI TIANQING Pharmaceutical Group CO., LTD. In the process of clinical trials, clinical research institutions shall jointly apply for patents with CHIA TAI TIANQING Pharmaceutical Group CO., LTD for innovative achievements independently completed by the agreed clinical protocol. CHIA TAI TIANQING Pharmaceutical Group CO., LTD and its affiliates may manufacture, use, sell, sell, commercialize the products covered by the above patents or those obtained by patent methods. However, without the permission of either party, neither party may transfer or in any way permit a third party to exploit such patent.

15 REFERENCES

1. Shankland KR, Armitage JO, Hancock BW. Non-Hodgkin lymphoma. Lancet. 2012, 380 (9844): 848.
2. Smedbya, KE, et al. Epidemiology and etiology of mantle cell lymphoma and other non-Hodgkin lymphoma subtypes. Sem in Cancer Biol 2011; 21:293-298.
3. Armitage, James O. My Treatment Approach to Patients With Diffuse Large B-Cell Lymphoma. Mayo Clinic Proceedings, 2012, 87 (2): 161-171.
4. Chinese Society of Hematology, Professional Committee of Lymphoma, Chinese Anti-Cancer Association, Chinese Societyof Hematology, et al. Chinese Guidelines for the Diagnosis and Treatment of Follicular Lymphoma (2013 Edition). Chinese Journal of Hematology, 2013, 34 (9): 820-824.
5. Montoto S, Davies A J, Matthews J, et al. Risk and Clinical Implications of Transformation of Follicular Lymphoma to Diffuse Large B-Cell Lymphoma. Journal of Clinical Oncology, 2007, 25 (17): 2426-2433.
6. Li, Li-Juan, Chai Y, Guo, Xiao-Jia, et al. Effects of endoplasmic reticulum stress on autophagy and apoptosis of human leukemia cells via inhibition of the PI3K/AKT/mTOR signaling pathway. Molecular Medicine Reports, 2018, 17 (6).
7. Samuels, Y. High Frequency of Mutations of the PIK3CA Gene in Human Cancers. Science, 2004, 304 (5670): 554-554.
8. Yuan T L, Cantley L C. PI3K pathway alterations in cancer: variations on a theme. Oncogene, 2008, 27 (41): 5497-5510.
9. Kong D, Yamori T. Phosphatidylinositol 3-kinase inhibitors: promising drug candidates for cancer therapy. 2008, 99 (9): 0-0.
10. Gopal AK, Kahl BS, de Vos S, et al. PI3Kδ inhibition by idelalisib in patients with relapsed indolent lymphoma. N Engl J Med. 2014 Mar 13; 370 (11): 1008-18.
11. Dreyling M, Santoro A, Mollica L, et al. Phosphatidylinositol 3-Kinase Inhibition by Copanlisib in Relapsed or Refractory Indolent Lymphoma. Journal of Clinical Oncology, 2017, 35 (35): 3898-3905.
12. Flinn I W, Miller C B, Ardeshna K M, et al. DYNAMO: A Phase II Study of Duvelisib (IPI-145) in Patients With Refractory Indolent Non-Hodgkin Lymphoma. Journal of Clinical Oncology, 2019, 37 (11): 912-922.
13. Alpelisib (ALP) + fulvestrant (FUL) for advanced breast cancer (ABC): results of the Phase 3 SOLAR-1 trial. 2018 ESMO.

Appendix I Calculation formula

1. Cockcroft-Gault creatinine clearance calculation formula

Ccr (mL/min) = [(140-age) × weight (kg)]/[72 × Scr (mg/dl)] or

Ccr (mL/min) = [(140-age) e) eight (kg)]/[72 × Scr (mg/Scr (umol/L)]

Note: Note that the unit of creatinine Scr is calculated, and it is calculated as 0.85 for female subjects

2. QTC calculation formula (Fridericia's formula)

QTcF = QT/(RR0.33), RR is the normalized heart rate value, obtained by dividing 60 by heart rate

Appendix II Eastern Cooperative Oncology Group Performance Status Scoring Criteria (ECOG score)

| 0 | Normal activity |
| --- | --- |
| 1 | Mild symptoms, self-care, able to engage in light physical activity |
| 2 | Can tolerate the symptoms of the tumor and take care of himself/herself, but can stay in bed for no more than 50% of the time during the day |
| 3 | The tumor symptoms are severe, with more than 50% of the time in bed during the day, but they can also get up and stand, and some of them take care of themselves |
| 4 | Critically ill Bedridden |
| 5 | Death |

Appendix III New York Heart Association (NYHA) Functional Classification

| Grading | New York Heart Association (NYHA) Class |
| --- | --- |
| Grade I | No limitation of physical activity, ordinary activity does not cause undue fatigue, dyspnea or palpitation. That is, the compensatory phase of cardiac function. |
| Grade II | Slight limitation of physical activity. Asymptomatic at rest, fatigue, palpitation, dyspnea, or angina may be precipitated by ordinary activity. It is also known as grade I or mild heart failure. |
| Grade III | Marked limitation of physical activity, absence of symptoms at rest, less than ordinary activity producing the above symptoms. Also known as grade II or moderate heart failure. |
| Grade IV | Inability to engage in any physical activity, congestive heart failure or angina symptoms at rest, aggravated by any physical activity. It is also known as grade III or severe heart failure. |

Cardiac function is divided into four classes, and heart failure is divided into three degrees (slightly supplemented according to NYHA classification).

Appendix IV Definition of Relapsed/Refractory Follicular Lymphoma

Patients were required to have been adequately treated with prior rituximab-containing regimens or to have disease progression on rituximab, and inclusion of subjects who did not meet this requirement should be justified (e.g., intolerant to rituximab).

- Relapse: defined as disease progression after response to adequate therapy with at least one regimen containing rituximab.
- Refractory to rituximab: defined as failure to respond to adequate treatment with a rituximab-containing regimen (combination chemotherapy or chemotherapy) or disease progression during treatment/within 6 months of the end of adequate treatment.

* "Adequate treatment with rituximab-containing regimen" refers to the completion of full cycle treatment with rituximab combined with chemotherapy according to the pathological type and disease stage requirements, or at least 4 weekly injections of 375 mg/m2 for rituximab treatment.

* "Progression on treatment" required that at least one cycle of rituximab plus chemotherapy or chemotherapy was completed if progression occurred during induction therapy and at least one dose of injection was completed if progression occurred during maintenance therapy.

* "Response" includes complete response and partial response.

* Reference of this standard: Center for Drug Evaluation (CDE), CFDA: Recommendation on the Definition of Population for Clinical Studies of Relapsed/Refractory CD20 + B-cell Non-Hodgkin Lymphoma:

Http://www.cde.org.cn/dzkw.do?method=largePage&id=314472, 2018-05-15.

Appendix V 2014 Lugano Staging Criteria

| Limited-stage | |
| --- | --- |
| Phase I | Invasion into only a single nodal region (I), or invasion into a single extranodal organ without nodal involvement (IE) |
| Phase II | Invasion thout nodal involvement (IE) on (I), or invasion into a single extranodalon on the Definition extranodal organ involvement in the ipsilateral nodal drainage area (IIE) (e.g., thyroid involvement with cervical nodal involvement, or mediastinal nodal involvement extending directly to the lung) |
| Stage II Large Mass * | Stage II with bulky disease |
| Progressive phase | |
| Phase 3 | Invasion into superior and inferior diaphragmatic lymph node regions, or invasion into supradiaphragmatic lymph nodes + splenic involvement (IIIS) |
| Stage IV | Invasion into extranodal organs outside the draining area of lymph nodes (IV) |
| Legend: CT, MRI or PET/CT as staging methods | |

Notes:

1. * According to the 2014 Lugano modified staging criteria, the specific data of bulky lesions of lymphoma are no longer limited, and it is only necessary to clearly record the maximum diameter of the largest lesion in the case; for patients with stage II large masses, the treatment principle should be selected as appropriate according to the pathological type and poor prognostic factors of the disease, and for patients with indolent lymphoma with large masses, the limited-stage treatment mode can be selected, but for patients with aggressive lymphoma with large masses, the advanced treatment mode should be selected.

2. Definition of lymph node distribution area

Supra-diaphragmatic (12 areas in total, one area for left and one area for right since they cannot be covered by a radiation field): Wechsler ring (a ring of lymphoid tissue for nasopharynx and oropharynx, including palatine tonsil, posterior pharyngeal adenoid, lingual tonsil and other lymphoid tissues at this site as an area), left/right neck (a region before unilateral ear, occipital region, submandibular region, submental region, internal carotid region and supraclavicular region), left/right subclavian region, left/right axillary fossa (including chest and internal mammary gland), left/right trochlear (including antecubital fossa), mediastinum (including paratracheal region and thymus region) and left/right hilum;

Subphrenic (9 areas total): spleen, upper abdomen (hilum of spleen, hilum of liver, abdominal cavity), lower abdomen (one area for para-aortic, retroperitoneum, perimesenteric, other unspecified abdominal lymph nodes), left/right para-iliac vessels, left/right groin (including thigh), left/right popliteal fossa.

3, tonsils, Wechsler's ring, and spleen were regarded as lymphoid organs.

Appendix VI Follicular Lymphoma International Prognostic Index 2 (FLIPI-2)

| Item | 0 point | 1 point |
| --- | --- | --- |
| Age | < 60 years | ≥ 60 years |
| Hemoglobin level | ≥emoglobin | < 120 g/L |
| Β2 microglobulin | Normal | Higher than normal |
| Bone marrow invasion | None | Yes |
| Maximum diameter of largest lymph node | < 6 cm | > 6 cm |

Appendix VII 2014 Lugano Evaluation Criteria

Note: Efficacy was evaluated using the criteria revised by the 2014 Lugano meeting and divided into radiographic response (CT/MRI assessment) and metabolic response (PET-CT assessment).

|  | Lesion area | PET-CT evaluation | CT evaluation |
| --- | --- | --- | --- |
| CR | Lymph node and extranodal involvement | 5PS score 1, 2, 3 * points, with or without residual lesions;  Note: Metabolism may be higher in Wechsler's ring, extranodal high metabolic uptake organs such as spleen or bone marrow stimulated by G-CSF than in mediastinum/liver blood pool. CR should be evaluated compared with background level | Target lesion (lymph node) long diameter (Ldi) i) t lesi |
|  |  |  | No extranodal disease |
|  | Non-measurable lesions | N/A | Vanishing |
|  | Organ enlargement | N/A | Back to normal |
|  | New lesions | None | None |
|  | Bone marrow | No evidence of bone marrow FDG-sensitive disease | Normal morphology, IHC negative if indeterminate |
| PR | Lymph node and extranodal involvement | 5PS score 4-5 points, with decreased uptake from baseline, residual lesions can be of any size | Sum of PPD (Ldi di of t axis perpendicular to Ldi) of up to 6 target lesions, i.e. e. uptake from baseline |
|  |  | Interim assessment, which suggests effective treatment | When the lesion is too small to measure: 5 mm reatmen |
|  |  | End stage assessment, the above conditions indicate residual disease | When the lesion disappears: 0 mm onditio |
|  | Non-measurable lesions | N/A | Disappeared/normal, residual lesion/no increase in lesion size |
|  | Organ enlargement | N/A | Decrease in the long diameter of the spleen > 50% of the increase in the original long diameter; the normal size of the spleen is usually 13 cm. If the original size is 15 cm, PR needs to be < 14 cm in the long diameter |
|  | New lesions | None | None |
|  | Bone marrow | Residual uptake above normal marrow tissue but decreasing from baseline; if nodular local abnormal changes persist in the marrow, MRI or biopsy or interim assessment is required for further diagnosis | N/A |
| SD | Target lesions (nodal/nodular masses, extranodal lesions) | No metabolic response: no significant change from baseline in the intermediate/terminal phase rating 5PS score of 4-5 | < 50% increase in SPD of up to 6 target lesions, without evidence of PD |
|  | Non-measurable lesions | N/A | Not reaching PD |
|  | Organ enlargement | N/A | Not reaching PD |
|  | New lesions | None | None |
|  | Bone marrow | Same as baseline | N/A |
| PD | Individual target lesions (nodal/nodular masses, extranodal lesions) | 5PS score 4-5 points with increased uptake compared with baseline, and/or new increased uptake at mid- or end-stage evaluation | Progression of at least 1 target lesion is diagnostic, while nodal/extranodal disease must also meet the following criteria:  Ldi > 1.5 cm  ≥> 1.5 cm cm also meet the following criteri  Increase in Ldi or Sdi from smallest status: 0.5 cm (m (cm (m (us: 0.5 cm (ria: le nodal/and/o |
|  |  |  | If the increase in the long diameter of the spleen is > 50% of the increase in the original long diameter, it often defaults to the normal size of the spleen of 13 cm. If the original size is 15 cm, it is judged that the long diameter of PD needs to be > 16 cm;  If there is no splenomegaly at baseline, the long diameter should be increased by at least 2 cm on the basis of baseline;  New or recurrent splenomegaly |
|  | Non-measurable lesions | None | New lesions or unequivocal progression of existing non-measurable lesions |
|  | New lesions | Occurrence of new hypermetabolic foci associated with lymphoma (excluding infection, inflammation, etc.), requiring biopsy or interim evaluation if not clear in nature | Re-enlargement of previously relieved lesions |
|  |  |  | New lymph nodes > 1.5 cm in any diameter |
|  |  |  | New extranodal lesions > 1.0 cm in any diameter, < 1.0 cm in diameter Demonstrate whether the lesion is lymphoma-related |
|  |  |  | Lesions of any size unequivocally associated with lymphoma |
|  | Bone marrow | New or recurrent high metabolic uptake | New or recurrent bone marrow involvement |

Deauville's PET rating 5-point scale:

1 point: Uptake rating 5-point

2 points: Uptake background; scale: nt te

3 points: Mediastinal blood pool < lesion uptake lymphoma onstrate w

4 points: Uptake > liver blood pool (mild);

5 points: Uptake > liver blood pool (significant, SUVmax > 2 times liver blood pool) or new lesions;

Score X: New abnormal uptake, which was considered to be unrelated to lymphoma;

* 5PS score of 3: good prognosis under standard treatment is indicated in most subjects, especially for subjects with interim evaluation. However, in some clinical trials of step-down therapy, a score of 3 is considered to be a poor treatment effect and undertreatment needs to be avoided.

Measurable lesions:

Up to 6 significant lymph node/nodal fusion masses, extranodal lesions, and both diameters easily measurable;

(1) Lymph nodes: Lymph nodes should be classified according to region. If there is mediastinal and retroperitoneal lymphadenopathy, these lesions should be included; measurable lymph nodes should be > 1.5 cm in long diameter;

(2) Non-nodal lesions: including solid organs (such as liver, spleen, kidney, lung, etc.), digestive tract, skin or palpable labeling part, measurable extranodal lesions need to be > 1.0 cm in long diameter.

Non-measurable lesions:

Any significant lesion that is not measurable/assessable is considered non-measurable. These include:

(1) Any nodal/nodal fusion mass, extranodal disease, i.e., any portion that is not selected as marked, or that is measurable, or that does not meet the criteria for measurability but is still considered a lesion;

(2) Consider disease involvement but difficult to quantify, such as pleural effusion, ascites, bone metastasis, leptomeningeal involvement, abdominal mass lesions;

(3) Other undiagnosed lesions requiring imaging follow-up;

Wechsler's ring and extranodal sites (e.g., digestive tract, liver, bone marrow): FDG uptake may be higher than mediastinal cisterns in CR, but higher than surrounding background levels (e.g., bone marrow is generally elevated due to chemotherapy or G-CSF metabolic activity).
