# Supplementary material for: Phase II study of novel orally PI3Kα/δ inhibitor TQ-B3525 in relapsed and/or refractory follicular lymphoma
Source: Signal Transduct Target Ther. 2024 Apr 17;9:99. doi: 10.1038/s41392-024-01798-0 (PMC11021411; doi:10.1038/s41392-024-01798-0)
Supplement: Supplementary file 2 — Sigtrans_Supplementary_Materials [file 41392_2024_1798_MOESM2_ESM.docx]

Supplementary Materials for

Phase II study of novel orally PI3Kα/δ inhibitor TQ-B3525 in relapsed and/or refractory follicular lymphoma

Huaqing Wang ^1,2,#^, Jifeng Feng ^3^, Yanyan Liu ^4^, Zhengzi Qian ^5^, Da Gao ^6^, Xuehong Ran ^7^, Hui Zhou ^8^, Lihong Liu ^9^, Binghua Wang ^10^, Meiyun Fang ^11^, Hebing Zhou ^12^, Zhenqian Huang ^13^, Shi Tao ^14^, Zhuowen Chen ^15^, Liping Su ^16^, Hang Su ^17^, Yu Yang ^18^, Xiaobao Xie ^19^, Huijing Wu ^20^, Ping Sun ^21^, Guoyu Hu ^22^, Aibin Liang ^23*^, Zhiming Li ^24*^

^1^ Department of Medical Oncology, Tianjin Union Medical Center of Nankai University, Tianjin, 300121, PR China;

^2^ The Institute of Translational Medicine, Tianjin Union Medical Center of Nankai University, Tianjin, 300121, PR China;

^3^ Department of Medical Oncology, Jiangsu Cancer Hospital, The Affiliated Cancer Hospital of Nanjing Medical University, Nanjing, 210009, PR China;

^4^ Department of Medical Oncology, Henan Cancer Hospital, The Affiliated Cancer Hospital of Zhengzhou University, Zhengzhou, 450003, PR China;

^5^ Department of Medical Oncology, Tianjin Medical University Cancer Institute and Hospital, Tianjin, 300060, PR China;

^6^ Department of Hematology, The Affiliated Hospital of Inner Mongolia Medical College, 010050, Hohhot, PR China;

^7^ Department of Hematology, Weifang People’s Hospital, The First Affiliated Hospital of Weifang Medical University, 261000, Weifang, PR China;

^8^ Department of Lymphoma & Hematology, Hunan Cancer Hospital, The Affiliated Cancer Hospital of Xiangya School of Medicine, Central South University, 410013, Changsha, PR China;

^9^ Department of Hematology, The Fourth Hospital of Hebei Medical University and Hebei Tumor Hospital, 050011, Shijiazhuang, PR China;

^10^ Department of Lymphoma, Weihai Central Hospital, 264400, Weihai, PR China;

^11^ Department of Hematology and Rheumatology, The Affiliated Zhongshan Hospital of Dalian University, 116001, Dalian, PR China;

^12^ Department of Hematology, Beijing Luhe Hospital, 101199, Beijing, PR China;

^13^ Department of Hematology, The First Affiliated Hospital of Guangzhou Medical University, 510120, Guangzhou, PR China;

^14^ Department of Hematology, The First Affiliated Hospital of Hainan Medical College, 570102, Haikou, PR China;

^15^ Department of Hematology, The First People’s Hospital of Foshan, 528000, Foshan, PR China;

^16^ Department of Hematology, Shanxi Cancer Hospital, 030013, Taiyuan, PR China;

^17^ Department of Lymphoma, Senior Department of Hematology, The Fifth Medical Center of Chinese PLA General Hospital, 100039, Beijing, PR China;

^18^ Department of Lymphoma and Head and Neck Cancer, Fujian Cancer Hospital, 350014, Fuzhou, PR China;

^19^ Department of Hematology, The First People’s Hospital of Changzhou, The Third Affiliated Hospital of Soochow University, 213003, Changzhou, PR China;

^20^ Department of Medical Oncology, Hubei Cancer Hospital Affiliated to Tongji Medical College, Huazhong University of Science and Technology, 430079, Wuhan, PR China;

^21^ Department of Medical Oncology, Yantai Yuhuangding Hospital, 264000, Yantai, PR China;

^22^ Department of Hematology, Zhuzhou Central Hospital, 412007, Zhuzhou, PR China;

^23^ Department of Hematology, Tongji Hospital of Tongji University, Shanghai, 200333, PR China;

^24^ Department of Medical Oncology, State Key Laboratory of Oncology in South China, Collaborative Innovation Center for Cancer Medicine, Guangdong Provincial Clinical Research Center for Cancer, Sun Yat-sen University Cancer Center, 510060, Guangzhou, PR China.

Correspondence to: Zhiming Li, lizhm@sysucc.org.cn, and Aibin Liang, Lab7182@tongji.edu.cn


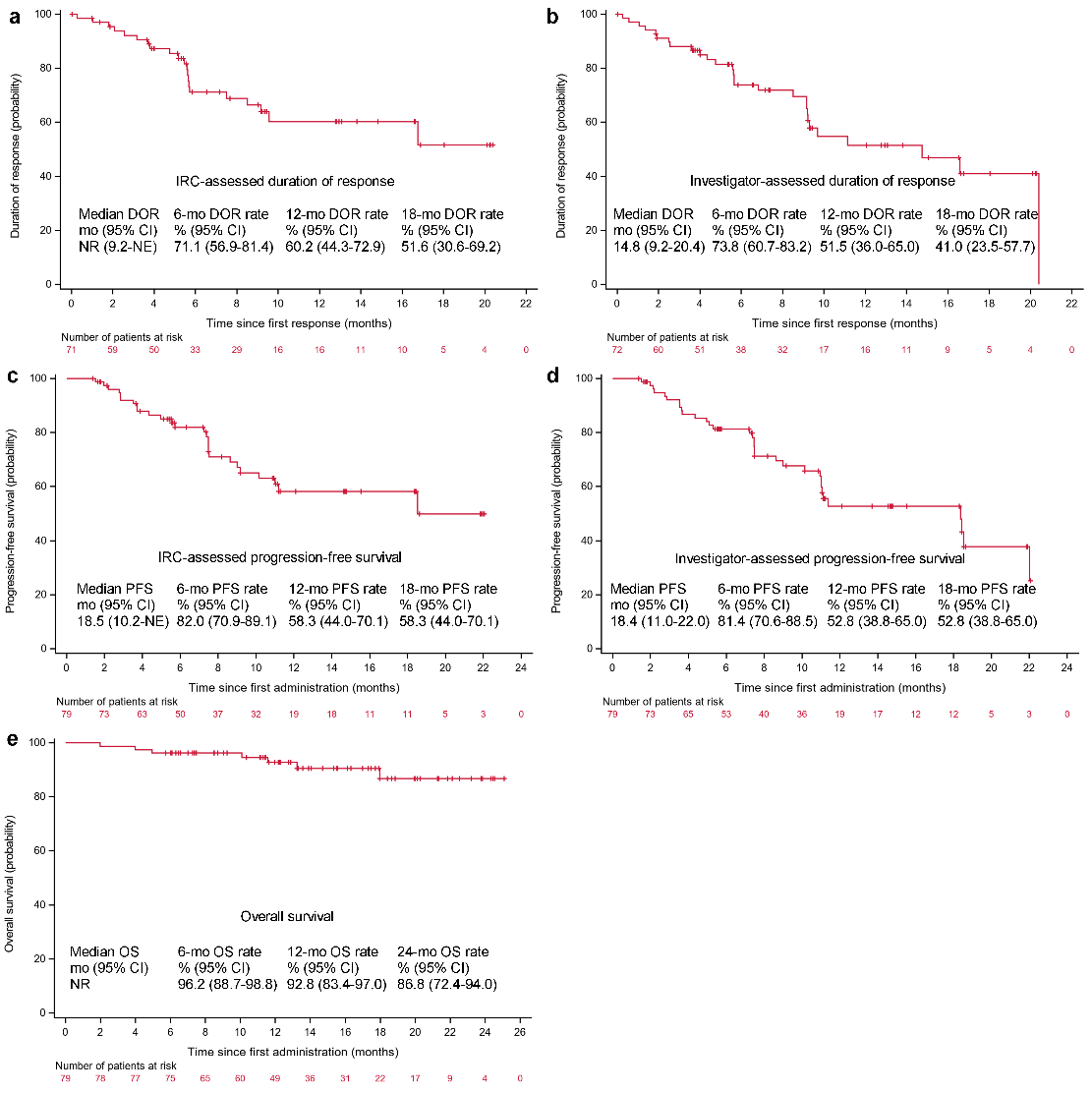


# Figure S1. Kaplan-Meier curves for DOR, PFS, and OS at stage 2 (per-protocol population).

DOR based on IRC assessment (a) and investigator assessment (b). PFS based on IRC assessment (c) and investigator assessment (d). OS (e).

**Abbreviations:** DOR, duration of response; PFS, progression-free survival; OS, overall survival; mo, months; CI, confidence interval; NR, not reached; NE not estimable; IRC, independent review committee.


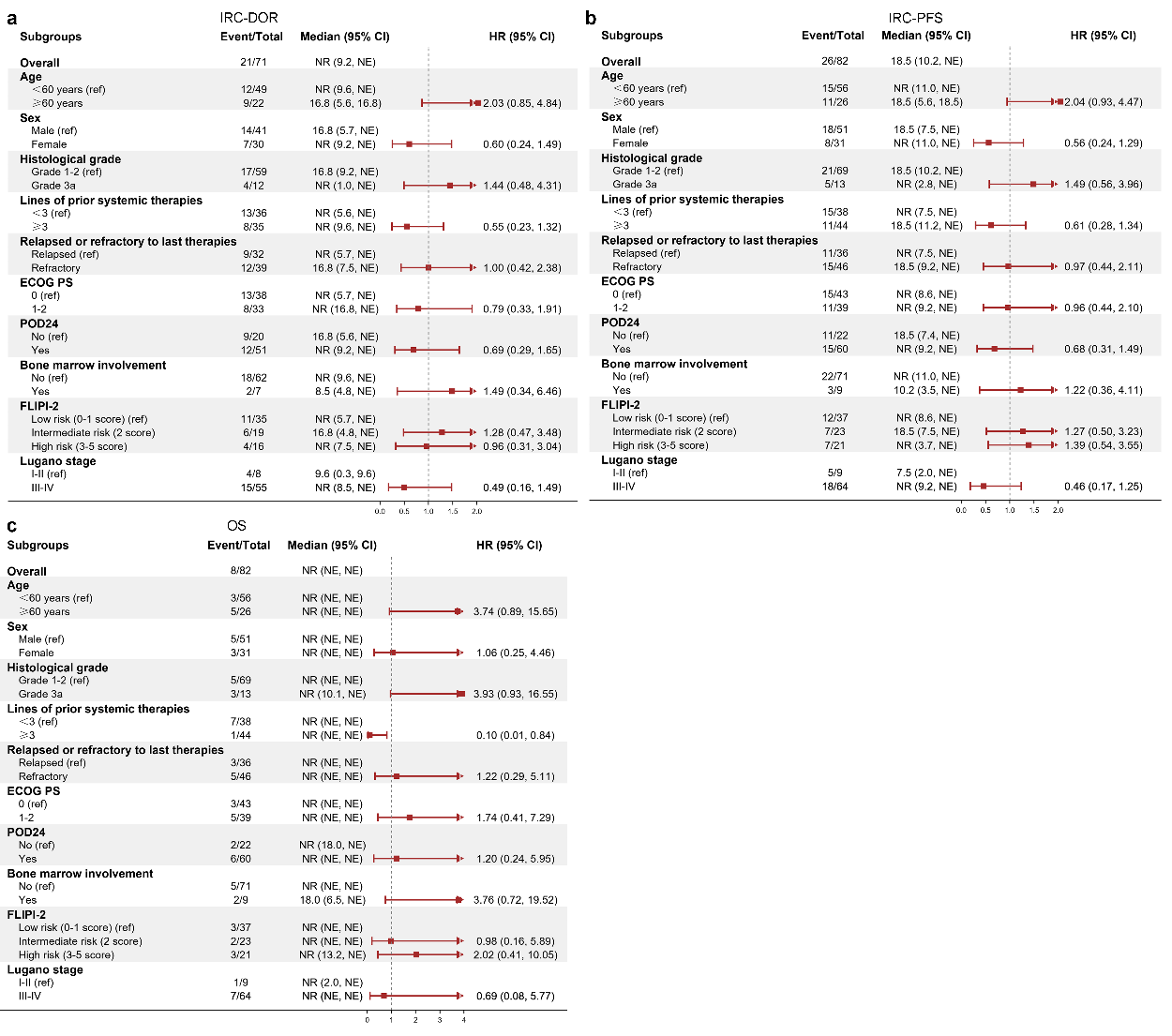


# Figure S2. Forest Plot of subgroup analysis for DOR, PFS, and OS in ITT at stage 2.

(a) IRC-assessed DOR. (b) IRC-assessed PFS. (c) OS.

**Abbreviations:** DOR, duration of response; PFS, progression-free survival; OS, overall survival; ITT, intent-to-treatment; CI, confidence interval; ECOG PS, Eastern Cooperative Oncology Group Performance Score; FLIPI-2, Follicular Lymphoma International Prognostic Index 2; POD24, progression of disease within 24 months; IRC, independent review committee; HR, hazard ratio; NR, nor reached; NE, not estimable.

Table S1. Studies evaluating treatment for follicular lymphoma and PI3K inhibitors.

| **Study** | **Target disease** | **Patient number** | **Regimens** | **Current therapy setting** | **Drug type** | **ORR** | **CRR** | **DOR, mo** | **PFS, mo** | **OS, mo** | **Ref.** |
| --- | --- | --- | --- | --- | --- | --- | --- | --- | --- | --- | --- |
| Phase II; Huaqing W et al | R/R FL | 82 | TQ-B3525 | Three or more lines | PI3Kα/δ inhibitor | 86.6% | 34.2% | NR | 18.5 | NR | - |
| Phase II; Dreyling M et al. | R/R indolent lymphoma (including R/R FL) | 142 (R/R FL 104) | Copanlisib | Three or more lines | PI3Kα/δ inhibitor | 59% (R/R FL 59%) | 12% (R/R FL 14%) | 22.6 (R/R FL 12.2) | 11.2 | NR | ^1^ |
| Phase II; Gopal AK et al. | R/R indolent lymphoma (including R/R FL) | 125 (R/R FL 72) | Idelalisib | Three or more lines | PI3Kα inhibitor | 56.8% | 5.6% | 12.5 | 11 | 20.3 | ^2^ |
| Phase II; Wang et al. | R/R FL | 114 | Linperlisib | Three or more lines | PI3Kδ inhibitor | 79.8% | 15.5% | 12.3 | 13.4 | NR | ^3^ |
| Phase II; Flinn IW et al. | R/R iNHL (including R/R FL) | 129 (R/R FL 83) | Duvelisib | Two or more lines | PI3Kδ/γ inhibitor | 47.3% (R/R FL 42.2%) | 1.6 (R/R FL 1.2%) | 10 | 9.5 | 28.9 | ^4^ |
| Phase II; Younes A | R/R NHL (including R/R FL) | 72 (R/R FL 24) | Buparlisib | Two or more lines | Pan PI3K inhibitor | R/R FL 25% | R/R FL 0 | R/R FL 11 | R/R FL 9.8 | R/R FL NR | ^5^ |
| Phase IIb; Fowler NH | R/R iNHL (including R/R FL) | 208 (R/R FL 117) | Umbralisib | Two or more lines | PI3Kδ/CK1ε inhibitor | 47.1% (R/R FL 45.3%) | R/R FL 5.1% | R/R FL 11.1 | R/R FL 10.6 | - | ^6^ |
| Phase II; Morschhauser F et al. | R/R FL | 99 | Tazemetostat | Three or more lines | EZH2 inhibitor | 69% | 13% | 13 | 13.8 | NR | ^7^ |
| Phase II; Jacobson CA et al. | R/R iNHL (including R/R FL) | 148 (R/R FL 124) | Axicabtagene ciloleucel | Three or more lines | CAR-T-cell therapy | 92% (R/R FL 94%) | 74% (R/R FL 79%) | NR | NR | NR | ^8^ |
| Fowler NH et al. | R/R FL | 98 | Tisagenlecleucel | Three or more lines | CAR-T-cell therapy | 86.2% | 69.1% | NR | NR | NR | ^9^ |
| Phase II; Budde LE et al. | R/R FL | 90 | Mosunetuzumab | Three or more lines | Bispecific T-cell engager therapy | 80% | 60% | 22.8 | 17.9 | NA | ^10^ |

**Abbreviations:** PI3K, phosphatidylinositol 3-kinase; EZH2, enhancer of zeste homolog 2; CAR-T-cell, chimeric antigen receptor-T-cell; ORR, objective response rate; CRR, complete response rate; DOR, duration of response; mo, months; PFS, progression-free survival; OS, overall survival; NR, not reached; NA, not available; Ref, reference; iNHL, indolent non-Hodgkin lymphoma.

Table S2. Baseline characteristics of patients at run-in stage.

| **Characteristics** | **All patients (n=25)** |
| --- | --- |
| Male | 14 (56.0%) |
| Age, years-median (range) | 55.0 (49.0-61.0) |
| <60 | 18 (72.0%) |
| ≥60 | 7 (28.0%) |
| ECOG PS |  |
| 0 | 14 (56.0%) |
| 1 | 11 (44.0%) |
| Median time from initial diagnosis to start of study treatment, months (range) | 36.3 (6.6-96.1) |
| Histological grade |  |
| Grade 1-2 | 20 (80.0%) |
| Grade 3a | 5 (20.0%) |
| Relapsed case | 8 (32.0%) |
| Refractory case | 17 (68.0%) |
| FLIPI-2 |  |
| 0-1 | 9 (36.0%) |
| 2 | 5 (20.0%) |
| 3-5 | 11 (44.0%) |
| Lugano stage |  |
| I | 2 (8.0%) |
| III-IV | 18 (72.0%) |
| Other | 5 (20.0%) |
| Bone marrow involvement |  |
| Yes  No | 5 (20.0%)  20 (80.0%) |
| Prior radiotherapy |  |
| Yes | 3 (12.0%) |
| No | 22 (88.0%) |
| Organ or stem cell transplant |  |
| No | 25 (100.0%) |
| POD24 |  |
| Yes  No | 19 (76.0%)  6 (24.0%) |
| Lines of prior systemic therapies, median (range) | 3.0 (2.0-3.0) |
| 2 | 12 (48.0%) |
| 3 | 10 (40.0%) |
| >3 | 3 (12.0%) |
| Prior therapies |  |
| Rituximab | 25 (100.0%) |
| Alkylating agents | 23 (92.0%) |
| Immunomodulatory drugs | 16 (64.0%) |
| Bendamustine | 4 (16.0%) |
| BTK inhibitors | 0 |

**Abbreviations:** ECOG PS, Eastern Cooperative Oncology Group Performance Score; FLIPI-2, Follicular Lymphoma International Prognostic Index 2; POD24, progression of disease within 24 months; BTK, bruton tyrosine kinase.

Table S3. Efficacy data at run-in stage.

| **Efficacy** | **IRC-assessed (n=25)** | **Investigator-assessed (n=25)** |
| --- | --- | --- |
| CRR | 6 (24.0%, 9.4%-45.1%) | 5 (20.0%, 6.8%-40.7%) |
| PR | 16 (64.0%) | 17 (68.0%) |
| SD | 1 (4.0%) | 2 (8.0%) |
| PD | 2 (8.0%) | 1 (4.0%) |
| ORR | 22 (88.0%, 68.8%-97.5%) | 22 (88.0%, 68.8%-97.5%) |
| DCR | 23 (92.0%, 74.0%-99.0%) | 24 (96.0%, 79.7%-99.9%) |
| Median DOR, months (95% CI) | 11.8 (5.5-NE) | 14.8 (7.2-NE) |
| Median PFS, months (95% CI) | 12.0 (7.3-NE) | 10.9 (7.6-NE) |
| Median OS, months (95% CI) | Not reached | |
| 24-month OS rate (95% CI) | 78.9% (56.4%-90.6%) | |

Data were presented as n (%) or n (%, 95% confidence interval).

**Abbreviations:** NE, not estimable; ORR, objective response rate; DCR, disease control rate; DOR, duration of response; PFS, progression-free survival; OS, overall survival; IRC, independent review committee; CI, confidence interval.

Table S4. Summary of efficacy in the ITT at stage 2.

| **Efficacy** | **IRC-assessed (n=82)** | **Investigator-assessed (n=82)** |
| --- | --- | --- |
| Best response |  |  |
| CRR | 28 (34.2%, 24.0%-45.5%) | 32 (39.0%, 28.4%-50.4%) |
| PR | 43 (52.4%) | 40 (48.8%) |
| SD | 7 (8.5%) | 7 (8.5%) |
| PD | 2 (2.4%) | 1 (1.2%) |
| NE^*^ | 2 (2.4%) | 2 (2.4%) |
| ORR | 71 (86.6%, 77.3%-93.1%) | 72 (87.8%, 78.7%-94.0%) |
| DCR | 78 (95.1%, 88.0%-98.7%) | 79 (96.3%, 89.7%-99.2%) |
| Median DOR^#^, months (95% CI) | NR (9.2-NE) | 14.8 (9.2-20.4) |
| 6-month DOR rate (%, 95% CI) | 71.1% (56.9%-81.4%) | 73.8% (60.7%-83.2%) |
| 12-month DOR rate (%, 95% CI) | 60.2% (44.3%-72.9%) | 51.5% (36.0%-65.0%) |
| 18-month DOR rate (%, 95% CI) | 51.6% (30.6%-69.2%) | 41.0% (23.5%-57.7%) |
| Median PFS, months (95% CI) | 18.5 (10.2-NE) | 18.4 (11.0-22.0) |
| 6-month PFS rate (%, 95% CI) | 82.0% (70.9%-89.1%) | 81.4% (70.6%-88.5%) |
| 12-month PFS rate (%, 95% CI) | 58.3% (44.0%-70.1%) | 52.8% (38.8%-65.0%) |
| 18-month PFS rate (%,95% CI) | 58.3% (44.0%-70.1%) | 52.8% (38.8%-65.0%) |
| Median OS, months (95% CI) | Not reached | |
| 6-month OS rate (%, 95% CI) | 96.3% (89.1%-98.8%) | |
| 12-month OS rate (%, 95% CI) | 91.8% (82.5%-96.3%) | |
| 24-month OS rate (%, 95% CI) | 86.1% (72.3%-93.3%) | |
| Time to first response, months-median (range) | 1.8 (0.2-9.3) | 1.8 (0.2-7.3) |
| Time to first complete response, months-median (range) | 3.7 (1.8-11.2) | 3.7 (1.7-11.2) |

Data were presented as n (%) or n (%, 95% confidence interval).

^*^Cases 31001 and 32004 did not have post-baseline radiographic assessments and could not be evaluated for efficacy.

^#^ DOR was assessed in 71 patients achieving responses.

**Abbreviations:** CRR, complete response rate; PR, partial response; SD, stable disease; PD, progressive disease; NE, not estimable; NR, not reached; ORR, objective response rate; DCR, disease control rate; DOR, duration of response; PFS, progression-free survival; OS, overall survival; CI, confidence interval.

Table S5. Summary of efficacy in PPS^#^ at stage 2.

| **Efficacy** | **IRC-assessed (n=79)** | **Investigator-assessed (n=79)** |
| --- | --- | --- |
| Best response |  |  |
| CRR | 28 (35.4%, 25.0%-47.0%) | 32 (40.5%, 29.6%-52.2%) |
| PR | 42 (53.2%) | 39 (49.4%) |
| SD | 7 (8.9%) | 7 (8.9%) |
| PD | 2 (2.5%) | 1 (1.3%) |
| ORR | 70 (88.6%, 79.5%-94.7%) | 71 (89.9%, 81.0%-95.5%) |
| DCR | 77 (97.5%, 91.2%-99.7%) | 78 (98.7%, 93.2%-100.0%) |
| Median DOR^*^, months (95% CI) | NR (9.2-NE) | 14.8 (9.2-20.4) |
| 6-month DOR rate (%, 95% CI) | 71.1% (56.9%-81.4%) | 73.8% (60.7%-83.2%) |
| 12-month DOR rate (%, 95% CI) | 60.2% (44.3%-72.9%) | 51.5% (36.0%-65.0%) |
| 18-month DOR rate (%, 95% CI) | 51.6% (30.6%-69.2%) | 41.0% (23.5%-57.7%) |
| Median PFS, months (95% CI) | 18.5 (10.2-NE) | 18.4 (11.0-22.0) |
| 6-month PFS rate (%, 95% CI) | 82.0% (70.9%-89.1%) | 81.4% (70.6%-88.5%) |
| 12-month PFS rate (%, 95% CI) | 58.3% (44.0%-70.1%) | 52.8% (38.8%-65.0%) |
| 18-month PFS rate (%, 95% CI) | 58.3% (44.0%-70.1%) | 52.8% (38.8%-65.0%) |
| Median OS, months (95% CI) | Not reached | |
| 6-month OS rate (%, 95% CI) | 96.2% (88.7%-98.8%) | |
| 12-month OS rate (%, 95% CI) | 92.8% (83.4%-97.0%) | |
| 24-month OS rate (%, 95% CI) | 86.8% (72.4%-94.0%) | |
| Time to first response, months-median (range) | 1.8 (1.2-9.3) | 1.8 (1.2-7.3) |
| Time to first complete response, months-median (range) | 3.7 (1.8-11.2) | 3.7 (1.7-11.2) |

Data were presented as n (%) or n (%, 95% confidence interval).

^#^ PPS included 79 patients because 2 patients (cases 31001 and 32004) did not complete 1 cycle of treatment without efficacy evaluation results and case 42001 did not complete 1-cycle TQ-B3525 with efficacy evaluation.

^*^ DOR was assessed in 70 patients achieving responses.

**Abbreviations:** NE, not estimable; NR, not reached; CRR, complete response rate; PR, partial response; SD, stable disease; PD, progressive disease; ORR, objective response rate; DCR, disease control rate; DOR, duration of response; PFS, progression-free survival; OS, overall survival; IRC, independent review committee; CI, confidence interval.

Table S6. Summary of treatment exposure in SS at stage 2.

|  | **Safety population (n=82)** |
| --- | --- |
| TQ-B3525 exposure, cycles-median (range) | 8.5 (0-26) |
| TQ-B3525 exposure, months-median (range) | 7.5 (0.1-23.5) |
| ≤6 months | 33 (40.2%) |
| ≤12 months | 59 (72.0%) |
| Cumulative dose, mg-median (range) | 4050.0 (80.0-14300.0) |
| Dose intensity, mg/day-median (range) | 480.7 (310.6-615.4) |
| Relative dose intensity (RDI), %-median (range) | 79.0% (51.0%-100.0%) |
| <80%, n (%) | 44 (53.7%) |
| 80%-120%, n (%) | 38 (46.3%) |

**Abbreviations:** SS, safety analysis set.

Table S7. Summary of safety at run-in stage.

|  | **Patients (n=25)** | |
| --- | --- | --- |
| **Summary of TRAEs** | | |
| Any grade/≥grade 3 TRAEs | 25 (100.0%)/19 (76.0%) | |
| Serious TRAEs | 9 (36.0%) | |
| Any grade/≥grade 3 TRAEs leading to dose reduction | 17 (68.0%)/10 (40.0%) | |
| Any grade/≥grade 3 TRAEs leading to dose interruption | 17 (68.0%)/13 (52.0%) | |
| Any grade/≥grade 3 TRAEs leading to discontinuation | 2 (8.0%)/2 (8.0%) | |
| TRAEs leading to death | 0 | |
| **Summary of TEAEs** | | |
| Any grade/≥grade 3 TEAEs | 25 (100.0%)/19 (76.0%) | |
| Serious TEAEs | 14 (56.0%) | |
| Any grade/≥grade 3 TEAEs leading to dose reduction | 18 (72.0%)/10 (40.0%) | |
| Any grade/≥grade 3 TEAEs leading to dose interruption | 18 (72.0%)/15 (60.0%) | |
| Any grade/≥grade 3 TEAEs leading to discontinuation | 2 (8.0%)/2 (8.0%) | |
| TEAEs leading to death | 0 | |
| **Any-grade TRAEs with incidence of ≥10%** | | |
| **Events** | **Any grade** | **Grade 3 or higher** |
| Hyperglycemia | 19 (76.0%) | 7 (28.0%) |
| Neutropenia | 16 (64.0%) | 9 (36.0%) |
| Diarrhea | 15 (60.0%) | 3 (12.0%) |
| Leukopenia | 13 (52.0%) | 5 (20.0%) |
| Weight loss | 10 (40.0%) | 1 (4.0%) |
| Pneumonitis | 10 (40.0%) | 4 (16.0%) |
| Alanine aminotransferase increased | 9 (36.0%) | 0 |
| Thrombopenia | 8 (32.0%) | 2 (8.0%) |
| Lymphopenia | 7 (28.0%) | 2 (8.0%) |
| Glycosylated hemoglobin (A1c) elevated | 7 (28.0%) | 1 (4.0%) |
| Anemia | 6 (24.0%) | 1 (4.0%) |
| Glucosuria | 6 (24.0%) | 1 (4.0%) |
| Occult blood positive | 5 (20.0%) | 0 |
| Aspartate aminotransferase increased | 5 (20.0%) | 1 (4.0%) |
| Lipase increased | 5 (20.0%) | 0 |
| Hypokalemia | 5 (20.0%) | 1 (4.0%) |
| Nausea | 5 (20.0%) | 0 |
| Fever | 5 (20.0%) | 0 |
| Infectious pneumonia | 5 (20.0%) | 4 (16.0%) |
| Mucositis oral | 4 (16.0%) | 2 (8.0%) |
| Proteinuria | 4 (16.0%) | 0 |
| Dizziness | 4 (16.0%) | 0 |
| Fatigue | 4 (16.0%) | 0 |
| Decreased appetite | 4 (16.0%) | 0 |
| Hypertriglyceridemia | 4 (16.0%) | 0 |
| Blood bilirubin increased | 4 (16.0%) | 0 |
| Creatinine increased | 4 (16.0%) | 0 |
| Positive urinary ketone body | 3 (12.0%) | 0 |
| Hypoalbuminemia | 3 (12.0%) | 0 |
| Hypercholesterolemia | 3 (12.0%) | 0 |
| Oral ulcer | 3 (12.0%) | 0 |
| Vomiting | 3 (12.0%) | 0 |

Data were presented as n (%).

**Abbreviations:** TEAEs, Treatment-emergent adverse events; TRAEs, treatment-related adverse events.

Table S8. TRAEs of special interest in SS at stage 2.

|  | **Safety population (n=82)** | |
| --- | --- | --- |
|  | **Any grade** | **Grade 3 or higher** |
| **TRAEs of special interest** | **82 (100.0%)** | **50 (61.0%)** |
| \| Hyperglycemia \| \| --- \| | 68 (82.9%) | 16 (19.5%) |
| Diarrhea | 46 (56.1%) | 11 (13.4%) |
| Neutropenia | 37 (45.1%) | 18 (22.0%) |
| Thrombopenia | 30 (36.6%) | 7 (8.5%) |
| Lymphopenia | 26 (31.7%) | 7 (8.5%) |
| Pneumonitis | 13 (15.9%) | 5 (6.1%) |
| Infectious pneumonia | 12 (14.6%) | 6 (7.3%) |
| Upper respiratory infection | 8 (9.8%) | 3 (3.7%) |
| Interstitial lung disease | 4 (4.9%) | 2 (2.4%) |
| Diabetes | 2 (2.4%) | 2 (2.4%) |
| Diabetic ketoacidosis | 1 (1.2%) | 0 |
| Sepsis | 1 (1.2%) | 1 (1.2%) |

Data were presented as n (%).

**Abbreviations:** TRAEs, treatment-related adverse events.

Table S9. Summary of safety in SS at stage 2.

|  | **Safety population (n=82)** |
| --- | --- |
| **Summary of TRAEs** | |
| Any grade/≥grade 3 TRAEs | 82 (100.0%)/63 (76.8%) |
| Serious TRAEs | 39 (47.6%) |
| TRAEs leading to dose reduction (any grade/≥grade 3) | 61 (74.4%)/37 (45.1%) |
| TRAEs leading to dose interruption (any grade/≥grade 3) | 60 (73.2%)/41 (50.0%) |
| TRAEs leading to discontinuation (any grade/≥grade 3) | 8 (9.8%)/4 (4.9%) |
| TRAEs leading to death | 1 (1.2%) |
| **Summary of TEAEs** | |
| Any grade/≥grade 3 TEAEs | 82 (100.0%)/67 (81.7%) |
| Serious TEAEs | 46 (56.1%) |
| TEAEs leading to dose reduction (any grade/≥grade 3) | 63 (76.8%)/37 (45.1%) |
| TEAEs leading to dose suspension (any grade/≥grade 3) | 64 (78.1%)/43 (52.4%) |
| TEAEs leading to discontinuation (any grade/≥grade 3) | 8 (9.8%)/4 (4.9%) |
| TEAEs leading to death | 1 (1.2%) |

Data were presented as n (%).

**Abbreviations:** TEAEs, treatment-emergent adverse events; TRAEs, treatment-related adverse events.

Table S10. One death due to sepsis at stage 2.

The investigators considered that sepsis was associated with disease progression or may be related to study drug TQ-B3525.

| **Time** | **Process** |
| --- | --- |
| Baseline | Presence of ruptured neck mass. |
| 2022-2-13 | The patient was admitted to the emergency department due to a fall and respiratory distress. Upon examination, there were indications of local skin breakdown with increased exudate, accompanied by foul-smelling purulent discharge. Blood cultures were positive for Gram-negative bacteria. Tumor progression was suspected, with worsening infection at the tumor site and skin breakdown, leading to sepsis complicated by pulmonary infection and respiratory failure. |
| 2022-2-15 | After receiving antibiotic treatment without any improvement, the patient's condition did not improve, and the family decided to discontinue treatment and have the patient discharged. |
| 2022-2-18 | Die. |

Table S11. Treatment-emergent adverse events (TEAEs) with incidence of ≥10% in SS at stage 2.

|  | **Safety population (n=82)** | |
| --- | --- | --- |
|  | **Any grade** | **Grade 3 or higher** |
| **TEAEs** | **82 (100.0%)** | **67 (81.7%)** |
| \| Hyperglycemia \| \| --- \| | 68 (82.9%) | 16 (19.5%) |
| Diarrhea | 50 (61.0%) | 13 (15.9%) |
| Neutropenia | 38 (46.3%) | 18 (22.0%) |
| Weight loss | 36 (43.9%) | 2 (2.4%) |
| Hypokalemia | 34 (41.5%) | 10 (12.2%) |
| Thrombopenia | 34 (41.5%) | 8 (9.8%) |
| Lymphopenia | 32 (39.0%) | 8 (9.8%) |
| Leukopenia | 31 (37.8%) | 10 (12.2%) |
| Anemia | 27 (32.9%) | 4 (4.9%) |
| Hypoalbuminemia | 20 (24.4%) | 0 |
| Decreased appetite | 19 (23.2%) | 3 (3.7%) |
| Blood lactate dehydrogenase increased | 19 (23.2%) | 0 |
| Blood bilirubin increased | 18 (22.0%) | 0 |
| Vomiting | 17 (20.7%) | 1 (1.2%) |
| Glycosylated hemoglobin (A1c) elevated | 17 (20.7%) | 2 (2.4%) |
| Proteinuria | 16 (19.5%) | 0 |
| Upper respiratory infection | 16 (19.5%) | 4 (4.9%) |
| Alanine aminotransferase increased | 16 (19.5%) | 2 (2.4%) |
| Fever | 16 (19.5%) | 0 |
| Increased C-reactive protein | 15 (18.3%) | 3 (3.7%) |
| Pneumonitis | 14 (17.1%) | 5 (6.1%) |
| Nausea | 14 (17.1%) | 0 |
| Hyponatremia | 14 (17.1%) | 1 (1.2%) |
| Glucosuria | 14 (17.1%) | 1 (1.2%) |
| Positive urinary ketone body | 14 (17.1%) | 0 |
| Creatinine increased | 14 (17.1%) | 1 (1.2%) |
| Fatigue | 13 (15.9%) | 3 (3.7%) |
| Lung infection | 13 (15.9%) | 7 (8.5%) |
| Cough | 12 (14.6%) | 0 |
| Urinary tract infection | 12 (14.6%) | 0 |
| Aspartate aminotransferase increased | 12 (14.6%) | 2 (2.4%) |
| Lipase increased | 12 (14.6%) | 4 (4.9%) |
| Hypocalcemia | 11 (13.4%) | 0 |
| CD4 lymphocytes decreased | 11 (13.4%) | 5 (6.1%) |
| Hypertriglyceridemia | 10 (12.2%) | 0 |
| Lymphocyte count increased | 10 (12.2%) | 0 |
| Occult blood positive | 10 (12.2%) | 0 |
| Elevated conjugated bilirubin | 9 (11.0%) | 0 |
| Headache | 9 (11.0%) | 0 |

Data were presented as n (%).

**Abbreviations:** TEAEs, treatment-emergent adverse events.

Table S12. Detailed eligibility criteria.

| **Inclusion criteria** |
| --- |
| Written informed consent for all study procedures prior to the study; |
| Age of at least 18 years old; |
| ECOG PS of ≤2; |
| Life expectancy of ≥3 months; |
| Histopathologically confirmed grade 1-3a FL; |
| Relapsed^*^ and/or refractory^#^ FL after two or more lines of systemic therapy (at least 1 regimen containing rituximab or anti-CD20 monoclonal antibody); |
| At least one radiologically measurable malignant lesion of lymph nodes and extranodal lymphoma as evaluated by CT or MRI; |
| Adequate organ function: |
| Blood routine test (no use of growth factor or transfusion within 7 days): |
| Absolute neutrophil count (ANC) ≥1.5×10^9^/L; |
| Lymphocyte count (LYM) ≥0.5×10^9^/L; |
| CD4+ T cell count ≥0.2×10^9^/L; |
| Platelets (PLT) ≥75×10^9^/L (lymphoma patients with bone marrow infiltration ≥50×10^9^/L were allowed); |
| Hemoglobin (Hb) ≥80 g/L; |
| Biochemistry test: |
| ALT and AST ≤2.5×ULN (≤5×ULN if the patient had liver metastases or biliary obstruction); |
| Total bilirubin (TBIL) ≤1.5×ULN; |
| Creatinine (Cr) ≤1.5×ULN or creatinine clearance ≥50 mL/min; |
| Coagulation function test: |
| Activated partial thromboplastin time (APTT), international normalized ratio (INR), and prothrombin time ≤1.5×ULN; |
| Male or female subjects should agree to use an adequate method of contraception starting with the first dose of study therapy through 6 months after the last dose of study (such as intrauterine devices, contraceptives or condoms); no pregnant or breastfeeding women, and a negative pregnancy test are received within 7 days before the first administration; |
| **Exclusion criteria** |
| Known FL transforming to diffuse large B-cell lymphoma; |
| Active central nervous system lymphoma; |
| Prior treatments with PI3K inhibitors or CAR-T; |
| Has diagnosed and/or treated additional malignancy within 3 years prior to the first administration (other than cured cervical carcinoma in situ, non-melanoma skin cancer, or superficial bladder tumor [Ta, non-invasive; Tis, carcinoma in situ; T1, superficially invasive]); |
| Type I and Type II diabetes, except those who meet one of the following conditions: |
| Type II diabetes patients with only exercise and diet control and fasting blood glucose <7.0 mmol/L and glycosylated hemoglobin (HbA1c) <7.0% at the screening; |
| Type II diabetes patients who could stably control the blood glucose by a single oral hypoglycemic agent, and were tested fasting blood glucose of <7.0 mmol/L and the glycosylated hemoglobin (HbA1c) of <7.0% at the screening; |
| History of interstitial lung disease, severely impaired lung function, severe pulmonary fibrosis, and drug-induced lung disease (determined by investigators); and active pneumonia confirmed by CT at the screening; |
| Infection requiring systemic therapy (prophylactic medication if no active infection); |
| Cytomegalovirus (CMV) infection (CMV PCR test positive at the screening); |
| History of immunodeficiency diseases (HIV-positive; acquired and congenital immunodeficiency diseases; history or active autoimmune disease); |
| Multiple factors affecting oral medication (ie. swallowing difficulty, gastrointestinal resection, ulcerative colitis, symptomatic/inflammatory bowel disease, chronic diarrhea, and intestinal obstruction); |
| Adverse events caused by previous therapy except alopecia that did not recover to ≤grade 1; |
| Systemic steroid treatment within 7 days before the first administration (prednisone equivalent dose of >10 mg/day or its equivalent); the followings were except: (1) topical, intraocular, intra-articular, intranasal, or inhaled corticosteroids were permitted; (2) short-term use of corticosteroids is permitted for prevention (such as allergy to contrast agents) or treatment of non-autoimmune conditions (such as delayed hypersensitivity reactions caused by exposure to allergens); |
| Other systemic anti-tumor medications within 4 weeks before the first administration, or still within the 5 half-life of the medication, which occurred first; |
| Major surgery, or unhealed wounds within 4 weeks before the first administration; |
| Tendency or history of bleeding diathesis; bleeding of ≥ grade 3 (ie. gastrointestinal bleeding and perforation) within 4 weeks before the first administration; |
| History of autologous hematopoietic stem cell transplant within 3 months or allogeneic hematopoietic stem cell transplant or organ transplantation (except corneal transplantation); |
| Cardiovascular disease ≥grade 2 within 6 months before the first administration: unstable angina, myocardial infarction, arrhythmia requiring treatment, congestive heart failure, and cerebrovascular accidents (including transient ischemic attacks); |
| QTc >480ms; left ventricular ejection fraction (LVEF) <50%; |
| Urinary protein ≥++, and confirmed 24-hour urinary protein >1.0 g within 7 days; |
| Epidemiological detection:  HBsAg positive and HBV DNA exceeding the upper limit of normal value (those who fall within the normal range after antiviral treatment could be included); |
| Anti-HCV positive; |
| Psychotropic substances abuse or a mental disorder; |
| Other conditions that make it inappropriate for the patient to be enrolled based on investigator's opinion; |

**Abbreviations:** ECOG PS, Eastern Cooperative Oncology Group Performance Status; FL, follicular lymphoma; CT, computed tomography; MRI, magnetic resonance imaging; ULN, upper limit of normal; HBV, hepatitis B virus; HCV, hepatitis C virus; DNA, deoxyribonucleic acid; PCR, polymerase chain reaction.

^*^Relapse was defined as disease progression following remissions with adequate treatment; at least one prior regimen must have contained an rituximab.

^#^ Refractoriness was defined per protocol as being unresponsive to a rituximab-containing regimen or progressing within the therapy or 6 months after completion of treatment.

Table S13. Detailed dose titration criteria.

| Patients were required to swallow the tablet whole; the treatments like breaking it apart and dissolving it in liquid were not permitted before administration: | | |
| --- | --- | --- |
| If patients received additional doses of TQ-B3525 within 1 day, skip a subsequent dose; | | |
| If patients were vomiting at therapeutic doses of TQ-B3525 on the same day, re-administration of vomited doses were not allowed; | | |
| Patients did not supplement a missed dose in the <12 hours prior to the next dose; | | |
| The criteria of treatment regimen modification: | | |
| The treatment should be adjusted based on the most-severe toxicities observed when toxicities varying the severity occurred simultaneously; | | |
| Treatment was discontinued if the cumulative duration of dosing interruption due to toxicities for more than 4 weeks; patients were considered to continue the study after communicating with the sponsor and the main investigator, and obtaining the consent of the patients; | | |
| Delayed administration was required if treatment-related adverse events did not restore to ≤grade 2 or baseline (except for alopecia) before any cycle of administration; investigators should carefully consider the rationality of treatment in exceptional cases beyond the protocol and made detailed records; | | |
| Dose reductions were permitted if clinically significant toxicities were observed; if a subject had experienced two dose reductions, the treatment must be discontinued when a third reduction was required due to toxicities; re-escalation was not permitted; | | |
| **Hematologic toxicities** | | |
| **Toxicities** | **Dose modification** | **Recommended treatment** |
| - Grade 3 hemoglobin decreased; | - Delay delivery till toxicities restored to grade ≤1 or baseline; | - Symptomatic treatment and monitoring at least weekly until toxicity recovered |
| - Grade 3 neutrophil count decreased; | - Continue medication after 1 dose reduction; |  |
| - Grade 2 platelet count decreased (normal baseline); | - Normal dose when grade 2 platelet count decreased (normal baseline) restored to grade ≤1; |  |
| - Grade 2 platelet count decreased (baseline <LLN); |  |  |
| - Grade 3 lymphocyte count decreased; | - Delay delivery till toxicities restored to grade ≤2 or baseline; | - Symptomatic treatment and monitoring at least weekly until toxicity recovered; |
| - Grade 3 CD4-positive lymphocytes decreased; | - Continue medication after 1 dose reduction; | - Sulfonamides for prevention of opportunistic infections caused by Pneumocystis jirovecii pneumonia; |
| - Grade 4 hemoglobin decreased; | - Delay delivery till toxicities restored to grade ≤2 or baseline; | - Symptomatic treatment and monitoring at least weekly until toxicity recovered |
| - Grade 4 neutrophil count decreased; | - Continue medication after 1-2 dose reductions or discontinuation at the discretion of the investigator; |  |
| - Grade 4 platelet count decreased; |  |  |
| - Grade ≥3 febrile neutropenia; |  |  |
| - Grade ≥3 platelet count decreased with bleeding; |  |  |
| **Increased** **fasting blood-glucose (FBG)** | | |
| **Change of FBG (mmol/L)** | **Dose modification** | **Recommended treatment** |
| - ULN≤FBG≤8.9 | - Normal dose; | - Continuous observation and oral hypoglycemic drugs can be given for symptomatic treatment; - If blood glucose levels continue to rise, prophylactic use of oral hypoglycemic agents may be considered during subsequent treatment; |
| - 8.9<FBG≤13.9 | - Investigators could determine whether to delay dosing based on both fasting and postprandial blood glucose levels; - Normal dose or continue medication after 1 dose reduction; | - Oral hypoglycemic drugs were given for symptomatic treatment; combined oral hypoglycemic agents with different mechanisms of action could be given following no use with single hypoglycemic agents, and insulin is used when necessary, and monitoring is repeated within 7 days until FBG ≤8.9; - Focus on the changes of urinary ketone and other indicators simultaneously; - If blood glucose levels continue to rise, prophylactic use of oral hypoglycemic agents may be considered during subsequent treatment; |
| - FBG>13.9 and no complications (duration <3 days); | - Delay delivery till FBG ≤8.9; - Investigators could determine whether to reduce 1 dose or maintain the normal dose; | - Symptomatic treatment and monitoring is repeated within 7 days until FBG ≤8.9; - Closely monitor the changes of FBG, urine ketone, and other indicators; - Prophylactic use of oral hypoglycemic agents or insulin may be considered during subsequent treatment; |
| - FBG>13.9 and no complications (duration ≥3 days); | - Delay delivery till FBG ≤8.9; - Investigators could determine whether to reduce 1-2 dose; |  |
| - FBG>13.9 with complications; | - Discontinuation; | - Withdrawal from the study after symptomatic treatment; |
| **Infections** | | |
| **Grade** | **Dose modification** | **Recommended treatment** |
| 1 | - Normal dose; | - Continuous observation and use of prophylactic drugs; |
| 2 | - Delay delivery till toxicities restored to grade ≤1; - Investigators could determine whether to reduce 1-2 doses; | - Symptomatic treatment; |
| 3 | - Delay delivery till toxicities restored to grade ≤1; - Investigators could determine whether to reduce 1-2 doses or discontinue the treatment; | - Symptomatic treatment; |
| 4 | - Discontinuation; | - Withdrawal from the study after symptomatic treatment; |
| - Discontinuation of TQ-B3525 and symptomatic treatment immediately when Pneumocystis jirovecii pneumonia occurred; - Patients with CMV (Cytomegalovirus) infection, herpes simplex virus infection, and varicella zoster infection of any grade, should immediately discontinueTQ-B3525 and receive symptomatic treatment until the infection subsides; then, investigators could determine whether to reduce dose or maintain the normal dose; | | |
| **Noninfectious pneumonia (NIP)** | | |
| **Grade** | **Dose modification** | **Recommended treatment** |
| 1 | - Normal dose; | - Continuous observation and symptomatic treatment; |
| 2 | - Delay delivery till toxicities restored to grade ≤1; - Investigators could determine whether to reduce 1-2 doses; | - Symptomatic treatment; |
| 3-4 | - Discontinuation; | - Withdrawal from the study after symptomatic treatment; |
| - In principle, treatment should be discontinued after the grade 3 non-infectious pneumonia; if the patient still requested to continue the treatment, the investigator should determine to reduce 1-2 doses for restarting the treatment after assessing the potential benefits and communicating with the sponsor and PI and record the reasons in detail in the original medical record; | | |
| **Gastrointestinal adverse events (diarrhea/enteritis/nausea/vomiting)** | | |
| **Grade** | **Dose modification** | **Recommended treatment** |
| 1-2 | - Normal dose; | - prophylactic treatment (grade 1-2) or symptomatic treatment (grade 3-4) at first occurrence; - If diarrhea persists after prophylactic or symptomatic treatment, it should be evaluated for the possibility of concomitant medication (e.g. metformin), gastrointestinal infection (including CMV, Clostridium difficile, etc.), or gastrointestinal inflammation (through endoscopy or tissue examination); |
| 3-4 | - Delay delivery till toxicities restored to grade ≤2 and reduce 1 dose at first occurrence; - Investigators could determine whether to reduce 1-2 doses or discontinue the treatment if diarrhea, grade ≥3 diarrhea, or its complications occurred later; |  |
| **Alanine transaminase (ALT)/** a**spartate aminotransferase (AST) increased with total bilirubin increased grade ≤1** | | |
| **Grade (ALT/AST)** | **Dose modification** | **Recommended treatment** |
| 1-2 | - Normal dose; | - Continuous observation and investigators determine whether to proceed with liver protection therapy; - If accompanied by clinical symptoms, repeat monitoring toxicity to grade ≤1 within 7 days after treatment; |
| 3-4 | - Delay delivery till toxicities restored to grade ≤1; - Continue medication after 1 dose reduction; | - Liver protection therapy and repeat monitoring toxicity to grade ≤1 within 7 days; |
| **ALT/AST increased with total bilirubin increased grade ≥2 (no cholestasis or hemolysis)** | | |
| **Grade (ALT/AST)** | **Dose modification** | **Recommended treatment** |
| 1 | - Normal dose; | - Repeat monitoring within 48 hours until normal or abnormal was not clinically significant; |
| 2 | - Delay delivery till toxicities restored to grade ≤1; - Continue medication after 1 dose reduction; | - Liver protection therapy and repeat monitoring toxicity to grade ≤1 within 7 days; |
| 3-4 | - Discontinuation; | - Withdrawal from the study after symptomatic treatment; |
| **Other non-hematological adverse events** | | |
| **Grade** | **Dose modification** | **Recommended treatment** |
| 1 | - Normal dose; | - Continuous observation and investigators determine whether to proceed with symptomatic treatment; |
| 2-3 | - Investigators could determine whether to delay delivery till toxicities restored to grade ≤1 or baseline; - Investigators could determine whether to reduce 1-2 doses or discontinue the treatment; | - Continuous observation, symptomatic treatment, and repeat monitoring within 7 days; |
| 4 | - Discontinuation; | - Withdrawal from the study after symptomatic treatment; |

**Abbreviations:** LLN, lower limit of the normal; FBG, fasting blood-glucose; ULN, upper limit of normal; CMV, cytomegalovirus; NIP, noninfectious pneumonia; ALT, alanine transaminase; AST, aspartate aminotransferase.

References

1 Dreyling M. *et al.* Phosphatidylinositol 3-Kinase Inhibition by Copanlisib in Relapsed or Refractory Indolent Lymphoma. *J Clin Oncol*. **35**, 3898-3905 (2017).

2 Gopal A. K. *et al.* PI3Kδ inhibition by idelalisib in patients with relapsed indolent lymphoma. *N Engl J Med*. **370**, 1008-1018 (2014).

3 Wang T. *et al.* The Oral PI3Kδ Inhibitor Linperlisib for the Treatment of Relapsed and/or Refractory Follicular Lymphoma: A Phase II, Single-Arm, Open-Label Clinical Trial. *Clin Cancer Res*. **29**, 1440-1449 (2023).

4 Flinn I. W. *et al.* DYNAMO: A Phase II Study of Duvelisib (IPI-145) in Patients With Refractory Indolent Non-Hodgkin Lymphoma. *J Clin Oncol*. **37**, 912-922 (2019).

5 Younes A. *et al.* Pan-phosphatidylinositol 3-kinase inhibition with buparlisib in patients with relapsed or refractory non-Hodgkin lymphoma. *Haematologica*. **102**, 2104-2112 (2017).

6 Fowler N. H. *et al.* Umbralisib, a Dual PI3Kδ/CK1ε Inhibitor in Patients With Relapsed or Refractory Indolent Lymphoma. *J Clin Oncol*. **39**, 1609-1618 (2021).

7 Morschhauser F. *et al.* Tazemetostat for patients with relapsed or refractory follicular lymphoma: an open-label, single-arm, multicentre, phase 2 trial. *Lancet Oncol*. **21**, 1433-1442 (2020).

8 Jacobson C. A. *et al.* Axicabtagene ciloleucel in relapsed or refractory indolent non-Hodgkin lymphoma (ZUMA-5): a single-arm, multicentre, phase 2 trial. *Lancet Oncol*. **23**, 91-103 (2022).

9 Fowler N. H. *et al.* Tisagenlecleucel in adult relapsed or refractory follicular lymphoma: the phase 2 ELARA trial. *Nat Med*. **28**, 325-332 (2022).

10 Budde L. E. *et al.* Safety and efficacy of mosunetuzumab, a bispecific antibody, in patients with relapsed or refractory follicular lymphoma: a single-arm, multicentre, phase 2 study. *Lancet Oncol*. **23**, 1055-1065 (2022).
